# Genetic determinants of telomere length from 109,122 ancestrally diverse whole-genome sequences in TOPMed

**DOI:** 10.1016/j.xgen.2021.100084

**Published:** 2022-01-13

**Authors:** Margaret A. Taub, Matthew P. Conomos, Rebecca Keener, Kruthika R. Iyer, Joshua S. Weinstock, Lisa R. Yanek, John Lane, Tyne W. Miller-Fleming, Jennifer A. Brody, Laura M. Raffield, Caitlin P. McHugh, Deepti Jain, Stephanie M. Gogarten, Cecelia A. Laurie, Ali Keramati, Marios Arvanitis, Albert V. Smith, Benjamin Heavner, Lucas Barwick, Lewis C. Becker, Joshua C. Bis, John Blangero, Eugene R. Bleecker, Esteban G. Burchard, Juan C. Celedón, Yen Pei C. Chang, Brian Custer, Dawood Darbar, Lisa de las Fuentes, Dawn L. DeMeo, Barry I. Freedman, Melanie E. Garrett, Mark T. Gladwin, Susan R. Heckbert, Bertha A. Hidalgo, Marguerite R. Irvin, Talat Islam, W. Craig Johnson, Stefan Kaab, Lenore Launer, Jiwon Lee, Simin Liu, Arden Moscati, Kari E. North, Patricia A. Peyser, Nicholas Rafaels, Christine Seidman, Daniel E. Weeks, Fayun Wen, Marsha M. Wheeler, L. Keoki Williams, Ivana V. Yang, Wei Zhao, Stella Aslibekyan, Paul L. Auer, Donald W. Bowden, Brian E. Cade, Zhanghua Chen, Michael H. Cho, L. Adrienne Cupples, Joanne E. Curran, Michelle Daya, Ranjan Deka, Celeste Eng, Tasha E. Fingerlin, Xiuqing Guo, Lifang Hou, Shih-Jen Hwang, Jill M. Johnsen, Eimear E. Kenny, Albert M. Levin, Chunyu Liu, Ryan L. Minster, Take Naseri, Mehdi Nouraie, Muagututi‘a Sefuiva Reupena, Ester C. Sabino, Jennifer A. Smith, Nicholas L. Smith, Jessica Lasky-Su, James G. Taylor, Marilyn J. Telen, Hemant K. Tiwari, Russell P. Tracy, Marquitta J. White, Yingze Zhang, Kerri L. Wiggins, Scott T. Weiss, Ramachandran S. Vasan, Kent D. Taylor, Moritz F. Sinner, Edwin K. Silverman, M. Benjamin Shoemaker, Wayne H.-H. Sheu, Frank Sciurba, David A. Schwartz, Jerome I. Rotter, Daniel Roden, Susan Redline, Benjamin A. Raby, Bruce M. Psaty, Juan M. Peralta, Nicholette D. Palmer, Sergei Nekhai, Courtney G. Montgomery, Braxton D. Mitchell, Deborah A. Meyers, Stephen T. McGarvey, Fernando D. Martinez on behalf of the NHLBI CARE Network, Angel C.Y. Mak, Ruth J.F. Loos, Rajesh Kumar, Charles Kooperberg, Barbara A. Konkle, Shannon Kelly, Sharon L.R. Kardia, Robert Kaplan, Jiang He, Hongsheng Gui, Frank D. Gilliland, Bruce D. Gelb, Myriam Fornage, Patrick T. Ellinor, Mariza de Andrade, Adolfo Correa, Yii-Der Ida Chen, Eric Boerwinkle, Kathleen C. Barnes, Allison E. Ashley-Koch, Donna K. Arnett, Christine Albert, NHLBI Trans-Omics for Precision Medicine (TOPMed) Consortium, TOPMed Hematology and Hemostasis Working Group, TOPMed Structural Variation Working Group, Cathy C. Laurie, Goncalo Abecasis, Deborah A. Nickerson, James G. Wilson, Stephen S. Rich, Daniel Levy, Ingo Ruczinski, Abraham Aviv, Thomas W. Blackwell, Timothy Thornton, Jeff O’Connell, Nancy J. Cox, James A. Perry, Mary Armanios, Alexis Battle, Nathan Pankratz, Alexander P. Reiner, Rasika A. Mathias

**Affiliations:** 1 Department of Biostatistics, Johns Hopkins Bloomberg School of Public Health, Baltimore, MD, USA; 2 Department of Biostatistics, School of Public Health, University of Washington, Seattle, WA, USA; 3 Department of Biomedical Engineering, Johns Hopkins Whiting School of Engineering, Baltimore, MD, USA; 4 Department of Epidemiology, Johns Hopkins Bloomberg School of Public Health, Baltimore, MD, USA; 5 Department of Biostatistics, University of Michigan School of Public Health, Ann Arbor, MI, USA; 6 Center for Statistical Genetics, University of Michigan School of Public Health, Ann Arbor, MI, USA; 7 GeneSTAR Research Program, Department of Medicine, Johns Hopkins School of Medicine, Baltimore, MD, USA; 8 Department of Laboratory Medicine & Pathology, University of Minnesota, Minneapolis, MN, USA; 9 Department of Medicine, Division of Genetic Medicine, Vanderbilt University Medical Center, Nashville, TN, USA; 10 Cardiovascular Health Research Unit, Department of Medicine, University of Washington, Seattle, WA, USA; 11 Department of Genetics, University of North Carolina, Chapel Hill, Chapel Hill, NC, USA; 12 Department of Cardiology, Johns Hopkins School of Medicine, Baltimore, MD, USA; 13 Department of Medicine, Division of Cardiology, Johns Hopkins School of Medicine, Baltimore, MD, USA; 14 LTRC Data Coordinating Center, The Emmes Company, LLC, Rockville, MD, USA; 15 Department of Human Genetics and South Texas Diabetes and Obesity Institute, University of Texas Rio Grande Valley School of Medicine, Brownsville, TX, USA; 16 Department of Medicine, Division of Genetics, Genomics, and Precision Medicine, University of Arizona, Tucson, AZ, USA; 17 Division of Pharmacogenomics, University of Arizona, Tucson, AZ, USA; 18 Department of Medicine, University of California, San Francisco, San Francisco, CA, USA; 19 Department of Bioengineering and Therapeutic Sciences, University of California, San Francisco, San Francisco, CA, USA; 20 Division of Pediatric Pulmonary Medicine, UPMC Children’s Hospital of Pittsburgh, University of Pittsburgh, Pittsburgh, PA, USA; 21 Department of Medicine, University of Maryland School of Medicine, Baltimore, MD, USA; 22 Vitalant Research Institute, San Francisco, CA, USA; 23 Department of Laboratory Medicine, University of California, San Francisco, San Francisco, CA, USA; 24 Division of Cardiology, University of Illinois at Chicago, Chicago, IL, USA; 25 Cardiovascular Division, Department of Medicine, Washington University School of Medicine in St. Louis, St. Louis, MO, USA; 26 Channing Division of Network Medicine, Department of Medicine, Brigham and Women’s Hospital, Boston, MA, USA; 27 Harvard Medical School, Boston, MA, USA; 28 Department of Internal Medicine, Section on Nephrology, Wake Forest School of Medicine, Winston-Salem, NC, USA; 29 Department of Medicine and Duke Comprehensive Sickle Cell Center, Duke University Medical Center, Durham, NC, USA; 30 Duke Molecular Physiology Institute, Duke University Medical Center, Durham, NC, USA; 31 Department of Medicine, University of Pittsburgh School of Medicine, Pittsburgh, PA, USA; 32 Cardiovascular Health Research Unit and Department of Epidemiology, University of Washington, Seattle, WA, USA; 33 Kaiser Permanente Washington Health Research Institute, Seattle, WA, USA; 34 Department of Epidemiology, University of Alabama at Birmingham, Birmingham, AL, USA; 35 Division of Environmental Health, Department of Population and Public Health Sciences, University of Southern California, Los Angeles, CA, USA; 36 Department of Biostatistics, Collaborative Health Studies Coordinating Center, University of Washington, Seattle, WA, USA; 37 Department of Medicine I, University Hospital Munich, Ludwig-Maximilian’s University, Munich, Germany; 38 German Centre for Cardiovascular Research (DZHK), partner site Munich Heart Alliance, Munich, Germany; 39 Laboratory of Epidemiology and Population Science, National Institute on Aging, National Institutes of Health, Bethesda, MD, USA; 40 Department of Medicine, Division of Sleep and Circadian Disorders, Brigham and Women’s Hospital, Boston, MA, USA; 41 Department of Epidemiology and Brown Center for Global Cardiometabolic Health, Brown University, Providence, RI, USA; 42 The Charles Bronfman Institute for Personalized Medicine, Icahn School of Medicine at Mount Sinai, New York, NY, USA; 43 Department of Epidemiology, University of North Carolina, Chapel Hill, Chapel Hill, NC, USA; 44 Department of Epidemiology, University of Michigan School of Public Health, Ann Arbor, MI, USA; 45 Department of Medicine, University of Colorado Denver, Anschutz Medical Campus, Aurora, CO, USA; 46 Department of Genetics, Harvard Medical School, Boston, MA, USA; 47 Department of Human Genetics, Graduate School of Public Health, University of Pittsburgh, Pittsburgh, PA, USA; 48 Department of Biostatistics, Graduate School of Public Health, University of Pittsburgh, Pittsburgh, PA, USA; 49 Center for Sickle Cell Disease and Department of Medicine, College of Medicine, Howard University, Washington, DC 20059, USA; 50 Department of Genome Sciences, University of Washington, Seattle, WA, USA; 51 Center for Individualized and Genomic Medicine Research (CIGMA), Department of Internal Medicine, Henry Ford Health System, Detroit, MI, USA; 52 Zilber School of Public Health, University of Wisconsin, Milwaukee, Milwaukee, WI, USA; 53 Department of Biochemistry, Wake Forest School of Medicine, Winston-Salem, NC, USA; 54 Division of Sleep Medicine, Department of Medicine, Brigham and Women’s Hospital, Boston, MA, USA; 55 Department of Biostatistics, Boston University School of Public Health, Boston, MA, USA; 56 The National Heart, Lung, and Blood Institute, Boston University’s Framingham Heart Study, Framingham, MA, USA; 57 Department of Environmental and Public Health Sciences, University of Cincinnati, Cincinnati, OH, USA; 58 Center for Genes, Environment, and Health, National Jewish Health, Denver, CO, USA; 59 Department of Biostatistics and Informatics, University of Colorado, Denver, Aurora, CO, USA; 60 The Institute for Translational Genomics and Population Sciences, Department of Pediatrics, The Lundquist Institute for Biomedical Innovation at Harbor-UCLA Medical Center, Torrance, CA, USA; 61 Department of Preventive Medicine, Northwestern University, Chicago, IL, USA; 62 Population Sciences Branch, Division of Intramural Research, National Heart, Lung, and Blood Institute, National Institutes of Health, Bethesda, MD, USA; 63 Bloodworks Northwest Research Institute, Seattle, WA, USA; 64 University of Washington, Department of Medicine, Seattle, WA, USA; 65 Center for Genomic Health, Icahn School of Medicine at Mount Sinai, New York, NY, USA; 66 Department of Public Health Sciences, Henry Ford Health System, Detroit, MI, USA; 67 The Population Sciences Branch, Division of Intramural Research, National Heart, Lung, and Blood Institute, Bethesda, MD, USA; 68 Ministry of Health, Government of Samoa, Apia, Samoa; 69 Department of Epidemiology & International Health Institute, School of Public Health, Brown University, Providence, RI, USA; 70 Lutia i Puava ae Mapu i Fagalele, Apia, Samoa; 71 Instituto de Medicina Tropical da Faculdade de Medicina da Universidade de São Paulo, São Paulo, Brazil; 72 Duke Comprehensive Sickle Cell Center, Duke University Medical Center, Durham, NC, USA; 73 Department of Biostatistics, University of Alabama at Birmingham, Birmingham, AL, USA; 74 Departments of Pathology & Laboratory Medicine and Biochemistry, Larrner College of Medicine, University of Vermont, Colchester, VT, USA; 75 Department of Epidemiology, Boston University School of Public Health, Boston, MA, USA; 76 Departments of Medicine, Pharmacology, and Biomedical Informatics, Vanderbilt University Medical Center, Nashville, TN, USA; 77 Division of Endocrinology and Metabolism, Department of Internal Medicine, Taichung Veterans General Hospital, Taichung, Taiwan; 78 Division of Pulmonary, Allergy, and Critical Care Medicine, University of Pittsburgh, Pittsburgh, PA, USA; 79 Institute for Translational Genomics and Population Sciences, Departments of Pediatrics and Medicine, The Lundquist Institute for Biomedical Innovation at Harbor-UCLA Medical Center, Torrance, CA, USA; 80 Department of Medicine, Vanderbilt University School of Medicine, Nashville, TN, USA; 81 Department of Medicine, Beth Israel Deaconess Medical Center, Harvard Medical School, Boston, MA, USA; 82 Division of Pulmonary and Critical Care Medicine, Brigham and Women’s Hospital, Boston, MA, USA; 83 Division of Pulmonary Medicine, Boston Children’s Hospital, Boston, MA, USA; 84 Cardiovascular Health Research Unit, Departments of Medicine, Epidemiology, and Health Services, University of Washington, Seattle, WA, USA; 85 Genes and Human Disease Research Program, Oklahoma Medical Research Foundation, Oklahoma City, OK, USA; 86 Geriatrics Research and Education Clinical Center, Baltimore Veterans Administration Medical Center, Baltimore, MD, USA; 87 Asthma & Airway Disease Research Center, University of Arizona, Tucson, AZ, USA; 88 The Mindich Child Health and Development Institute, Icahn School of Medicine at Mount Sinai, New York, NY, USA; 89 Division of Allergy and Clinical Immunology, The Ann and Robert H. Lurie Children’s Hospital of Chicago, and Department of Pediatrics, Northwestern University, Chicago, IL, USA; 90 Division of Public Health Sciences, Fred Hutchinson Cancer Research Center, Seattle, WA, USA; 91 UCSF Benioff Children’s Hospital, Oakland, CA, USA; 92 Department of Epidemiology and Population Health, Albert Einstein College of Medicine, Bronx, NY, USA; 93 Department of Medicine, Tulane University School of Medicine, New Orleans, LA, USA; 94 Mindich Child Health and Development Institute, Departments of Pediatrics and Genetics & Genomic Sciences, Icahn School of Medicine at Mount Sinai, New York, NY, USA; 95 Brown Foundation Institute of Molecular Medicine, McGovern Medical School, University of Texas Health Science Center at Houston, Houston, TX, USA; 96 Human Genetics Center, School of Public Health, University of Texas Health Science Center at Houston, Houston, TX, USA; 97 Cardiology Division, Department of Medicine, Massachusetts General Hospital, Boston, MA, USA; 98 Division of Biomedical Statistics and Informatics, Mayo Clinic, Rochester, MN, USA; 99 Jackson Heart Study and Departments of Medicine and Population Health Science, Jackson, MS, USA; 100 Human Genetics Center, Department of Epidemiology, Human Genetics, and Environmental Sciences, School of Public Health, University of Texas Health Science Center at Houston, Houston, TX, USA; 101 College of Public Health, University of Kentucky, Lexington, KY, USA; 102 Division of Cardiovascular Medicine, Brigham and Women’s Hospital, Boston, MA, USA; 103 Regeneron Pharmaceuticals, Tarrytown, NY, USA; 104 Department of Physiology and Biophysics, University of Mississippi Medical Center, Jackson, MI, USA; 105 Center for Public Health Genomics, Department of Public Health Sciences, University of Virginia, Charlottesville, VA, USA; 106 Center of Human Development and Aging, Rutgers New Jersey Medical School, Newark, NJ, USA; 107 Department of Biostatistics, University of Washington, Seattle, WA, USA; 108 Division of Endocrinology, Diabetes, and Nutrition, Department of Medicine, University of Maryland School of Medicine, Baltimore, MD, USA; 109 Program for Personalized and Genomic Medicine, University of Maryland School of Medicine, Baltimore, MD, USA; 110 Vanderbilt Genetics Institute and Division of Genetic Medicine, Vanderbilt University Medical Center, Nashville, TN, USA; 111 Department of Oncology, Johns Hopkins School of Medicine, Baltimore, MD, USA; 112 Departments of Computer Science and Genetic Medicine, Johns Hopkins University, Baltimore, MD, USA; 113 Department of Epidemiology, University of Washington, Seattle, WA, USA

**Keywords:** telomeres, telomere length genetics, trans-population genome-wide association study

## Abstract

Genetic studies on telomere length are important for understanding age-related diseases. Prior GWASs for leukocyte TL have been limited to European and Asian populations. Here, we report the first sequencing-based association study for TL across ancestrally diverse individuals (European, African, Asian, and Hispanic/Latino) from the NHLBI Trans-Omics for Precision Medicine (TOPMed) program. We used whole-genome sequencing (WGS) of whole blood for variant genotype calling and the bioinformatic estimation of telomere length in n = 109,122 individuals. We identified 59 sentinel variants (p < 5 × 10^−9^) in 36 loci associated with telomere length, including 20 newly associated loci (13 were replicated in external datasets). There was little evidence of effect size heterogeneity across populations. Fine-mapping at *OBFC1* indicated that the independent signals colocalized with cell-type-specific eQTLs for *OBFC1* (*STN1*). Using a multi-variant gene-based approach, we identified two genes newly implicated in telomere length, *DCLRE1B* (*SNM1B*) and *PARN*. In PheWAS, we demonstrated that our TL polygenic trait scores (PTSs) were associated with an increased risk of cancer-related phenotypes.

## Highlights

TOPMed sequencing-based association study identifies 36 loci for telomere length
Reliable estimation of telomere length from whole-genome sequencing data using TelSeq
Fine-mapping of sentinel variants in telomere genetic loci
Polygenic trait scores offer insight into impact of TL on disease outcomes

## Graphical abstract

**Figure:**
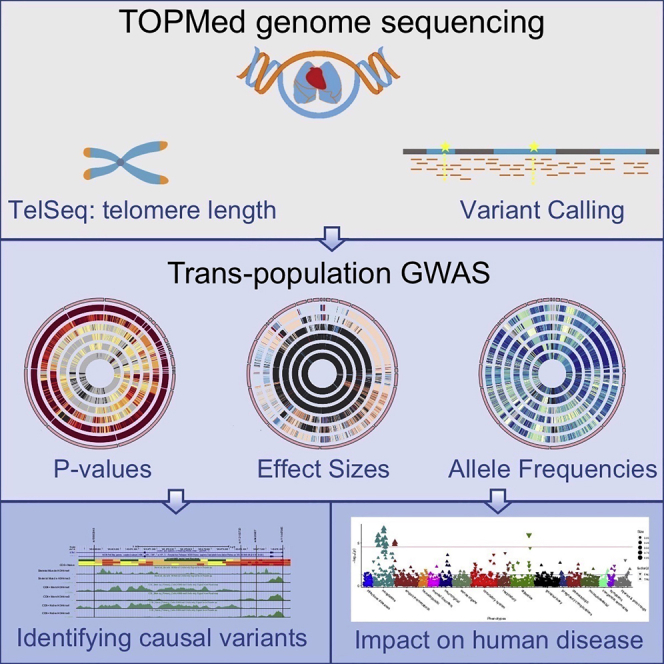

## Teaser

Taub et al. leverage TOPMed whole-genome sequencing (WGS) to estimate telomere length (TL) and report a trans-population sequencing-based association study. They identify 36 loci associated with TL and correlate polygenetic trait scores with disease outcomes.

## Introduction

Telomeres shorten in replicating somatic cells, and telomere length (TL) is associated with age-related diseases.^1^^,^^2^ To date, 17 genome-wide association studies (GWASs) have identified 25 loci for leukocyte TL,^3^, ^4^, ^5^, ^6^, ^7^, ^8^, ^9^, ^10^, ^11^, ^12^, ^13^, ^14^, ^15^, ^16^, ^17^, ^18^, ^19^ but these studies were limited to individuals of European and Asian ancestry and relied on laboratory assays of TL. The decreasing costs of high-throughput sequencing have enabled whole-genome sequencing (WGS) data generation on an unprecedented scale, including in the National Heart, Lung, and Blood Institute (NHLBI) Trans-Omics for Precision Medicine (TOPMed) cohorts. Our analyses of TOPMed data offer the opportunity to address the limitations of prior TL GWASs with increased sample size, population diversity, and inclusion of rare variant analyses and fine-mapping of the *OBFC1* locus.

In this study, we report a sequencing-based association analysis for telomere length in 109,122 ancestrally diverse individuals (European, African, Asian, and Hispanic/Latino) from the TOPMed program. We used WGS of whole blood for variant genotype calling. We used the TelSeq method for the bioinformatic estimation of telomere length from the WGS data and demonstrated that this approach has high phenotypic and genetic correlation with laboratory-based assays, providing a reliable measurement of TL. We identified 59 sentinel variants (p < 5 × 10^−9^) in 36 loci associated with TL; 20 of these are newly associated, and 13 replicated in external datasets. We also identified new common and rare variant associations at previously reported TL loci. Using WGS data also allowed fine-mapping approaches for *OBFC1*. Finally, we conducted phenome-wide association studies (PheWAS) in BioVU and identified the association of our defined polygenic trait scores (PTSs) for TL with the increased risk of cancer-related phenotypes.

## Results

We selected TelSeq^20^ to bioinformatically estimate TL due to its computational efficiency and high correlation with Southern blot^21^ and flowFISH^22^ measurements (Figures S1A–S1C; STAR Methods). We developed a principal components-based approach to remove technical artifacts arising from the sequencing process that affected TL estimation, which further improved accuracy (Figures S1D and S1E; STAR Methods). We found high phenotypic correlation of TelSeq-derived TL with TL measured by Southern blot^21^ in the 2,398 TOPMed samples from the Jackson Heart Study (JHS) and with TL measured by flowFISH^22^ in a set of 19 TOPMed GeneSTAR samples (r = 0.68 and 0.80, respectively; Figures S1C–S1E; STAR Methods). In addition, we observed high genetic correlation between TelSeq and Southern blot assays of TL in the subset of 1,083 family-based JHS samples (ρ_g_ = 0.8069, SE = 0.05, estimated using SOLAR^23^). Together, the phenotypic and genetic correlations with the more traditionally used Southern blot or flowFISH assays suggest the suitability of TelSeq-based TL as a potential TL measure for large-scale genetic epidemiologic study.

**Figure 1:**
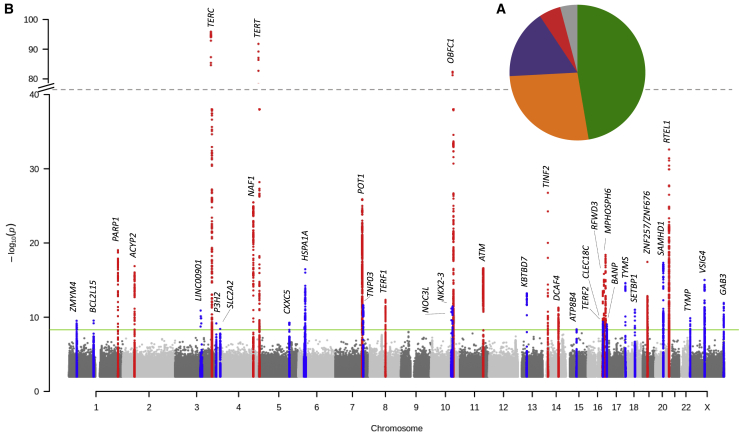
Genome-wide Manhattan plot (A) Pie chart showing population groups based on HARE for samples included in analysis: European (green, n = 51,654), African (orange, n = 29,260), Hispanic/Latino (purple, n = 18,019), Asian (red, n = 5,683), and Other/Mixed/Unknown (gray, n = 4,506). (B) Trans-population genome-wide tests for association using 163 million sequence-identified variants on n = 109,122 samples with sequence-generated telomere length from TOPMed. All loci had a peak p < 5 × 10^−9^ in the pooled trans-population analysis. Previously reported loci for TL are indicated in red, and loci newly associated in the present study are indicated in blue. Note the shift in scale above the y axis break; no peak variants had a p value within the y axis break.

Pooled trans-population association analysis was performed with n = 109,122 individuals from TOPMed (including 51,654 of European ancestry, 29,260 of African ancestry, 18,019 Hispanic/Latinos, 5,683 of Asian ancestry, and 4,506 of other, mixed, or uncertain ancestries, as determined by harmonized ancestry and race/ethnicity [HARE]^24^;Figure 1A; STAR Methods); 44% were male and ages ranged from <1 to 98 years old (Table S1). Genome-wide tests for association were performed across 163 million variants. Using a series of single-variant tests for association (primary to identify loci, iterative conditional by chromosome to identify additional independent variants, and joint tests, including all independent variants to summarize effect sizes; see STAR Methods), we identified 59 independently associated variants mapping to 36 loci, meeting the significance threshold of p < 5 × 10^−9^ (Figure 1B; Tables 1 and S2); 16 of these were previously reported and 20 were newly associated loci, as described further below.

**Table 1:** 59 variants independently associated with telomere length, mapping to 36 loci, in 109,122 ancestrally diverse (African, European, Hispanic/Latino, Asian) individuals from TOPMed

						Single variant analysis in pooled trans-population sample		p values from joint model					Effect sizes from joint model					Cochran’s Q (p value)
Chr	Locus	SNP	Prior GWAS	Annotation	MAC	p value	Percent variation explained	Trans- population	European	African	Hispanic/ Latino	Asian	Trans- population	European	African	Hispanic/ Latino	Asian	
1	ZMYM4	rs11581846		7	87500	3.04E−10	0.036%	1.74E−11	7.99E−5	8.80E−3	3.37E−6	4.13E−1	−19.9	−21.1	−15.0	−24.6	−8.7	0.44
1	BCL2L15	rs2296176		7	33274	2.84E−10	0.036%	1.52E−10	6.57E−8	2.38E−3	3.16E−1	2.07E−2	−19.5	−22.4	−22.0	−7.3	−26.9	0.28
1	PARP1	rs1136410	Known	missense	36395	9.32E−20	0.076%	9.14E−22	6.10E−9	1.02E−2	7.51E−7	2.04E−4	−29.3	−26.0	−23.7	−29.5	−32.5	0.87
2	ACYP2	rs17189743	Known	missense	3741	7.18E−12	0.043%	4.24E−11	2.70E−6	2.52E−1	5.20E−6	1.94E−1	−55.4	−50.7	−30.4	−82.4	−46.8	0.34
2		rs144980386		deletion	28773	1.32E−17	0.067%	1.99E−17	7.78E−13	7.32E−4	1.45E−4	6.78E−1	27.5	33.6	21.2	30.5	4.8	0.08
3	LINC00901	rs961617801		6	12	1.25E−11	0.042%	4.81E−11	2.82E−10	–	–	–	1009.6	1038.5	–	–	–	–
3	TERC	rs12637184	Known	4	47452	1.30E−96	0.399%	1.58E−103	1.02E−50	1.84E−15	7.32E−30	2.33E−11	−59.8	−57.0	−65.8	−66.1	−57.3	0.51
3		rs9826466		N/A	4066	3.25E−17	0.065%	3.66E−21	2.04E−01	7.28E−19	2.96E−03	–	−77.0	253.9	−77.6	−77.8	–	0.25
3	P3H2	rs10937417		7	80209	6.89E−10	0.035%	1.89E−10	9.04E−5	1.65E−5	2.33E−3	6.30E−1	14.6	13.5	17.9	17.1	−4.4	0.16
4	SLC2A2	rs4235345		6	44302	3.82E−9	0.032%	1.88E−9	3.24E−7	4.39E−2	1.86E−2	5.70E−2	16.8	19.8	12.8	13.2	47.1	0.41
4	NAF1	rs60735607∗	Known	6	57418	0.003964	0.008%	4.43E−12	4.45E−7	7.20E−3	5.33E−4	9.94E−1	−18.5	−20.0	−12.9	−22.4	−0.1	0.43
4		rs113580095		7	290	4.72E−18	0.069%	1.63E−16	2.57E−8	7.12E−2	3.40E−7	–	−254.7	−231.9	−287.8	−257.9	–	0.89
4		rs1351222		7	50104	3.27E−26	0.103%	1.60E−32	9.04E−16	1.50E−7	6.80E−11	6.58E−3	32.7	32.7	28.9	41.2	28.9	0.49
5	TERT	rs192999400	Known	5	2470	3.10E−15	0.057%	8.21E−23	2.15E−1	3.90E−17	3.42E−3	6.19E−2	101.3	88.7	97.4	96.9	108.4	1.00
5		rs6897196		5	102081	1.87E−83	0.344%	6.04E−13	7.24E−4	7.90E−8	1.87E−2	1.46E−1	20.8	16.2	25.2	16.8	23.4	0.55
5		rs7705526∗∗		7	64162	1.64E−92	0.382%	2.01E−18	1.16E−11	7.73E−4	1.46E−3	6.47E−2	30.0	35.5	22.5	27.1	34.1	0.47
5		rs2853677∗∗		5	80905	8.17E−65	0.265%	1.25E−19	9.27E−7	8.88E−10	1.33E−5	8.34E−2	−23.9	−17.7	−33.9	−28.9	−22.0	0.08
5		rs34052286		3a	6173	1.50E−12	0.046%	5.47E−22	3.86E−1	1.09E−14	2.39E−5	–	−66.0	−59.3	−61.6	−74.3	–	0.80
5		rs114616103∗		7	4527	2.39E−7	0.024%	2.03E−13	1.44E−9	2.09E−2	1.72E−2	4.93E−1	−57.2	−59.2	−37.9	−57.6	−71.9	0.73
5	CXXC5	rs75903170		2b	11895	5.69E−10	0.035%	7.01E−10	2.83E−4	2.58E−6	8.14E−2	4.31E−1	29.6	25.5	46.7	22.3	12.6	0.19
6	HSPA1A	rs1008438		2b	106908	3.42E−17	0.065%	6.64E−19	3.61E−9	1.40E−4	4.10E−7	1.71E−2	−20.3	−19.7	−18.4	−25.7	−20.5	0.73
7	POT1	rs720613	Known	7	62325	1.27E−26	0.105%	1.37E−27	2.59E−18	5.87E−6	3.63E−5	2.51E−2	−26.3	−31.3	−20.4	−24.6	−21.0	0.26
7		rs202187871		missense	27	4.89E−12	0.044%	6.72E−13	6.42E−12	–	–	–	738.3	719.6	–	–	–	–
7	TNP03	rs7783384		5	92528	2.34E−12	0.045%	6.10E−12	1.22E−7	1.47E−3	2.54E−2	2.04E−2	−15.2	−17.8	−13.3	−11.4	−19.8	0.64
8	TERF1	rs183633026	Known	7	1132	1.59E−10	0.038%	1.45E−10	3.48E−2	7.67E−1	1.51E−9	–	99.4	75.4	−22.0	109.6	–	0.18
8		rs73687065		5	1676	3.10E−12	0.045%	8.10E−12	7.74E−1	2.76E−10	5.71E−3	–	85.3	24.9	87.4	97.0	–	0.74
8		rs10112752		7	78968	4.59E−13	0.048%	9.83E−12	5.13E−9	5.69E−3	4.16E−2	2.83E−2	−15.8	−19.2	−12.6	−11.2	−26.2	0.40
10	NOC3L	rs3758526		missense	32511	6.80E−12	0.043%	5.77E−13	1.63E−5	2.70E−5	1.38E−2	1.42E−2	−22.0	−21.1	−22.6	−19.8	−23.1	0.99
10	NKX2-3	rs10883359		7	54905	3.60E−12	0.044%	9.34E−11	5.46E−5	5.14E−6	3.46E−2	1.70E−1	−16.5	−14.5	−28.1	−11.9	−11.9	0.19
10	OBFC1	rs10883948	Known	7	94489	2.04E−34	0.137%	3.97E−12	1.20E−5	3.99E−4	2.18E−2	6.42E−4	−18.8	−15.7	−24.2	−13.9	−46.8	0.11
10		rs112163720∗		4	15559	0.440005	0.001%	4.86E−16	9.57E−7	4.34E−7	3.69E−4	9.38E−4	37.1	48.1	39.1	35.3	54.9	0.66
10		rs9420907∗∗		3a	54838	3.90E−83	0.342%	6.80E−54	3.65E−18	6.94E−19	6.79E−13	9.41E−1	−49.2	−44.3	−52.4	−53.8	−2.4	0.30
10		rs111447985		2a	2391	2.29E−24	0.095%	3.03E−35	4.94E−3	2.24E−2	5.44E−22	2.81E−10	131.9	120.7	98.1	143.4	137.2	0.77
11	ATM	rs61380955	Known	7	105969	2.47E−17	0.066%	1.11E−18	5.79E−14	2.97E−4	2.47E−3	9.19E−2	−19.6	−24.8	−15.6	−15.7	−14.6	0.24
13	KBTBD7	rs1411041		6	85572	6.29E−14	0.052%	6.65E−15	1.46E−8	6.04E−4	1.13E−3	1.77E−2	22.4	25.5	20.5	20.2	20.8	0.87
14	TINF2	rs28372734	Known	4	2648	1.74E−27	0.108%	1.27E−30	4.91E−2	3.42E−6	4.59E−9	7.26E−10	112.6	120.9	103.9	132.1	94.8	0.59
14		rs8016076		2b	1977	1.80E−11	0.041%	4.46E−13	1.01E−1	1.70E−10	6.73E−3	–	83.8	374.8	80.9	87.5	–	0.43
14		rs41293824		5	1543	1.31E−9	0.034%	1.87E−10	7.43E−1	1.83E−7	2.58E−4	–	83.1	40.9	76.5	125.7	–	0.40
14	DCAF4	rs2572	Known	5	20731	5.14E−12	0.044%	6.70E−14	2.00E−7	1.07E−4	3.42E−3	1.75E−3	28.0	27.7	36.5	25.5	33.8	0.80
15	ATP8B4	rs7172615		4	41027	4.31E−9	0.032%	3.53E−10	1.14E−7	3.77E−3	1.96E−1	1.90E−1	−17.8	−20.3	−21.4	−8.7	−12.0	0.41
16	TERF2	rs9925619	Known	7	66224	3.01E−14	0.053%	7.84E−15	3.03E−4	1.01E−7	1.02E−6	5.10E−1	18.6	13.1	22.5	27.9	8.9	0.10
16	CLEC18C	rs62049363		7	61724	4.09E−10	0.036%	3.25E−11	5.24E−7	1.44E−1	4.80E−4	3.52E−1	−16.8	−16.7	−11.2	−19.4	−8.5	0.69
16	RFWD3	rs7193541	Known	missense	93079	1.47E−16	0.063%	3.18E−17	3.39E−12	5.63E−5	1.66E−3	5.21E−1	−18.7	−22.9	−16.8	−16.9	−5.6	0.24
16	MPHOSPH6	rs2967355	Known	6	34993	3.96E−19	0.073%	2.04E−20	2.69E−11	1.58E−4	4.86E−7	9.91E−1	−28.2	−26.2	−33.8	−36.8	−0.1	0.05
16	BANP	rs12934497		5	77109	9.15E−10	0.034%	8.16E−0	1.53E−5	1.62E−3	5.66E−3	1.15E−1	14.6	14.1	15.2	15.2	31.8	0.86
18	TYMS	rs150119891∗		5	1320	2.27E−7	0.025%	1.92E−11	3.69E−10	2.80E−1	2.89E−3	–	−98.8	−104.9	−52.3	−160.9	–	0.32
18		rs8088781		5	25774	2.49E−15	0.057%	8.91E−32	2.78E−15	1.75E−8	8.74E−9	8.66E−2	−50.6	−56.0	−40.8	−59.3	−160.9	0.21
18		rs2612101∗		5	56194	0.407696	0.001%	6.29E−16	7.76E−7	2.11E−4	5.13E−8	3.89E−2	26.0	29.4	18.4	35.1	171.4	0.05
18	SETBP1	rs2852770		7	46513	1.00E-11	0.042%	1.15E-12	2.32E-09	4.37E-02	1.23E-04	7.01E-01	−19.0	−25.1	−9.4	−24.3	−4.1	0.03
19	ZNF257/ZNF676	rs8105767∗∗	Known	6	76591	3.59E−18	0.069%	1.52E−18	6.32E−9	1.14E−7	5.00E−3	5.14E−3	20.3	20.8	22.2	15.0	25.5	0.68
20	SAMHD1	rs2342113		6	51830	4.62E−18	0.069%	1.58E−19	2.50E−13	4.00E−4	1.34E−6	3.67E−1	−23.7	−33.8	−16.1	−27.4	−7.7	0.01
20	RTEL1	rs41308088	Known	5	14105	1.58E−10	0.038%	8.42E−16	6.46E−15	6.24E−2	4.61E−2	2.05E−1	37.1	45.3	25.1	20.1	58.1	0.12
20		rs79981941		6	21592	1.46E−23	0.092%	1.37E−12	5.06E−6	4.73E−3	5.48E−6	9.09E−2	−26.7	−26.4	−18.6	−39.1	−47.5	0.25
20		rs41309367∗∗		5	71377	2.52E−33	0.133%	1.01E−42	5.92E−23	1.49E−15	9.58E−9	6.05E−1	−34.1	−37.0	−38.0	−33.0	−5.1	0.02
20		rs35640778		missense	2354	2.31E−28	0.112%	1.47E−38	3.77E−29	2.06E−7	1.11E−4	9.25E−1	−140.5	−141.8	−176.3	−116.7	13.8	0.42
20		rs181080831		synonymous	675	6.40E−17	0.064%	7.53E−19	8.00E−18	8.50E−1	1.39E−2	–	180.5	199.5	15.5	133.0	–	0.07
22	TYMP	rs361725		3a	101750	1.36E−10	0.038%	8.26E−12	3.97E−11	2.32E−1	4.11E−2	2.39E−2	−15.6	−22.1	−5.4	−10.6	−21.2	0.02
X	VSIG4	rs12394264		4	50912	9.67E−16	0.059%	5.36E−17	1.57E−7	1.94E−5	4.13E−5	7.28E−1	19.0	19.2	15.7	20.6	13.3	0.85
X	GAB3	rs2728723		5	63517	1.21E−12	0.046%	3.47E−12	4.45E−8	8.67E−2	7.96E−4	4.55E−2	13.2	15.7	6.0	14.4	17.0	0.17

Loci are labeled as known if the sentinel variants in the locus were in LD (r^2^ ≥ 0.7) with previously reported GWAS association for telomere length. There are 5 variants marked with an asterisk where the primary analysis did not meet our threshold of p < 5 × 10^−9^; however, they reached significance after conditioning on significant variants mapping to the chromosome (detailed in Table S2). Variants marked with a double asterisk are direct matches to prior reported sentinel variants. Percentage of trait variation explained by each variant is provided from single-variant association tests. p values and effect sizes (in base pairs) are reported from a joint model including all variants. p values for effect heterogeneity across population groups were generated using Cochran’s Q statistic. MAC is the minor allele count from the full combined sample. For all exonic variants, detailed annotation is provided, while for all non-coding variants, the RegulomeDB score is given.

See also Tables S2 and S4.

**Figure 2:**
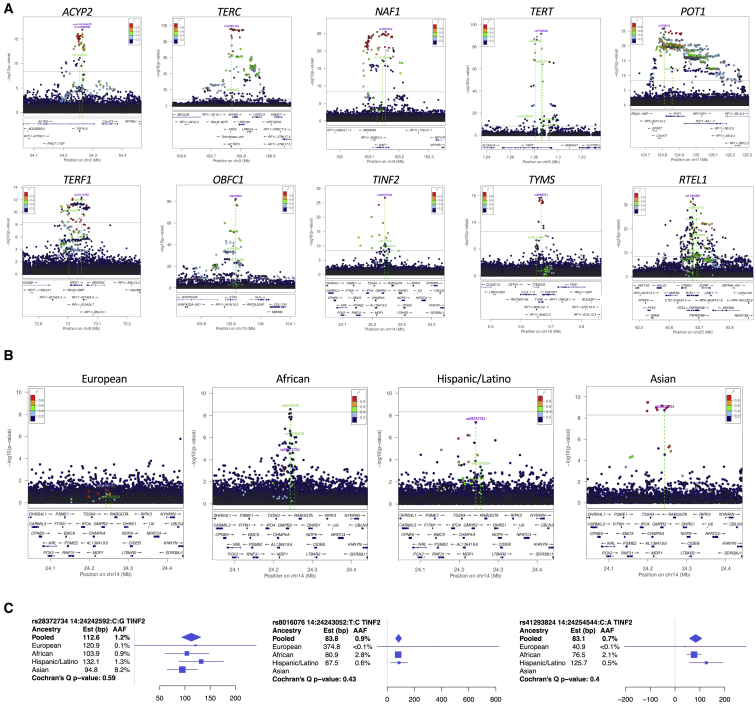
LocusZoom plots for multi-hit loci and *TINF2* (A) LocusZoom plots for all loci with >1 sentinel variant. Linkage disequilibrium (LD) was calculated from the set of samples used in the analysis with respect to the peak variant in the pooled trans-population primary analysis, thereby reflecting LD patterns specific to the TOPMed samples. For each figure, the peak sentinel variant from the pooled trans-population analysis is indexed and labeled in purple, and all of the independent variants identified through the iterative conditional approach are labeled in green and highlighted with green dotted lines. (B) LocusZoom plots for 4 population groups for the *TINF2* locus. (C) Forest plots displaying effect sizes and standard errors, as well as minor allele frequencies, by population group for the 3 sentinel variants in *TINF2*. See also Table S2.

We examined 25 previously reported loci for TL identified through GWASs, using qPCR or Southern blot assays to directly measure TL, for evidence of replication in our study. For 16 loci (*PARP1*, *ACYP2*, *TERC*, *NAF1*, *TERT*, *POT1*, *TERF1*, *OBFC1*, *ATM*, *TINF2*, *DCAF4*, *TERF2*, *RFWD3*, *MPHOSPH6*, *ZNF208/ZNF257/ZNF676*, and *RTEL1*), there was at least 1 variant with p <5 × 10^−9^ in our trans-population TL analysis that was in linkage disequilibrium (LD) (r^2^ ≥ 0.7) with a published genome-wide significant (p <5 × 10^−8^) variant from a previous study (Tables 1 and S3). Directionally consistent and nominal evidence for replication was noted for *CTC1* (rs3027234, p = 7.97 × 10^−5^) and *SENP7* (rs55749605, p = 0.023). A signal previously attributed to *PRRC2A* is located <200 kb from our signal for *HSPA1A* but may be distinct given the low LD (r^2^ = 0.26). We found no evidence of replication (all variants with p > 0.05) for the remaining previously reported TL loci (*CXCR4*, *PXK*, *MOB1B*, *DKK2/PAPSS1*, *CARMIL1*, and *CSNK2A2*; Table S3)*.* Our comprehensive conditional analyses identified ≥1 independent sentinel variants at 9 of the 16 previously reported loci (Figure 2A; Table 1). The resolution possible with our trans-population WGS data identified a sentinel variant different from the one previously reported by tagging-based GWASs for 11 of the 16 known loci. At known loci *RTEL1*, *RFWD3*, *POT1*, *ACYP2*, and *PARP1*, our WGS-based sentinels included a coding missense variant in genes *RTEL1*, *RFWD3*, *POT1*, *TSPYL6*, and *PARP1*, respectively. For the remaining known TL loci, many of the non-coding sentinel variants are annotated as having regulatory evidence (RegulomeDB score < 7; Table 1), as illustrated further for *OBFC1* below.

A total of 22 independent sentinel variants were located at the 20 newly associated loci (Table 1). We examined 19 of these sentinel variants for evidence of association in 2 previously qPCR-based TL GWASs with non-overlapping subjects^18^^,^^19^ (Figure S2A). Variants at 10 of these loci (*BCL2L15*, *CXXC5*, *HSPA1A*, *NOC3L*, *NKX2-3*, *ATP8B4*, *CLEC18C*, *TYMS*, *SAMHD1*, and *TYMP*) had a Bonferroni-corrected p < 0.05/19 = 0.0026, and an additional 3 had variants with p < 0.05 (*TNP03*, *KBTBD7*, and *BANP*), as did a second variant at *TYMS*. The variant at *SAMHD1* was previously reported at an false discovery rate (FDR) < 0.05 (p = 1.41 × 10^−7^),^19^ but here has genome-wide significance (p = 1.58 × 10^−19^). Proteins encoded by two of these genes have strong biological connections to TL: CXXC5, which physically interacts with ATM and transcriptionally regulates p53 levels^25^; two proteins implicated in telomere length regulation; and BANP (also known as SMAR1), which forms a complex with p53 and functions as a tumor suppressor.^26^

There is high consistency in the effect sizes at the 59 sentinel variants observed in our TelSeq-based TL GWAS compared to prior GWASs using qPCR assays of TL (Figure S2B). The Pearson correlation was 0.92 (p = 2.1 × 10^−15^, for 37 overlapping variants) for our study compared to Dorajoo et al.^18^ (n = 23,096 Singaporean Chinese; Figure S2B, upper panel) and was 0.86 (p = 1.2 × 10^−13^, for 43 overlapping variants) for our study compared to Li et al.^19^ (n = 78,592 European; Figure S2B, lower panel). However, qPCR is a relative measure of TL (relative to a single copy gene, see Note S1); it has different units from our TelSeq measurement, which is in base pairs. A direct comparison between effect sizes in base pairs from Southern blot and TelSeq in the 2,398 JHS samples (STAR Methods) confirms very high correlation (r = 0.92, p = 1.6 × 10^−20^ for 49 variants with minor allele frequency (MAF) ≥1% in the JHS samples; Figure S2C). We note that the effect sizes in bp for TelSeq are around half those for Southern blot (slope = 0.56, p = 1.6 × 10^−20^). This mirrors what we observe in a direct comparison of the TelSeq and Southern blot TL values (Figure S1D): a 1 bp increase in Southern blot TL corresponds to a 0.42 bp increase in TelSeq TL (p = 4.9 × 10^−319^). Furthermore, the age effects on TL in these JHS samples show a similar pattern: the estimated per year decline in TL is 22 bp (p = 1.9 × 10^−114^) for Southern blot compared to 11 bp (p = 1.8 × 10^−69^) for TelSeq (STAR Methods). As a further assessment of the general reliability of our TelSeq measurements for large-scale genetic epidemiologic analysis, we performed cross-trait LD score regression with LDSC^27^^,^^28^ using GWAS summary statistics from the European ancestry group (n = 51,564) in our TOPMed WGS-based analysis and the Li et al.^19^ analysis on n = 78,592 European ancestry individuals with qPCR-measured TL. The genetic correlation was 0.8066 (SE = 0.09, p = 1.8 × 10^−17^), indicating a high degree of shared genetic determinants of TL from the 2 different measurement technologies.

Each of the 59 sentinel variants individually accounted for a small percentage of phenotypic variation (Table 1), consistent with prior GWASs of TL but cumulatively accounted for 4.35% of TL variance, compared to 2%–3% from prior GWASs.^3^ The 37 variants mapping to 16 known loci explained 3.38% of TL variability, and an additional 0.96% was explained by the 22 variants mapping to our 20 newly associated loci, representing a sizable gain in explained variability for TL from the present study. Prior GWASs using Southern blot and qPCR report allelic effects ranging from ∼49 to 120 bp.^3^^,^^4^^,^^11^^,^^13^ In the TOPMed data, our estimated effect sizes for common variants (minor allele frequency, MAF ≥ 5%) ranged from 2 to 59 bp per allele. In comparison, the effect sizes were larger for rare and low-frequency variants (MAF < 5%) in the TOPMed data (40–1,063 bp per allele).

Stratified association analyses were performed in population groups with at least 5,000 samples to evaluate effect heterogeneity of the 59 sentinel variants (Table S4). Reduced sample sizes, coupled with variation in allele frequency, often limited our power to detect population-specific associations at GWAS thresholds in the individual strata (Table S4); no additional loci were identified. A major advantage of our analysis was the ability to rely on the individual-level WGS data for the iterative conditional approach to identify the final set of independent sentinel variants at each locus. The identified sentinel variants show little evidence for heterogeneity across populations (Table 1). All Cochran’s Q^29^ p values (Table 1) were above a Bonferroni correction threshold (p > 0.001), and the 5 with nominal significance (0.001 < p < 0.05) appear to be primarily driven by differences in the (smallest) Asian stratum. An interesting illustration of a locus with strong allele frequency differences between groups is *TINF2*; the evidence at the peak variant (rs28372734) in the trans-population analysis was driven by the smaller Hispanic/Latino and Asian groups (group-specific p 4.6 × 10^−9^ and 7.3 × 10^−10^, respectively), and the secondary peak (rs8016076) was driven by the African group (group-specific p 1.7 × 10^−10^; Figure 2B; Table 1). No association is noted in the European group, where these variants are nearly monomorphic (Figure 2C).

Gene-based tests in the combined sample of all 109,000 individuals identified 8 protein-coding genes with deleterious rare and low-frequency (MAF < 1%, including singletons) variants associated with TL (p < 1.8 × 10^−6^, see Figure S3; STAR Methods). Six of these genes were previously identified GWAS loci (*POT1*, *TERT*, *RTEL1*, *CTC1*, *SAMHD1*, and *ATM*), now adding support for rare variant associations in these genes. Both *DCLRE1B* and *PARN* have been implicated in short telomere syndrome (STS) patients.^30^, ^31^, ^32^ DCLRE1B protein localizes to the telomere via interaction with the protein of another previously implicated GWAS gene, *TERF2*, and contributes to telomere protection from DNA repair pathways.^33^^,^^34^ Notably, two *PARN* loss-of-function variants included in our gene-based test were previously identified in STS patients.^30^ Both rs878853260 and rs876661305 produce frameshift mutations; rs876661305 produces an early termination codon, truncating most of the nuclease domain.^35^ For each of these 8 genes, a leave-one-out approach iterating over each variant included in the aggregate test showed there were no detectable main driver variants and indicated that these gene-based association signals arise from cumulative signals across multiple rare deleterious variants (Figure S3), with the possible exception of *ATM*. When conditioned on the 59 sentinel variants, all of the genes, except *POT1*, maintained or increased statistical significance (Figure S3). For *POT1*, while the removal of the single variant identified in Table 1 (rs202187871) and conditioning on all 59 sentinels resulted in a decrease in significance from 1.52 × 10^−24^ to 5.53 × 10^−18^, it nonetheless remained strongly significant, meeting Bonferroni thresholds.

**Figure 3:**
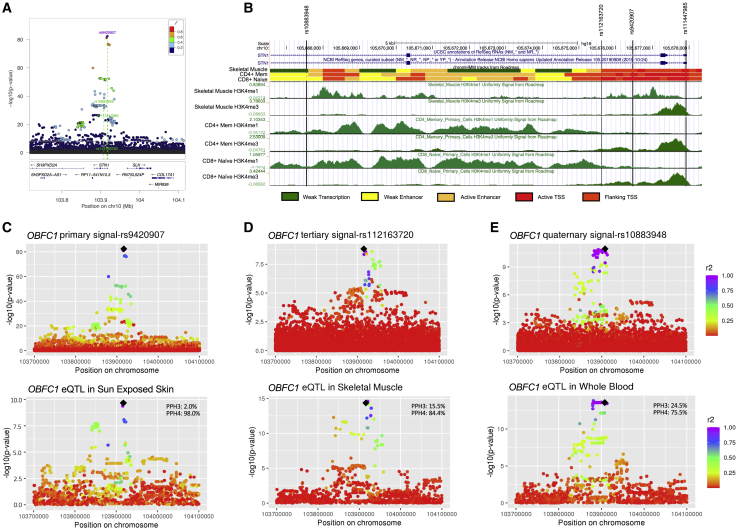
Fine-mapping of multiple *OBFC1* signals (A) LocusZoom plot of the *OBFC1* locus, where green dotted lines indicate each independent signal, as in Figure 2. (B) Roadmap Epigenomics Consortium data in hg19 coordinates for skeletal muscle tissue, Primary T CD4^+^ memory cells from peripheral blood, and primary T CD8^+^ naive cells from peripheral blood (Roadmap samples E108, E037, and E047, respectively; data were not available for sun-exposed skin). The ChromHMM state model is shown for the 18-state auxiliary model. The state model suggests the primary (rs9420907), secondary (rs111447985), and tertiary (rs112163720) signals are in the promoter region, while the quaternary signal (rs10883948) is in an enhancer region in all Roadmap blood cell types but is transcriptional for peripheral blood monocytes and CD19^+^ B cells. (C–E) GWAS and eQTL results for the primary (C), tertiary (D), and quaternary (E) signals. The top panels are the GWAS summary statistics from the primary, and iterative conditional analyses that were used to perform colocalization analysis (secondary signal was rare and not available for colocalization). Bottom panels are eQTLs for *OBFC1* in the indicated tissue from GTEx. The GTEx eQTLs for these tissues do not colocalize with one another (PPH4 < 4.4 × 10^−7^), and each signal did not significantly colocalize in the other tissues. LD was calculated from the pooled trans-population samples with respect to the sentinel (black diamond). See also Figures S4 and S5 and Table S5.

The identification of multiple independent sentinel variants for several loci offers the unique opportunity to evaluate the potential for distinct regulatory mechanisms (Figures 2A and S4). OBFC1 is part of a complex that binds single-stranded telomeric DNA^36^ and is expressed across multiple tissues in GTEx^37^ and in whole-blood studies meta-analyzed in eQTLGen.^38^ All four signals at the OBFC1 locus are in the promoter and early introns of OBFC1 (Figure 3A and 3B). Evidence for expression quantitative trait loci (eQTL) colocalization was detected at the primary, tertiary, and quaternary signals in various tissues (STAR Methods). While all 3 signals colocalized with OBFC1 eQTLs, the strongest colocalization evidence in each case was in a distinct tissue: sun-exposed skin from the lower leg (posterior probability of shared signal, PPH4 = 98.0%) for the primary, skeletal muscle (PPH4 = 84.4%) for the tertiary, and whole blood (GTEx PPH4 = 75.5%, eQTLGen PPH4 = 75.5%) for the quaternary signal (Figures 3C–3E and S5E; Table S5). Data from the Roadmap Epigenomics Consortium^39^ indicate that all 4 signals are consistent with promoter or enhancer regions across blood cells and skeletal muscle tissue (Figure 3B). We were unable to perform colocalization analysis on the secondary signal with data from either GTEx or eQTLGen as it is driven by rare variants only in the Hispanic/Latino and Asian individuals (rs111447985; Table S4).

Using individual-level data within the Vanderbilt University biobank BioVU, we performed a PheWAS (Table S6) using 49 available sentinel variants individually in addition to a TL polygenic trait score (PTS). The PTS was generated separately for European and African individuals in BioVU as a simple linear combination of the effect sizes from the stratified joint analysis in European or African individuals, respectively (Table 1, Effect sizes from joint model). PTS values were significantly higher in BioVU African Americans (AAs, mean = −217 bp, SD = 96 bp) compared to European Americans (EAs, mean = −279 bp, SD = 96 bp, p < 0.05, Welch’s 2-sample t test; Figure S6A), offering evidence that previously observed differences in TL by ancestry (longer TL in individuals of African ancestry^1^) may be explained in part by the genetic contribution to TL. The largest cumulative effect of the sentinel variants, as evidenced from the PTS, is for the category of neoplasms in the EAs, with higher TL PTS associated with increased risk to the individual cancer phenotypes (11 of the 14 significant results after Bonferroni correction for 1,704 tested phecodes were cancer related; Figure S6B; Table S6); associations were only nominal in the BioVU AAs, likely due to lower power from the smaller sample size. Single variant PheWAS (Table S6) in the BioVU EAs are largely replicated within the UK Biobank (UKBB; Table S7), again showing strong associations with neoplasms, and in general, demonstrating the alleles that increased TL also increased risk for these cancer-related phenotypes. In addition, analyses of both the UKBB and BioVU data identified an association between the *HSPA1A* locus (rs1008438) and type 1 diabetes-related endocrine/metabolism phenotypes (BioVU p 1.2 × 10^−8^ to 4.1 × 10^−28^, UKBB p 6.7 × 10^−6^ to 2.6 × 10^−27^ for a range of phecodes grouped under 250.1); here, the allele decreasing TL increased risk for these phenotypes (BioVU odds ratios 1.4–2.1, UKBB odds ratios 1.4–2.2). This agrees with prior associations between shorter TL and increased risk of type 1 diabetes,^40^ and between the protein product of *HSPA1A* (Hsp72) and diabetic ketoacidosis.^41^

## Discussion

Leveraging WGS available through the NHLBI TOPMed program, we have illustrated the value of a large, trans-population WGS study for a harmonized phenotype of broad interest, bioinformatically estimated TL, to identify new loci associated with TL. The well-powered study enabled identification of rare deleterious variants with estimated effect sizes larger than those of common variants. Using WGS allowed us the unique opportunity to hone in on causal variants using fine-mapping approaches for one locus, *OBFC1*, and begin to characterize tissue-specific genetic effects for this locus. We were also able to establish that for most population groups, effects are highly consistent at sentinel variants, despite differences in association strength at loci such as *TINF2* and *OBFC1*, in which allele frequencies varied among populations.

One of the main limitations to the interpretation of human genetic studies of TL pertains to the heterogeneity and lack of standardization of various TL assays, and therefore comparability of results (including the genetic effect sizes) between studies^42^^,^^43^ (Note S1). To date, there is a paucity of data directly comparing WGS TL estimates with laboratory-based TL measurements in large-scale genetic epidemiologic studies.^44^ In the present study, we have performed an in-depth analysis of the robustness of our TelSeq-derived TL and the resulting GWAS statistics and report the following: (1) we confirm previous observations that TelSeq estimates are consistently shorter than Southern blot (mTRF), but that the 2 values are highly correlated^44^; (2) we demonstrate a high degree of shared heritability (i.e., genetic correlation) between TelSeq-derived and Southern blot-derived TL using phenotype-on-phenotype measures of heritability in the same subjects; (3) we see similarly high genetic correlation using GWAS summary statistic measures between qPCR- and TelSeq-derived TL GWASs in Europeans; (4) we show high correlation of effect sizes at sentinel variants between TelSeq- and qPCR-derived GWASs; and (5) we show that effect sizes from TelSeq were consistently ∼50% lower compared to Southern blot at the same variants measured on the same subjects mirroring the correlation patterns noted in the original phenotypes themselves.

### Limitations of the study

The limitations of this study include the lack of datasets that include TL measurements in diverse populations for replication of the newly associated loci and genes; external studies are limited to largely European and Asian ancestry. For example, the identified effects at the *ZMYM4* and *P3H2* loci may be larger in Hispanic/Latino and African ancestry populations, respectively, than European. In both, the strength of the association, the effect sizes, and percent variation explained in the context of relative sample size in our data are larger in these non-European groups. Our lack of replication described here may be overcome with an ability to evaluate these loci in additional studies with greater population representation.

In addition, in this study, we evaluate statistical significance for association using p values that could result in anunequal ability to define significance thresholds across allele frequencies (lower allele frequencies need higher effect sizes, for example). Alternative approaches that consider effect sizes as a prioritization scheme could be applied in the future.

Finally, our fine-mapping approach based on tissue expression is limited for many of the associated loci, due to their lack of expression in GTEx tissues. Here, we followed up on the *OBFC1* locus because the gene of interest is expressed in multiple adult tissues readily available in eQTL resources such as GTEx. In contrast, while *TERT* and *TERC* are important components of telomerase, they have low to undetectable expression in most GTEx tissue samples. As discussed by others, to adequately fine-map these loci, data on stem cell and/or developmental tissues will be important.^45^

While our TelSeq-based TL measurements and the resulting genetic effect sizes appear to be robust based on our comparison to laboratory-based assays (summarized above), caveats described (Note S1) necessitate attention when interpreting and comparing the results between large-scale TL genetic studies, especially from the perspective of clinical risk quantification. Nonetheless, the ability to implement sequence-based TL phenotype estimation in a large, trans-population WGS dataset creates opportunities to meaningfully expand our ability to evaluate the role of genes influencing TL in human health and disease, to dissect the genetic basis to TL differences across populations, and to set in place a model to leverage preexisting resources of WGS to bioinformatically quantify TL.

## STAR★Methods

### Key resources table

**Table:** 

REAGENT or RESOURCE	SOURCE	IDENTIFIER
**Deposited data**		

TOPMed genetic variant calls from whole-genome sequencing data, by study	Taliun et al., 2021^46^	See Table S8 for dbGaP study phs IDs
TOPMed phenotype data, by study	Taliun et al., 2021^46^	See Table S8 for dbGaP study phs IDs
TOPMed batch-adjusted telomere length calls, by study	This paper	See Table S8 for dbGaP study phs IDs
TOPMed GWAS summary statistics for trans-population and population subgroup analyses	This paper	dbGaP: phs001974.v3.p1

**Software and algorithms**		

All original computer code	This paper	Zenodo: https://doi.org/10.5281/zenodo.5360775
TelSeq	Ding et al., 2014^20^	https://github.com/zd1/telseq
HARE	Fang et al., 2019^24^	https://github.com/tanglab/HARE
GENESIS (R package)	Gogarten et al., 2019^47^	https://bioconductor.org/packages/release/bioc/html/GENESIS.html 10.18129/B9.bioc.GENESIS
CAVIAR	Hormozdiari et al., 2014^48^	http://genetics.cs.ucla.edu/caviar/download.html
coloc	Giambartolomei et al., 2014^49^	https://chr1swallace.github.io/coloc/articles/a01_intro.html
PheWAS (R package)	Carroll et al., 2014^50^	https://github.com/PheWAS/PheWAS

### Resource availability

#### Lead contact

Further information and requests for resources and reagents should be directed to and will be fulfilled by the lead contact, Rasika Mathias (rmathias@jhmi.edu).

#### Materials availability

This study did not generate new unique reagents.

### Experimental model and subject details

#### TOPMed study populations

Our study involves human subjects only. To perform this genome-wide association study of telomere length, we leveraged the whole genome sequence samples available through the NHLBI Trans Omics for Precision Medicine (TOPMed) program. The program currently consists of more than 80 participating studies,^51^ with a range of study designs as described in Taliun et al.^46^ Participants are mainly U.S. residents with diverse ancestries (self-reported European, African, Hispanic/Latino, Asian, and Other). Smaller representation comes from non-US populations including Samoan, Brazilian, and Asian studies. Details on the specific samples included for telomere length analysis are outlined below, details on the population groupings using HARE (harmonized ancestry and race/ethnicity) are described in detail below, and final categories summarized in Table S1; additional information is also described by TOPMed.^51^ Counts of subjects by sex are also included in Table S1. While sex is included as a covariate in all relevant models in our analysis, we do not specifically investigate the effect of sex on telomere length in this work.

#### TOPMed whole-genome sequencing (WGS)

WGS was performed to an average depth of 38X using DNA isolated from blood, PCR-free library construction, and Illumina HiSeq X technology. Details for variant calling and quality control are described in Taliun et al.^46^ Briefly, variant discovery and genotype calling was performed jointly, across all the available TOPMed Freeze 8 studies, using the GotCloud^52^ pipeline resulting in a single multi-study genotype call set.

### Method details

#### Estimating telomere length for WGS samples

A variety of computational tools exist that leverage WGS data to generate an estimate of telomere length.^44^ Here, we performed a thorough comparison of two leading methods for estimating telomere length from WGS data to choose the preferred scalable method for performing the estimation on all available samples from TOPMed. The first method, TelSeq,^20^ calculates an estimate of individual telomere length using counts of sequencing reads containing a fixed number of repeats of the telomeric nucleotide motif TTAGGG. Given that 98% of our data was sequenced using read lengths of 151 or 152 (as confirmed from the SEQ field in the analyzed CRAM files), we chose to use a repeat number of 12. These read counts are then normalized according to the number of reads in the individual WGS dataset with between 48% and 52% GC content to adjust for potential technical artifacts related to GC content. The second method, Computel^53^ uses an alignment-based method to realign all sequenced reads from an individual to a “telomeric reference sequence.” Reads aligning to this reference sequence are considered to be telomeric and are included in the estimate of telomere length. Because Computel performs a complete realignment, additional computational steps are involved compared to those needed for TelSeq.

To compare the results and scalability from these two methods, we first directly compared estimates obtained from TelSeq and Computel on 2,398 samples from the Jackson Heart Study (JHS) and found them to be highly correlated with one another (Pearson correlation r = 0.98, Figure S1A). We also compared computational time to generate the telomere length estimates on these samples and show that Computel is around ten times more time-consuming (Figure S1B). This is in part due to the fact that Computel requires CRAM-formatted files (as the WGS data are currently stored) to first be converted back to Fastq format (while TelSeq requires a CRAM to BAM conversion), but also due to the computationally expensive step of realignment to the telomeric reference genome that the Computel algorithm employs.

TelSeq generates an estimate of TL in bp similar to laboratory assays such as Southern blot^21^ and flowFISH;^22^ in contrast qPCR approaches are represented as T/S ratios.^54^^,^^55^ As a further comparison to orthogonally measured telomere length values, we used data on the same 2,398 samples from JHS with Southern blot^21^ telomere length estimates.^56^ For these samples, the Southern blot assay was performed on the same source DNA sample that was used to generate the WGS in TOPMed. The Pearson correlation values between the TelSeq and Computel estimates and the Southern blot estimates did not differ (r = 0.58 and 0.56 for TelSeq and Computel, respectively, Figure S1C). Based on our observation that both Computel and TelSeq showed similar correlation to the Southern blot estimates and high correlation with each other, and that TelSeq was an order of magnitude more computationally efficient, we chose to use TelSeq to perform telomere length estimation on our data. Final telomere length estimation was performed on a set of 128,901 samples whose CRAM-files were available for analysis at the TOPMed IRC at the time of analysis.

#### Batch adjustment to correct for confounders

To account for technical sources of variability in our telomere length estimates, both within a study (see, for example, colors in Figures S1A and S1C which indicate grouping by shared 96-well plate for shipment to the sequencing center) and across studies, we developed a method to estimate components of technical variability in our samples. We estimated these covariates using the sequencing data itself, similar to methods developed for other multivariate genomics data types (SVA or PEER factors^57^^,^^58^), using aligned sequencing reads and relying on the fact that genomic coverage patterns of aligned reads can reflect technical variation.

We computed average sequencing depth for every 1,000 bp genomic region (“bin”) genome-wide using mosdepth.^59^ We removed bins known to be problematic: those containing repetitive DNA sequence with difficulty mapping (mappability < 1.0 using 50bp k-mers in GEMTools v1.759^60^) or that overlap the list of known problematic SVs^61^ or overlap known CNVs in the Database of Genomic Variants. To avoid overcorrecting for sex, bins were limited to autosomes. After normalizing the approximately 150,000 remaining bin counts within sample, we performed Randomized Singular Value Decomposition^62^ (rSVD), a scalable alternative to principal components analysis, to generate batch principal components (bPCs). We included increasing numbers of bPCs in a linear regression model predicting TelSeq TL, and computed the correlation of the resulting residuals with external data measurements, including Southern blot measurements for JHS (n = 2,398) and the Women’s Health Initiative (WHI; n = 596) and age at blood draw (JHS n = 3,294; WHI n = 10,708). Based on the observed correlation, the final decision was to include the first 200 bPCs across all samples. Using the n = 2,398 JHS samples described above, we compared TL estimates before and after batch correction. The percent of variance in TL explained by sequencing plate reduced from 21.9% (baseline) to 10.5% (200 bPCs), and the variance explained by age increased from 8.0% (baseline) to 10.3% (200 bPCs), evidence that the signal-to-noise ratio was improved. Overall, the correlation between the bPC corrected TL and Southern blot data improved from r = 0.58 to 0.68 (Figure S1D) in the JHS data and from r = 0.54 to 0.72 for the WHI data. Further, we compared TelSeq estimates of 19 samples within a single sequencing batch from the GeneSTAR study to the clinical gold standard of flowFISH^22^ (Figure S1E) and observed a correlation of 0.80 in both granulocytes and lymphocytes. Therefore, our data show that we are able to reduce the sequencing artifacts stemming from batch variability to attain correlation of TelSeq to Southern blot similar to the correlation of TelSeq to flowFISH.

#### Samples included in genetic analysis

All samples with telomere length estimated from the WGS data from TOPMed Freeze 8 were considered for inclusion, provided they had consent that allowed for genetic analysis of telomere length. Only samples with sequencing read lengths of 151 or 152 base pairs and having age at blood draw data available were included. For the set of samples that were part of a duplicate pair/group (either part of the intended duplicates designed by TOPMed, or a duplicate identified across the studies through sample QC) only one sample from each duplicated pair/group was retained. The final counts and demographic summary statistics for subjects grouped by TOPMed study for all 54 studies included in our analysis are shown in Table S1.

While self-reported race (Asian, Black and White) and Hispanic ethnicity group (Central American, Costa Rican, Cuban, Dominican, Mexican, Puerto Rican, South American) data are available in TOPMed, these data have limitations for analysis that include individuals with missing information or non-specific responses (e.g., ‘other’ or ‘multiple’) and high variability in genetically inferred measures of ancestry among individuals with the same reported race/ethnicity. To overcome these limitations, we used a computational method called HARE (harmonized ancestry and race/ethnicity), a newly developed machine learning approach for jointly leveraging reported and genetic data in the definition of population strata for GWAS.^24^ HARE uses provided race/ethnicity labels and genetic ancestry principal component (PC) values to compute probability estimates for each individual’s membership in each race/ethnicity stratum. For our HARE analysis, we used provided race (Asian, Black, White) or Hispanic ethnicity group (Central American, Costa Rican, Cuban, Dominican, Mexican, Puerto Rican, South American) as input labels to define population strata, and we used 11 PCs computed with PC-AiR^63^ using 638,486 LD-pruned (r^2^ < 0.1) autosomal variants with minor allele frequency > 1% to represent genetic ancestry. Genetic outliers for population strata were identified as individuals for whom their maximum stratum probability was more than 5 times greater than their reported stratum probability. Stratum values for genetic outliers and individuals with missing or non-specific race/ethnicity were imputed as the stratum for which they had the highest membership probability.

Our primary analysis allowed for heterogeneous residual variance (see Primary single variant tests for association for details) among groups defined jointly by study and HARE-based population stratum assignment, with minor study-specific modifications to account for small strata. We required at least 30 individuals within a study-HARE grouping and collapsed individuals into merged HARE groups within a study as necessary to retain everyone for analysis. For our population-specific analyses, we used HARE assignment to stratify individuals into the following population groups: African (corresponding to the Black HARE stratum), Asian (Asian), European (White), and Hispanic/Latino (Central American, Costa Rican, Cuban, Dominican, Mexican, Puerto Rican, and South American). To better preserve genetic ancestry similarity among individuals in population-specific stratified analyses, we restricted to individuals for whom their HARE population stratum membership probability was at least 0.7; the population stratum counts in Table S1 reflect the counts in the stratified analyses, where individuals not meeting this criterion are labeled as “Other/Uncertain.”

Samoan individuals from the Samoan Adiposity Study and Brazilian individuals from the Reds-III Brazil study were excluded from the HARE analyses due to their unique ancestry in the TOPMed dataset; these studies were treated as their own population groups for analyses.

### Quantification and statistical analysis

#### Primary single variant tests for association

Genome-wide tests for association were performed using the R Bioconductor package GENESIS.^47^ The primary analysis included all available trans-population TOPMed samples (n = 109,122). A secondary analysis was performed for all population groups with n > 5,000, which included European (n = 51,654), African (n = 29,260), Hispanic/Latino (n = 18,019) and Asian (n = 5,683) groups as defined above using HARE. Prior to genetic modeling, we generated residuals from a linear regression model on all 109,122 samples with 200 batch principal components (bPCs), as described above; for clarity we call these residuals $TL_{bPC}$ below. For the pooled trans-population analysis, we used a fully adjusted two-stage model, as described in the next two bullets.^64^ For each population-specific analysis, the same approach was used, limited to samples within that population group.

**Stage 1:** We fit a linear mixed model (LMM) on n = 109,122 samples, using $TL_{bPC}$ as the outcome; adjusting for age, sex, study, sequencing center, and 11 PC-AiR^63^ PCs of ancestry as fixed effect covariates; including a random effect with covariance matrix proportional to a sparse empirical kinship matrix computed with PC-Relate^65^ to account for genetic relatedness among samples; and allowing for heteroskedasticity of residual variance across study-HARE groups as defined above. The marginal residuals from this Stage 1 model were then inverse-normalized and rescaled by their original standard deviation. This rescaling restores values to the original trait scale, providing more meaningful effect size estimates from subsequent association tests.^66^
**Stage 2:** We fit a second LMM on all n = 109,122 samples, using the inverse-normalized and rescaled residuals from Stage 1 as the outcome; all other aspects of the model including fixed effects adjustment, random effects, and residual variance structure were identical to the model in Stage 1. This two-stage covariate adjustment has been shown to be most effective at controlling for false-positives and increasing statistical power in this setting.^64^ The output of this Stage 2 model was then used to perform both single variant and gene-based tests for association.

#### Single variant tests for association

We used the output of the two-stage LMM to perform score tests of association for each variant with minor allele count (MAC) ≥ 5 that passed TOPMed Informatics Research Center (IRC) at the University of Michigan quality filters^46^ and which had < 10% of samples with read depth < 10. Genotype effect size estimates and percent of variability explained (PVE) were approximated from the score test results.^67^

#### Significance, conditional analysis, locus definitions

A p value cutoff of 5x10^−9^ was used to determine genome-wide significance in the primary trans-ethnic analysis. We identified our set of independent significant variants (as reported in Table 1) through an iterative conditioning process within each chromosome. For a given chromosome, if at least one variant from the primary analysis crossed the genome-wide significance cutoff, this peak variant was included as an additional fixed-effect covariate in a new two-stage LMM (see Stages 1 and 2 described above), and score test results were examined to see if any remaining variants crossed the 5x10^−9^ threshold. If so, we performed a second round of conditioning, including both the original peak variant and the new conditional peak variant as fixed-effect covariates in another two-stage LMM; and so on, adding conditional peak variants for additional rounds (Table S2). For each chromosome, the conditioning procedure was completed when no additional variants crossed the genome-wide threshold (p < 5x10^−9^) on that chromosome. At each step, all variants passing the p < 5x10^−9^ threshold were examined in BRAVO^68^ to assess quality, and 334 variants were filtered out due to variant call quality issues. In the case where a current peak variant was flagged for quality, the next most significant variant, provided its p value was below the 5x10^−9^ cutoff, was considered the peak variant instead. Variants were grouped into loci based on physical distance and an examination of linkage disequilibrium (LD) patterns, and locus names were determined using a combination of previous literature, known telomere biology, and physical location.

#### Cumulative percent of variability explained (PVE)

Through the iterative conditional approach, we identified a total of 59 variants (Table 1) that met our genome-wide significance threshold of p < 5x10^−9^. The cumulative PVE values for this full set of 59 variants (4.35%), the set of 37 variants mapping to known loci (3.38%), and the set of 22 variants mapping to previously un-identified loci (0.96%, see Overlap with prior published GWAS below for definition of previously un-identified variants) were each estimated jointly using approximations from multi-parameter score tests. This joint PVE approximation is similar to the single variant PVE approximation described above, except that the set of variants is tested jointly, accounting for covariance among the estimated variant effect sizes. This approach avoids inadvertently double counting any partially shared signal among the set of identified variants.

#### Joint tests for association, cross-population heterogeneity

We then performed joint association analyses for the full multi-ethnic sample (n = 109,122), as well as each of the four population groups with n > 5000, to determine effect sizes and p values when all 59 variants were considered together. Using the inverse-normalized and rescaled residuals from the primary analysis Stage 1 LMM as the outcome, we fit a new Stage 2 LMM that was the same as described above, except with the additional inclusion of the genotypes for these 59 variants as additive genetic fixed effects. Given this joint modeling framework, the variant effect size estimates are all adjusted for one another. These estimates were used as input for calculation of a polygenic trait score used for the PheWAS described below. Finally, we tested for heterogeneity of effect sizes from these analyses among the population groups by adapting Cochran’s Q statistic and its p value,^69^ commonly used to test for effect heterogeneity in meta-analysis (Table 1). For each variant, the effect size estimates and standard errors from each population group analysis were used to calculate Q, and a Bonferroni threshold of 0.001 (0.05/59) was used to assess significance.

#### Overlap with prior published GWAS

For each of the 59 variants identified, we examined the linkage disequilibrium (LD) with previously reported sentinel variants from 17 published GWAS. Only sentinel variants with p < 5x10^−8^ in their published study were considered, which included a total of 56 variants (Table S3). If one of our variants had LD ≥ 0.7 with a published variant, it was labeled as a known variant/part of a known locus in Table 1. Within a locus, we then compared each independent variant to the prior GWAS reported sentinel variant. If they were identical, the variant was labeled as a known sentinel variant in Table 1. Additionally, locus names for the final set of independent variants were selected based on (i) prior GWAS study definition for known loci, and (ii) the specific gene annotation for each variant mapping directly to a gene for previously un-identified loci.

#### Replication of newly associated loci

To determine whether the loci newly associated in the current study are supported by findings from prior studies, we considered the two largest most recent studies of telomere genetics in European^19^ (Li et al., n = 78,592) and Asian^18^ (Dorajoo et al., n = 26,875) ancestry individuals. These studies both used telomere length as measured by qPCR. For all newly associated variants in Table 1, we pulled the effect size estimates, standard errors, and p values, where available (Figure S2A). These results were available in at least one of the two studies for 19 of our 22 previously un-identified variants, so we considered a p value cutoff of 0.05/19 = 0.0026 to be replicated, after multiple testing correction. We also labeled variants where at least one study reported p < 0.05 as suggestive. We compared effect sizes between the qPCR results and our TelSeq results, assessing correlation of all overlapping variants (n = 37 for Dorajoo et al.,^18^ n = 43 for Li et al.,^19^ Figure S2B).

#### Comparison of Southern blot and TelSeq effect sizes

Using the 2,398 samples from JHS with both TelSeq and Southern blot TL measurements, we used the same fully-adjusted two-stage LMM framework to perform tests for genetic association at the 49 of 59 sentinel variants with MAF ≥ 1% in this group. We calculated Pearson correlation between the estimated effect sizes and fit a linear regression to relate them to one another as an estimate of difference in effect size magnitude (Figure S2C). The Stage 1 models from our LMM framework also provided estimated effect sizes for the average change in Southern blot and TelSeq TL estimates in basepairs for a one-year difference in age at blood draw.

#### Genetic correlation of TelSeq with other TL estimates

We assessed genetic correlation of our TelSeq estimates with other TL estimates in two ways: (1) Using a subset of 1,083 of our 2,398 JHS samples with both Southern blot and TelSeq TL estimates who were either participants in the nested family cohort portion of JHS or unselected 1st or 2nd degree relatives from the remaining samples,^70^ we measured genetic correlation ($\rho_{G}$) of the Southern blot and TelSeq TL estimates using SOLAR^23^; and (2) We performed cross-trait LD Score regression using LDSC^27^^,^^28^ [ to estimate genetic correlation using genetic association summary statistics from our European ancestry group (n = 51,564) and summary statistics from the Li et al. study,^19^ which used qPCR to measure TL on 78,592 individuals of European ancestry. We used pre-computed LD scores from 1000 Genomes European data (downloaded from https://data.broadinstitute.org/alkesgroup/LDSCORE/eur_w_ld_chr.tar.bz2), and used the SNP list from https://data.broadinstitute.org/alkesgroup/LDSCORE/w_hm3.snplist.bz2 to match up alleles across studies and LD scores. After preprocessing and SNP checks (using defaults from LDSC), we were left with a set of 1,171,171 SNPs for the LD Score regression analysis.

#### Gene-based coding variant tests - variant annotation

For its use in gene-based tests for association, annotation based variant filtering and GENCODE v29 gene model-based^71^ aggregation was performed using the TOPMed freeze 8 WGSA Google BigQuery-based variant annotation database on the BioData Catalyst powered by Seven Bridges platform (http://doi.org/10.5281/zenodo.3822858). The annotation database was built using variant annotations for TOPMed freeze 8 variants gathered by Whole Genome Sequence Annotator (WGSA) version v0.8^72^ and formatted by WGSAParsr version 6.3.8 (https://github.com/UW-GAC/wgsaparsr). Variants were annotated as exonic, splicing, transcript ablation/amplification, ncRNA, UTR5, UTR3, intronic, upstream, downstream, or intergenic using Ensembl Variant effect predictor (VEP).^73^ Exonic variants were further annotated as frameshift insertion, frameshift deletion, frameshift block substitution, stop-gain, stop-loss, start-loss, non-frameshift insertion, non-frameshift deletion, non-frameshift block substitution, nonsynonymous variant, synonymous variant, or unknown. Additional scores used included REVEL,^74^ MCAP^75^ or CADD^76^ effect prediction algorithms.

#### Gene-based coding variant tests - tests for association

Gene-based association testing was performed on the pooled trans-population dataset (n = 109,122). To improve the power to identify rare variant associations in coding regions, we aggregated deleterious rare coding variants in all protein-coding genes and then tested for association with telomere length. To enrich for likely functional variants, only variants with a “deleterious” consequence for its corresponding gene or genes,^77^ were included. For each protein-coding gene, a set of rare coding variants (MAF < 0.01, including singletons where MAC = 1, restricted to variants which passed IRC quality filters^46^ and which had < 10% of samples with read depth < 10) was constructed, which was composed of all stop-gain, stop-loss, start-loss, transcript ablation, transcript amplification, splice acceptor variants, splice donor variants and frameshift variants, as well as the exonic missense variants that fulfilled one of these criteria: 1) REVEL score > 0.5, 2) predicted M_CAP value was “Damaging,” or 3) CADD PHRED-scaled score > 30. We applied the variant Set Mixed Model Association Test (SMMAT)^78^ as implemented in GENESIS, using the genesis_tests app on the Analysis Commons,^79^ with MAF based variant weights given by a beta-distribution with parameters of 1 and 25, as proposed by Wu et al.,^80^ and using the same two-stage LMM output as used in the primary single variant analysis. Only genes with a cumulative MAC ≥ 5 over all variants were evaluated, leaving a total of 27,558 genes, and significance was evaluated after a Bonferroni correction for multiple testing (p < 0.05 / 27,558 = 1.815x10^−6^) (Figure S3).

Next, we sought to determine the influence of each rare deleterious variant in each significant gene on the association signal. We iterated through the variants, removing one variant at a time (leave-one-out approach),^81^ and repeated the SMMAT analysis. If a variant made a large contribution to the original association signal, one would expect the signal to be significantly weakened with the removal of the variant from the set (Figure S3).

Finally, we further tested for independence of the gene-based and single variant signals by performing a conditional SMMAT analysis that included the 59 genome-wide significant variants from our primary analysis as fixed-effect covariates in the two-stage LMM. These 59 variants were also removed from the aggregated set of rare variants for a gene if they had been previously included (e.g., rs202187871 in *POT1*). All other analysis parameters were the same as described above (Figure S3).

#### Colocalization of OBFC1 signals using GTEx and eQTLGen

Iterative conditional analysis was repeated for chromosome 10 focusing on a 2Mb window centered on the primary signal near *OBFC1* (rs10883948). The original pooled GWAS results (n = 109,122) were used for colocalization analysis with the primary signal while the appropriate round of conditional analysis was used for each subsequent signal (e.g., the output of the second round of conditional analysis was used for colocalization analysis with the tertiary signal). Credible set analysis was performed using CAVIAR on primary signal data and the output of each conditional analysis each with a single assumed causal variant.^48^ For each independent *OBFC1* signal, the credible set contained the top sentinel variant (Figures S5A–S5D). Colocalization analysis was performed using coloc, a Bayesian posterior probability method that estimates the probability of shared signal across testing modalities at each variant.^49^ We report the posterior probability that the two signals are independent (PPH3) and the posterior probability that the two signals overlap (PPH4). The sentinel variants from each signal were assayed as expression quantitative trait loci (eQTLs) in both GTEx^82^ and eQTLGen^38^ datasets. For each sentinel, significant gene-tissue pairs for that sentinel were identified from GTEx v8 (FDR < 0.05) and assayed for colocalization comparing the beta and standard error of the beta from our GWAS results and the eQTL results. For colocalization analysis in the eQTLGen dataset, all eGenes within a 2Mb window of the sentinel were identified and assayed for colocalization comparing the MAF, p value, and number of observations. MAF was estimated for eQTLGen data using the TOPMed MAF. Colocalization analysis was not possible for the *OBFC1* secondary signal as that variant is absent in both datasets and a representative proxy variant was not available. Roadmap^39^ data was accessed July, 2020 using the hg19 (February, 2009 release) UCSC genome browser^83^ track data hubs.^84^^,^^85^

#### Phenome-wide association tests (PheWAS)

Using individual level data within the Vanderbilt University biobank BioVU, PheWAS^86^ (tests for association between genotype and phenotype) were performed using the 49 (of 59) sentinel variants available in the multi-ethnic genotyping array (MEGA) chip results imputed to the Haplotype Reference Consortium.^87^ Single variant tests using SNP dosage values were performed for all available phecodes (number of cases at least 20), including the covariates age, sex, genotype batch and the first ten ancestry principal components. Analysis was performed separately in BioVU self-identified African Americans (AA, n = 15,174) and BioVU self-identified European Americans (EA, n = 70,439). In addition, European and African specific effect sizes from the joint analysis from Table 1 were combined to create separate polygenic trait scores (PTS) for each population group which were then tested for association with available phecodes, again including the covariates age, sex, genotype batch and the first ten ancestry principal components. Results were evaluated at a Bonferroni threshold corrected for the number of informative phecodes for each variant (range n = 1,114-1,361) or the PTS (n = 1,704) (Figure S6; Table S6). Analysis was performed using the PheWAS R package.^50^

We queried United Kingdom Biobank (UKBB) GWAS results using the University of Michigan PheWeb web interface (http://pheweb.sph.umich.edu/SAIGE-UKB/). The UKBB PheWeb interface contains results from a SAIGE^88^ genetic analysis of 1,403 ICD-based traits of 408,961 UKBB participants of European ancestry. PheWeb is a publicly accessible database that allows querying genome-wide association results for 28 million imputed genetic variants. 47 out of our 59 sentinel variants were present in PheWeb. We report all hits passing a Bonferroni correction for the number of tests performed for each variant (0.05/1403 = 3.6x10^−5^, Table S7).

## Data Availability

TOPMed genomic data and pre-existing parent study phenotypic data are made available to the scientific community in study-specific accessions in the database of Genotypes and Phenotypes (dbGaP) (https://www.ncbi.nlm.nih.gov/gap/?term=TOPMed). Telomere length calls were derived from the raw sequence data as described in the Method details, and the phenotype covariates of age, sex, and ancestry are available through the study-specific dbGaP accession IDs as listed in the supplementary information, Table S8. This TOPMed work includes multiple studies, some of which are based on sensitive populations, precluding the unrestricted sharing of GWAS summary statistics. The TOPMed dbGaP accession (dbGaP: phs001974.v3.p1) provides a mechanism for sharing sensitive results, using the controlled-access mechanism of dbGaP to provide protections for sensitive populations. The full results from this GWAS for the full group, European, African, Asian and Hispanic/Latino subgroup analyses have all been deposited at phs001974.v3.p1 and are available publicly through dbGaP access. All original code has been deposited at Zenodo (Zenodo: https://doi.org/10.5281/zenodo.5360775) and is publicly available. Any additional information required to reanalyze the data reported in this paper is available from the lead contact upon request.

## Supplemental information

Document S1. Figures S1–S6, Tables S1–S8, Note S1, Study Acknowledgments, Other Acknowledgments, TOPMed Consortium member list, TOPMed Hematology and Hemostasis Working Group list, and TOPMed Structural Variation Working Group list

Document S2. Article plus supplemental information

## References

[1] Aviv A., Shay J.W. (2018). Reflections on telomere dynamics and ageing-related diseases in humans. Philos. Trans. R. Soc. Lond. B Biol. Sci..

[2] McNally E.J., Luncsford P.J., Armanios M. (2019). Long telomeres and cancer risk: the price of cellular immortality. J. Clin. Invest..

[3] Codd V., Nelson C.P., Albrecht E., Mangino M., Deelen J., Buxton J.L., Hottenga J.J., Fischer K., Esko T., Surakka I. (2013). Identification of seven loci affecting mean telomere length and their association with disease. Nat. Genet..

[4] Codd V., Mangino M., van der Harst P., Braund P.S., Kaiser M., Beveridge A.J., Rafelt S., Moore J., Nelson C., Soranzo N., Wellcome Trust Case Control Consortium (2010). Common variants near TERC are associated with mean telomere length. Nat. Genet..

[5] Delgado D.A., Zhang C., Chen L.S., Gao J., Roy S., Shinkle J., Sabarinathan M., Argos M., Tong L., Ahmed A. (2018). Genome-wide association study of telomere length among South Asians identifies a second RTEL1 association signal. J. Med. Genet..

[6] Gu J., Chen M., Shete S., Amos C.I., Kamat A., Ye Y., Lin J., Dinney C.P., Wu X. (2011). A genome-wide association study identifies a locus on chromosome 14q21 as a predictor of leukocyte telomere length and as a marker of susceptibility for bladder cancer. Cancer Prev. Res. (Phila.).

[7] Lee J.H., Cheng R., Honig L.S., Feitosa M., Kammerer C.M., Kang M.S., Schupf N., Lin S.J., Sanders J.L., Bae H. (2014). Genome wide association and linkage analyses identified three loci-4q25, 17q23.2, and 10q11.21-associated with variation in leukocyte telomere length: the Long Life Family Study. Front. Genet..

[8] Levy D., Neuhausen S.L., Hunt S.C., Kimura M., Hwang S.J., Chen W., Bis J.C., Fitzpatrick A.L., Smith E., Johnson A.D. (2010). Genome-wide association identifies OBFC1 as a locus involved in human leukocyte telomere biology. Proc. Natl. Acad. Sci. USA.

[9] Liu Y., Cao L., Li Z., Zhou D., Liu W., Shen Q., Wu Y., Zhang D., Hu X., Wang T. (2014). A genome-wide association study identifies a locus on TERT for mean telomere length in Han Chinese. PLoS ONE.

[10] Mangino M., Christiansen L., Stone R., Hunt S.C., Horvath K., Eisenberg D.T., Kimura M., Petersen I., Kark J.D., Herbig U. (2015). DCAF4, a novel gene associated with leucocyte telomere length. J. Med. Genet..

[11] Mangino M., Hwang S.J., Spector T.D., Hunt S.C., Kimura M., Fitzpatrick A.L., Christiansen L., Petersen I., Elbers C.C., Harris T. (2012). Genome-wide meta-analysis points to CTC1 and ZNF676 as genes regulating telomere homeostasis in humans. Hum. Mol. Genet..

[12] Mangino M., Richards J.B., Soranzo N., Zhai G., Aviv A., Valdes A.M., Samani N.J., Deloukas P., Spector T.D. (2009). A genome-wide association study identifies a novel locus on chromosome 18q12.2 influencing white cell telomere length. J. Med. Genet..

[13] Pooley K.A., Bojesen S.E., Weischer M., Nielsen S.F., Thompson D., Amin Al Olama A., Michailidou K., Tyrer J.P., Benlloch S., Brown J. (2013). A genome-wide association scan (GWAS) for mean telomere length within the COGS project: identified loci show little association with hormone-related cancer risk. Hum. Mol. Genet..

[14] Prescott J., Kraft P., Chasman D.I., Savage S.A., Mirabello L., Berndt S.I., Weissfeld J.L., Han J., Hayes R.B., Chanock S.J. (2011). Genome-wide association study of relative telomere length. PLoS ONE.

[15] Saxena R., Bjonnes A., Prescott J., Dib P., Natt P., Lane J., Lerner M., Cooper J.A., Ye Y., Li K.W. (2014). Genome-wide association study identifies variants in casein kinase II (CSNK2A2) to be associated with leukocyte telomere length in a Punjabi Sikh diabetic cohort. Circ. Cardiovasc. Genet..

[16] Walsh K.M., Codd V., Smirnov I.V., Rice T., Decker P.A., Hansen H.M., Kollmeyer T., Kosel M.L., Molinaro A.M., McCoy L.S., ENGAGE Consortium Telomere Group (2014). Variants near TERT and TERC influencing telomere length are associated with high-grade glioma risk. Nat. Genet..

[17] Zeiger A.M., White M.J., Eng C., Oh S.S., Witonsky J., Goddard P.C., Contreras M.G., Elhawary J.R., Hu D., Mak A.C.Y. (2018). Genetic Determinants of Telomere Length in African American Youth. Sci. Rep..

[18] Dorajoo R., Chang X., Gurung R.L., Li Z., Wang L., Wang R., Beckman K.B., Adams-Haduch J., M Y., Liu S. (2019). Loci for human leukocyte telomere length in the Singaporean Chinese population and trans-ethnic genetic studies. Nat. Commun..

[19] Li C., Stoma S., Lotta L.A., Warner S., Albrecht E., Allione A., Arp P.P., Broer L., Buxton J.L., Da Silva Couto Alves A. (2020). Genome-wide Association Analysis in Humans Links Nucleotide Metabolism to Leukocyte Telomere Length. Am. J. Hum. Genet..

[20] Ding Z., Mangino M., Aviv A., Spector T., Durbin R., UK10K Consortium (2014). Estimating telomere length from whole genome sequence data. Nucleic Acids Res..

[21] Kimura M., Stone R.C., Hunt S.C., Skurnick J., Lu X., Cao X., Harley C.B., Aviv A. (2010). Measurement of telomere length by the Southern blot analysis of terminal restriction fragment lengths. Nat. Protoc..

[22] Alder J.K., Hanumanthu V.S., Strong M.A., DeZern A.E., Stanley S.E., Takemoto C.M., Danilova L., Applegate C.D., Bolton S.G., Mohr D.W. (2018). Diagnostic utility of telomere length testing in a hospital-based setting. Proc. Natl. Acad. Sci. USA.

[23] Almasy L., Blangero J. (1998). Multipoint quantitative-trait linkage analysis in general pedigrees. Am. J. Hum. Genet..

[24] Fang H., Hui Q., Lynch J., Honerlaw J., Assimes T.L., Huang J., Vujkovic M., Damrauer S.M., Pyarajan S., Gaziano J.M., VA Million Veteran Program (2019). Harmonizing Genetic Ancestry and Self-identified Race/Ethnicity in Genome-wide Association Studies. Am. J. Hum. Genet..

[25] Zhang M., Wang R., Wang Y., Diao F., Lu F., Gao D., Chen D., Zhai Z., Shu H. (2009). The CXXC finger 5 protein is required for DNA damage-induced p53 activation. Sci. China C Life Sci..

[26] Kaul R., Mukherjee S., Ahmed F., Bhat M.K., Chhipa R., Galande S., Chattopadhyay S. (2003). Direct interaction with and activation of p53 by SMAR1 retards cell-cycle progression at G2/M phase and delays tumor growth in mice. Int. J. Cancer.

[27] Bulik-Sullivan B.K., Loh P.R., Finucane H.K., Ripke S., Yang J., Patterson N., Daly M.J., Price A.L., Neale B.M., Schizophrenia Working Group of the Psychiatric Genomics Consortium (2015). LD Score regression distinguishes confounding from polygenicity in genome-wide association studies. Nat. Genet..

[28] Bulik-Sullivan B., Finucane H.K., Anttila V., Gusev A., Day F.R., Loh P.R., Duncan L., Perry J.R., Patterson N., Robinson E.B., ReproGen Consortium, Psychiatric Genomics Consortium, Genetic Consortium for Anorexia Nervosa of the Wellcome Trust Case Control Consortium 3 (2015). An atlas of genetic correlations across human diseases and traits. Nat. Genet..

[29] Cochran W.G. (1954). The combination of estimates from different experiments. Biometrics.

[30] Stuart B.D., Choi J., Zaidi S., Xing C., Holohan B., Chen R., Choi M., Dharwadkar P., Torres F., Girod C.E. (2015). Exome sequencing links mutations in PARN and RTEL1 with familial pulmonary fibrosis and telomere shortening. Nat. Genet..

[31] Tummala H., Walne A., Collopy L., Cardoso S., de la Fuente J., Lawson S., Powell J., Cooper N., Foster A., Mohammed S. (2015). Poly(A)-specific ribonuclease deficiency impacts telomere biology and causes dyskeratosis congenita. J. Clin. Invest..

[32] Touzot F., Callebaut I., Soulier J., Gaillard L., Azerrad C., Durandy A., Fischer A., de Villartay J.P., Revy P. (2010). Function of Apollo (SNM1B) at telomere highlighted by a splice variant identified in a patient with Hoyeraal-Hreidarsson syndrome. Proc. Natl. Acad. Sci. USA.

[33] van Overbeek M., de Lange T. (2006). Apollo, an Artemis-related nuclease, interacts with TRF2 and protects human telomeres in S phase. Curr. Biol..

[34] Lenain C., Bauwens S., Amiard S., Brunori M., Giraud-Panis M.J., Gilson E. (2006). The Apollo 5′ exonuclease functions together with TRF2 to protect telomeres from DNA repair. Curr. Biol..

[35] Wu M., Reuter M., Lilie H., Liu Y., Wahle E., Song H. (2005). Structural insight into poly(A) binding and catalytic mechanism of human PARN. EMBO J..

[36] Stewart J.A., Wang Y., Ackerson S.M., Schuck P.L. (2018). Emerging roles of CST in maintaining genome stability and human disease. Front. Biosci..

[37] Battle A., Brown C.D., Engelhardt B.E., Montgomery S.B., GTEx Consortium, Laboratory, Data Analysis & Coordinating Center (LDACC)—Analysis Working Group, Statistical Methods Groups—Analysis Working Group, Enhancing GTEx (eGTEx) Groups, NIH Common Fund, NIH/NCI, NIH/NHGRI, NIH/NIMH, NIH/NIDA, Biospecimen Collection Source Site—NDRI, Biospecimen Collection Source Site—RPCI, Biospecimen Core Resource—VARI, Brain Bank Repository—University of Miami Brain Endowment Bank, Leidos Biomedical—Project Management, ELSI Study, Genome Browser Data Integration & Visualization—EBI, Genome Browser Data Integration & Visualization—UCSC Genomics Institute, University of California, Santa Cruz, Lead Analysts, Laboratory, Data Analysis & Coordinating Center (LDACC), NIH Program Management, Biospecimen Collection, Pathology, eQTL Manuscript Working Group (2017). Genetic effects on gene expression across human tissues. Nature.

[38] Vosa U., Claringbould A., Westra H.-J., Bonder M.J., Deelen P., Zeng B., Kirsten H., Saha A., Kreuzhuber R., Yazar S. (2021). Unraveling the polygenic architecture of complex traits using blood eQTL metaanalysis. Nat. Genet..

[39] Kundaje A., Meuleman W., Ernst J., Bilenky M., Yen A., Heravi-Moussavi A., Kheradpour P., Zhang Z., Wang J., Ziller M.J., Roadmap Epigenomics Consortium (2015). Integrative analysis of 111 reference human epigenomes. Nature.

[40] Januszewski A.S., Sutanto S.S., McLennan S., O’Neal D.N., Keech A.C., Twigg S.M., Jenkins A.J. (2016). Shorter telomeres in adults with type 1 diabetes correlate with diabetes duration, but only weakly with vascular function and risk factors. Diabetes Res. Clin. Pract..

[41] Oglesbee M.J., Herdman A.V., Passmore G.G., Hoffman W.H. (2005). Diabetic ketoacidosis increases extracellular levels of the major inducible 70-kDa heat shock protein. Clin. Biochem..

[42] Nussey D.H., Baird D., Barrett E., Boner W., Fairlie J., Gemmell N., Hartmann N., Horn T., Haussmann M., Olsson M. (2014). Measuring telomere length and telomere dynamics in evolutionary biology and ecology. Methods Ecol. Evol..

[43] Aubert G., Hills M., Lansdorp P.M. (2012). Telomere length measurement-caveats and a critical assessment of the available technologies and tools. Mutat. Res..

[44] Lee M., Napier C.E., Yang S.F., Arthur J.W., Reddel R.R., Pickett H.A. (2017). Comparative analysis of whole genome sequencing-based telomere length measurement techniques. Methods.

[45] Demanelis K., Jasmine F., Chen L.S., Chernoff M., Tong L., Delgado D., Zhang C., Shinkle J., Sabarinathan M., Lin H., GTEx Consortium (2020). Determinants of telomere length across human tissues. Science.

[46] Taliun D., Harris D.N., Kessler M.D., Carlson J., Szpiech Z.A., Torres R., Taliun S.A.G., Corvelo A., Gogarten S.M., Kang H.M., NHLBI Trans-Omics for Precision Medicine (TOPMed) Consortium (2021). Sequencing of 53,831 diverse genomes from the NHLBI TOPMed Program. Nature.

[47] Gogarten S.M., Sofer T., Chen H., Yu C., Brody J.A., Thornton T.A., Rice K.M., Conomos M.P. (2019). Genetic association testing using the GENESIS R/Bioconductor package. Bioinformatics.

[48] Hormozdiari F., Kostem E., Kang E.Y., Pasaniuc B., Eskin E. (2014). Identifying causal variants at loci with multiple signals of association. Genetics.

[49] Giambartolomei C., Vukcevic D., Schadt E.E., Franke L., Hingorani A.D., Wallace C., Plagnol V. (2014). Bayesian test for colocalisation between pairs of genetic association studies using summary statistics. PLoS Genet..

[50] Carroll R.J., Bastarache L., Denny J.C. (2014). R PheWAS: data analysis and plotting tools for phenome-wide association studies in the R environment. Bioinformatics.

[51] NHLBI Trans-Omics for Precision Medicine (2021). https://www.nhlbiwgs.org/group/project-studies.

[52] Jun G., Wing M.K., Abecasis G.R., Kang H.M. (2015). An efficient and scalable analysis framework for variant extraction and refinement from population-scale DNA sequence data. Genome Res..

[53] Nersisyan L., Arakelyan A. (2015). Computel: computation of mean telomere length from whole-genome next-generation sequencing data. PLoS ONE.

[54] Aviv A., Hunt S.C., Lin J., Cao X., Kimura M., Blackburn E. (2011). Impartial comparative analysis of measurement of leukocyte telomere length/DNA content by Southern blots and qPCR. Nucleic Acids Res..

[55] O’Callaghan N.J., Fenech M. (2011). A quantitative PCR method for measuring absolute telomere length. Biol. Proced. Online.

[56] Mwasongwe S., Gao Y., Griswold M., Wilson J.G., Aviv A., Reiner A.P., Raffield L.M. (2017). Leukocyte telomere length and cardiovascular disease in African Americans: the Jackson Heart Study. Atherosclerosis.

[57] Leek J.T., Storey J.D. (2007). Capturing heterogeneity in gene expression studies by surrogate variable analysis. PLoS Genet..

[58] Stegle O., Parts L., Piipari M., Winn J., Durbin R. (2012). Using probabilistic estimation of expression residuals (PEER) to obtain increased power and interpretability of gene expression analyses. Nat. Protoc..

[59] Pedersen B.S., Quinlan A.R. (2018). Mosdepth: quick coverage calculation for genomes and exomes. Bioinformatics.

[60] Derrien T., Estellé J., Marco Sola S., Knowles D.G., Raineri E., Guigó R., Ribeca P. (2012). Fast computation and applications of genome mappability. PLoS ONE.

[61] sv_blacklist.bed. http://cf.10xgenomics.com/supp/genome/GRCh38/sv_blacklist.bed.

[62] Halko N., Martinsson P.G., Tropp J.A. (2011). Finding Structure with Randomness: Probabilistic Algorithms for Constructing Approximate Matrix Decompositions. SIAM Rev..

[63] Conomos M.P., Miller M.B., Thornton T.A. (2015). Robust inference of population structure for ancestry prediction and correction of stratification in the presence of relatedness. Genet. Epidemiol..

[64] Sofer T., Zheng X., Gogarten S.M., Laurie C.A., Grinde K., Shaffer J.R., Shungin D., O’Connell J.R., Durazo-Arvizo R.A., Raffield L., NHLBI Trans-Omics for Precision Medicine (TOPMed) Consortium (2019). A fully adjusted two-stage procedure for rank-normalization in genetic association studies. Genet. Epidemiol..

[65] Conomos M.P., Reiner A.P., Weir B.S., Thornton T.A. (2016). Model-free Estimation of Recent Genetic Relatedness. Am. J. Hum. Genet..

[66] Tang Z.Z., Lin D.Y. (2015). Meta-analysis for Discovering Rare-Variant Associations: Statistical Methods and Software Programs. Am. J. Hum. Genet..

[67] Zhou B., Shi J., Whittemore A.S. (2011). Optimal methods for meta-analysis of genome-wide association studies. Genet. Epidemiol..

[68] The NHLBI Trans-Omics for Precision Medicine (TOPMed) Whole Genome Sequencing Program (2018). https://bravo.sph.umich.edu/freeze5/hg38/.

[69] Cochran W.G. (1954). The Combination of Estimates from Different Experiments. Biometrics.

[70] Wilson J.G., Rotimi C.N., Ekunwe L., Royal C.D., Crump M.E., Wyatt S.B., Steffes M.W., Adeyemo A., Zhou J., Taylor H.A. (2005). Study design for genetic analysis in the Jackson Heart Study. Ethn. Dis..

[71] Frankish A., Diekhans M., Ferreira A.M., Johnson R., Jungreis I., Loveland J., Mudge J.M., Sisu C., Wright J., Armstrong J. (2019). GENCODE reference annotation for the human and mouse genomes. Nucleic Acids Res..

[72] Liu X., White S., Peng B., Johnson A.D., Brody J.A., Li A.H., Huang Z., Carroll A., Wei P., Gibbs R. (2016). WGSA: an annotation pipeline for human genome sequencing studies. J. Med. Genet..

[73] Ahn D.H., Ozer H.G., Hancioglu B., Lesinski G.B., Timmers C., Bekaii-Saab T. (2016). Whole-exome tumor sequencing study in biliary cancer patients with a response to MEK inhibitors. Oncotarget.

[74] Ioannidis N.M., Rothstein J.H., Pejaver V., Middha S., McDonnell S.K., Baheti S., Musolf A., Li Q., Holzinger E., Karyadi D. (2016). REVEL: An Ensemble Method for Predicting the Pathogenicity of Rare Missense Variants. Am. J. Hum. Genet..

[75] Jagadeesh K.A., Wenger A.M., Berger M.J., Guturu H., Stenson P.D., Cooper D.N., Bernstein J.A., Bejerano G. (2016). M-CAP eliminates a majority of variants of uncertain significance in clinical exomes at high sensitivity. Nat. Genet..

[76] Kircher M., Witten D.M., Jain P., O’Roak B.J., Cooper G.M., Shendure J. (2014). A general framework for estimating the relative pathogenicity of human genetic variants. Nat. Genet..

[77] Graham G. (2015). Disparities in cardiovascular disease risk in the United States. Curr. Cardiol. Rev..

[78] Chen H., Huffman J.E., Brody J.A., Wang C., Lee S., Li Z., Gogarten S.M., Sofer T., Bielak L.F., Bis J.C., NHLBI Trans-Omics for Precision Medicine (TOPMed) Consortium, TOPMed Hematology and Hemostasis Working Group (2019). Efficient Variant Set Mixed Model Association Tests for Continuous and Binary Traits in Large-Scale Whole-Genome Sequencing Studies. Am. J. Hum. Genet..

[79] Brody J.A., Morrison A.C., Bis J.C., O’Connell J.R., Brown M.R., Huffman J.E., Ames D.C., Carroll A., Conomos M.P., Gabriel S., NHLBI Trans-Omics for Precision Medicine (TOPMed) Consortium, Cohorts for Heart and Aging Research in Genomic Epidemiology (CHARGE) Consortium, TOPMed Hematology and Hemostasis Working Group, CHARGE Analysis and Bioinformatics Working Group (2017). Analysis commons, a team approach to discovery in a big-data environment for genetic epidemiology. Nat. Genet..

[80] Wu M.C., Lee S., Cai T., Li Y., Boehnke M., Lin X. (2011). Rare-variant association testing for sequencing data with the sequence kernel association test. Am. J. Hum. Genet..

[81] Keramati A.R., Yanek L.R., Iyer K., Taub M.A., Ruczinski I., Becker D.M., Becker L.C., Faraday N., Mathias R.A. (2019). Targeted deep sequencing of the PEAR1 locus for platelet aggregation in European and African American families. Platelets.

[82] GTEx Consortium (2020). The GTEx Consortium atlas of genetic regulatory effects across human tissues. Science.

[83] Kent W.J., Sugnet C.W., Furey T.S., Roskin K.M., Pringle T.H., Zahler A.M., Haussler D. (2002). The human genome browser at UCSC. Genome Res..

[84] Li D., Hsu S., Purushotham D., Sears R.L., Wang T. (2019). WashU Epigenome Browser update 2019. Nucleic Acids Res..

[85] Raney B.J., Dreszer T.R., Barber G.P., Clawson H., Fujita P.A., Wang T., Nguyen N., Paten B., Zweig A.S., Karolchik D., Kent W.J. (2014). Track data hubs enable visualization of user-defined genome-wide annotations on the UCSC Genome Browser. Bioinformatics.

[86] Denny J.C., Ritchie M.D., Basford M.A., Pulley J.M., Bastarache L., Brown-Gentry K., Wang D., Masys D.R., Roden D.M., Crawford D.C. (2010). PheWAS: demonstrating the feasibility of a phenome-wide scan to discover gene-disease associations. Bioinformatics.

[87] McCarthy S., Das S., Kretzschmar W., Delaneau O., Wood A.R., Teumer A., Kang H.M., Fuchsberger C., Danecek P., Sharp K., Haplotype Reference Consortium (2016). A reference panel of 64,976 haplotypes for genotype imputation. Nat. Genet..

[88] Dey R., Schmidt E.M., Abecasis G.R., Lee S. (2017). A Fast and Accurate Algorithm to Test for Binary Phenotypes and Its Application to PheWAS. Am. J. Hum. Genet..

