# Supplementary material for: Genetic determinants of telomere length from 109,122 ancestrally diverse whole-genome sequences in TOPMed
Source: Cell Genom. 2022 Jan 13;2(1):100084. doi: 10.1016/j.xgen.2021.100084 (PMC9075703; doi:10.1016/j.xgen.2021.100084)
Supplement: Document S1. Figures S1–S6, Tables S1–S8, Note S1, Study Acknowledgments, Other Acknowledgments, TOPMed Consortium member list, TOPMed Hematology and Hemostasis Working Group list, and TOPMed Structural Variation Working Group list [file mmc1.pdf]

Cell Genomics, Volume *2*

Supplemental information

Genetic determinants of telomere length from 109,122 ancestrally diverse whole-genome sequences in TOPMed

Margaret A. Taub, Matthew P. Conomos, Rebecca Keener, Kruthika R. Iyer, Joshua S. Weinstock, Lisa R. Yanek, John Lane, Tyne W. Miller-Fleming, Jennifer A. Brody, Laura M. Raffield, Caitlin P. McHugh, Deepti Jain, Stephanie M. Gogarten, Cecelia A. Laurie, Ali Keramati, Marios Arvanitis, Albert V. Smith, Benjamin Heavner, Lucas Barwick, Lewis C. Becker, Joshua C. Bis, John Blangero, Eugene R. Bleecker, Esteban G. Burchard, Juan C. Celedón, Yen Pei C. Chang, Brian Custer, Dawood Darbar, Lisa de las Fuentes, Dawn L. DeMeo, Barry I. Freedman, Melanie E. Garrett, Mark T. Gladwin, Susan R. Heckbert, Bertha A. Hidalgo, Marguerite R. Irvin, Talat Islam, W. Craig Johnson, Stefan Kaab, Lenore Launer, Jiwon Lee, Simin Liu, Arden Moscati, Kari E. North, Patricia A. Peyser, Nicholas Rafaels, Christine Seidman, Daniel E. Weeks, Fayun Wen, Marsha M. Wheeler, L. Keoki Williams, Ivana V. Yang, Wei Zhao, Stella Aslibekyan, Paul L. Auer, Donald W. Bowden, Brian E. Cade, Zhanghua Chen, Michael H. Cho, L. Adrienne Cupples, Joanne E. Curran, Michelle Daya, Ranjan Deka, Celeste Eng, Tasha E. Fingerlin, Xiuqing Guo, Lifang Hou, Shih-Jen Hwang, Jill M. Johnsen, Eimear E. Kenny, Albert M. Levin, Chunyu Liu, Ryan L. Minster, Take Naseri, Mehdi Nouraie, Muagututi`a Sefuiva Reupena, Ester C. Sabino, Jennifer A. Smith, Nicholas L. Smith, Jessica Lasky-Su, James G. Taylor VI, Marilyn J. Telen, Hemant K. Tiwari, Russell P. Tracy, Marquitta J. White, Yingze Zhang, Kerri L. Wiggins, Scott T. Weiss, Ramachandran S. Vasan, Kent D. Taylor, Moritz F. Sinner, Edwin K. Silverman, M. Benjamin Shoemaker, Wayne H.-H. Sheu, Frank Sciurba, David A. Schwartz, Jerome I. Rotter, Daniel Roden, Susan Redline, Benjamin A. Raby, Bruce M. Psaty, Juan M. Peralta, Nicholette D. Palmer, Sergei Nekhai, Courtney G. Montgomery, Braxton D. Mitchell, Deborah A. Meyers, Stephen T. McGarvey, Fernando D. Martinez on behalf of the NHLBI CARE Network, Angel C.Y. Mak, Ruth J.F. Loos, Rajesh Kumar, Charles Kooperberg, Barbara A. Konkle, Shannon Kelly, Sharon L.R. Kardia, Robert Kaplan, Jiang He, Hongsheng Gui, Frank D. Gilliland, Bruce D. Gelb, Myriam Fornage, Patrick T. Ellinor, Mariza de Andrade, Adolfo Correa, Yii-Der Ida Chen, Eric Boerwinkle, Kathleen C. Barnes, Allison E. Ashley-Koch, Donna K. Arnett, Christine Albert, NHLBI Trans-Omics for Precision Medicine (TOPMed) Consortium, TOPMed Hematology and Hemostasis Working Group, TOPMed Structural Variation Working Group, Cathy C. Laurie, Goncalo Abecasis, Deborah A. Nickerson, James G. Wilson, Stephen S. Rich, Daniel Levy, Ingo Ruczinski, Abraham Aviv, Thomas W. Blackwell, Timothy Thornton, Jeff O'Connell, Nancy J. Cox, James A. Perry, Mary Armanios, Alexis Battle, Nathan Pankratz, Alexander P. Reiner, and Rasika A. Mathias

LIST OF CONTENTS: Supplementary Information for Genetic determinants of telomere length from 109,122 ancestrally-diverse whole genome sequences in TOPMed

Supplementary Figures:

Figure S1, Assessment of bioinformatic telomere length estimation

Figure S2, Replication of variants in newly identified loci

Figure S3, Leave-one-out analysis of gene-based results

Figure S4, Detailed results from the *OBFC1* locus

Figure S5, Credible set analysis and colocalization analysis for *OBFC1* in eQTLGen

Figure S6, Polygenic trait score (PTS) analysis across 49 available sentinel variants in BioVU

Supplementary Tables:

Table S1, Demographics by TOPMed study

Table S2, Conditional analysis results by chromosome

Table S3, Results at prior GWAS variants

Table S4, Association results for the single variant association analysis for each of the 59 sentinel variants from *Table 1*

Table S5, Iterative conditional analysis of *OBFC1*

Table S6, BioVU PheWAS results

Table S7, UKBB PheWAS results

Supplemental Note 1

Study Acknowledgements

Other Acknowledgements

Consortium Memberships

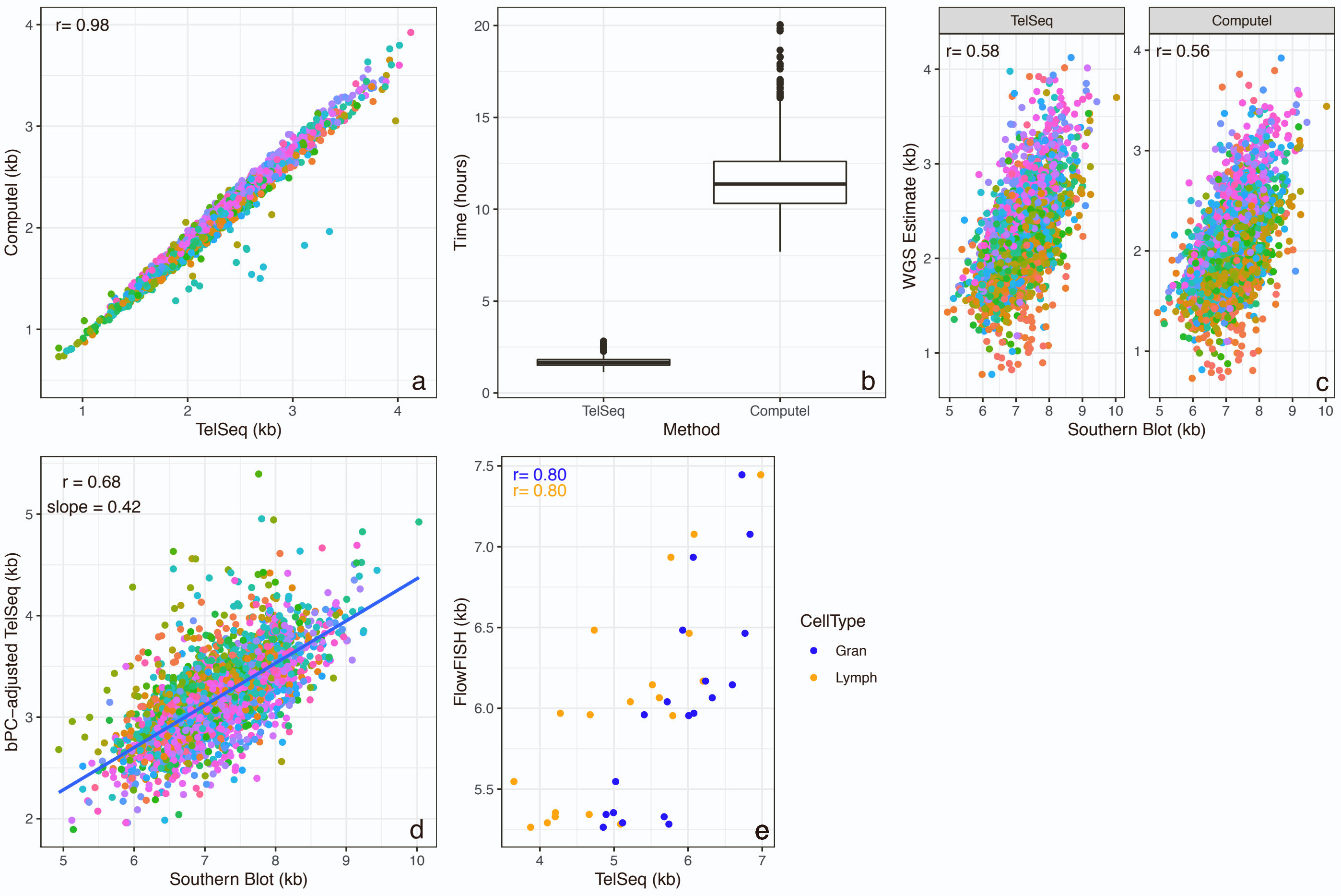

Figure S1, Assessment of bioinformatic telomere length estimation: For 2,398 samples from the Jackson Heart Study (JHS), **[a]** scatter plot with Pearson correlation between TelSeq and Computel length estimates; **[b]** Comparison of computational times for TelSeq and Computel; **[c]** scatter plots with Pearson correlations between TelSeq (left) and Computel (right) and Southern blot TL estimates; **[d]** scatter plot with Pearson correlation and linear regression slope between TelSeq and Southern blot TL estimates after adjustment for the final set of 200 batch principal components (bPCs) used in our full analysis. Colors indicate on which plate samples were shipped to the sequencing center, in Panels A, C and D; and **[e]** scatter plot with Pearson correlation between bPC-adjusted TelSeq and flowFISH data on 19 samples from the GeneSTAR study. Related to ***STAR Methods***.

a

Chr	Position	Locus	SNP	TOPMed effect size in bp	Li et al. 2020 European (N=78,592) P-value	Beta	SE(Beta)	Dorajoo et al. 2019 Singaporean Chinese (N=23,096) P-value	Beta	SE(Beta)	Replication threshold
1	35259602	ZMYM4	rs11581846	-19.9	2.53E-01	-0.01	0.01	6.09E-01	-0.01	0.01	>0.05
1	113881455	BCL2L15	rs2296176	-19.5	1.69E-04	-0.02	0.01	6.86E-05	-0.05	0.01	<0.0026
3	117584223	LINC00901	rs961617801	1009.6	-	-	-	-	-	-	-
3	190053412	P3H2	rs10937417	14.6	2.49E-01	0.01	0.01	6.80E-01	0.00	0.01	>0.05
4	9928595	SLC2A2	rs4235345	16.8	5.62E-02	0.01	0.01	9.13E-01	0.00	0.03	>0.05
5	139637905	CXXC5	rs75903170	29.6	3.15E-05	0.05	0.01	3.40E-04	0.06	0.02	<0.0026
6	31815431	HSPA1A	rs1008438	-20.3	9.38E-05	-0.02	0.01	4.47E-03	-0.03	0.01	<0.0026
7	129041243	TNP03	rs7783384	-15.2	8.29E-03	-0.01	0.01	2.80E-02	-0.02	0.01	<0.05
10	94344908	NOC3L	rs3758526	-22.0	6.31E-05	-0.03	0.01	1.68E-02	-0.02	0.01	<0.0026
10	99514276	NKX2-3	rs10883359	-16.5	1.50E-03	-0.02	0.01	2.18E-07	-0.05	0.01	<0.0026
13	41150640	KBTBD7	rs1411041	22.4	6.38E-03	0.02	0.01	5.85E-03	0.03	0.01	<0.05
15	50065546	ATP8B4	rs7172615	-17.8	2.88E-07	-0.03	0.01	2.10E-03	-0.03	0.01	<0.0026
16	70193527	CLEC18C	rs62049363	-16.8	4.87E-04	-0.03	0.01	1.38E-03	-0.03	0.01	<0.0026
16	87961594	BANP	rs12934497	14.6	3.84E-02	0.01	0.00	5.10E-01	0.02	0.02	<0.05
18	650764		rs150119891	-98.8	2.81E-01	-0.03	0.03	-	-	-	>0.05
18	666625	TYMS	rs8088781	-50.6	2.41E-07	-0.04	0.01	-	-	-	<0.0026
18	676473		rs2612101	26.0	3.16E-02	-0.01	0.01	-	-	-	<0.05
18	44666476	SETBP1	rs2852770	-19.0	1.87E-01	-0.01	0.01	7.41E-01	0.00	0.01	>0.05
20	36922795	SAMHD1	rs2342113	-23.7	2.78E-05	-0.04	0.01	1.57E-02	-0.02	0.01	<0.0026
22	50532618	TYMP	rs361725	-15.6	1.72E-03	-0.02	0.01	5.41E-05	-0.04	0.01	<0.0026
X	66015290	VSIG4	rs12394264	19.0	-	-	-	-	-	-	-
X	154720412	GAB3	rs2728723	13.2	-	-	-	-	-	-	-

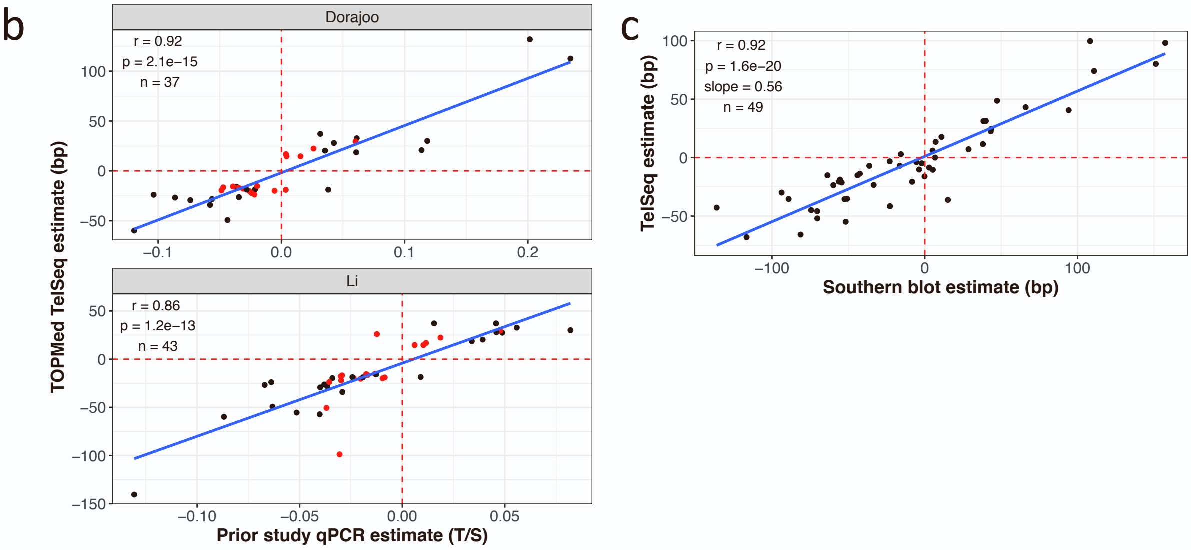

Figure S2, Replication of variants in newly identified loci. [a] Replication results for the 22 variants mapping to the 20 newly identified TOPMed loci in two non-overlapping, prior published GWAS on TL using qPCR data. Three thresholds of significance are labeled (Bonferroni significant: p <0.0026; nominally significant: p <0.05; non-significant: p >0.05). **[b]** Pearson correlation between the estimated effect sizes for all sentinel variants comparing the TOPMed trans-population analysis using TelSeq (bp) and the two non-overlapping, prior published GWAS on TL using qPCR data (T/S ratio). Points in red correspond to previously un-identified variants included in [a]. **[c]** Pearson correlation and linear regression slope between the estimated effect sizes from single variant association tests of the 2,398 individuals from the Jackson Heart Study with both TelSeq (bp) and Southern blot (bp) TL measurements. Numbers of variants shown in [b] represent those available for lookup, and in [c] represent those with MAF≥1% in the JHS sample set. Related to ***STAR Methods: Replication and comparison of effect size estimates.***

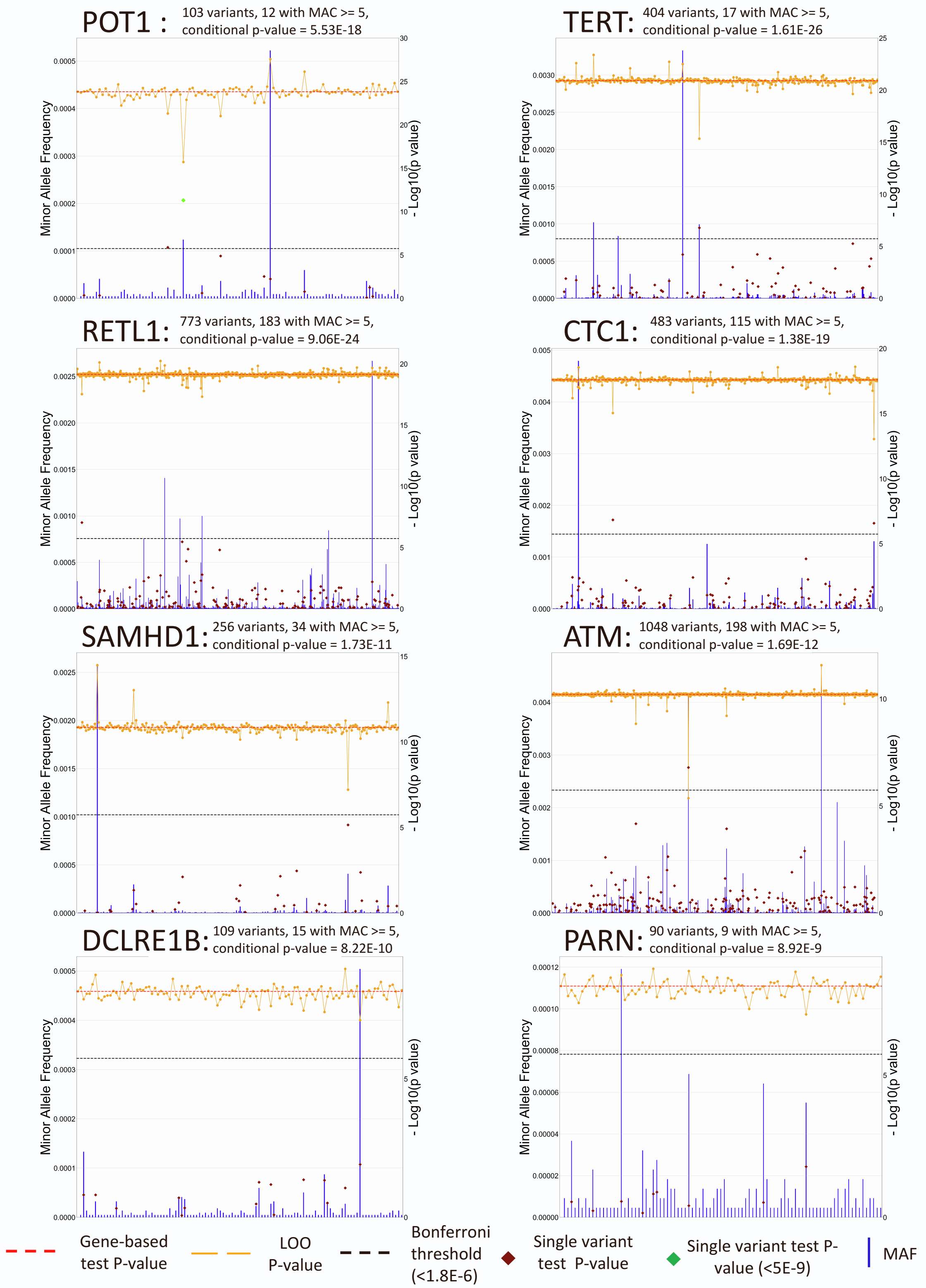

Figure S3, Leave-one-out analysis of gene-based results: Eight genes were identified as passing the Bonferroni threshold based on number of genes tested (p-value < 0.05/27,558 = $1.8x10^{-6}$). For each gene, a leave-one-out analysis was performed iterating the SMMAT test and leaving one variant out at a time. The plots show the change in SMMAT p-value for each variant (orange line with marker) relative to the variant's allele frequency (blue bar), the overall gene-based test including all variants (dotted red line) and the single variant results for all variants with an MAC≥5 that were included in single variant tests for association (brown and green diamonds). For each gene, the number of rare and deleterious variants included in SMMAT is indicated. For any variant with a MAC≥5, a single variant test was also performed as part of the primary analysis. The count of these variants is indicated. In addition the SMMAT p-value for these genes when conditioning on the 59 sentinel variants is also given. Related to ***STAR Methods.***

a

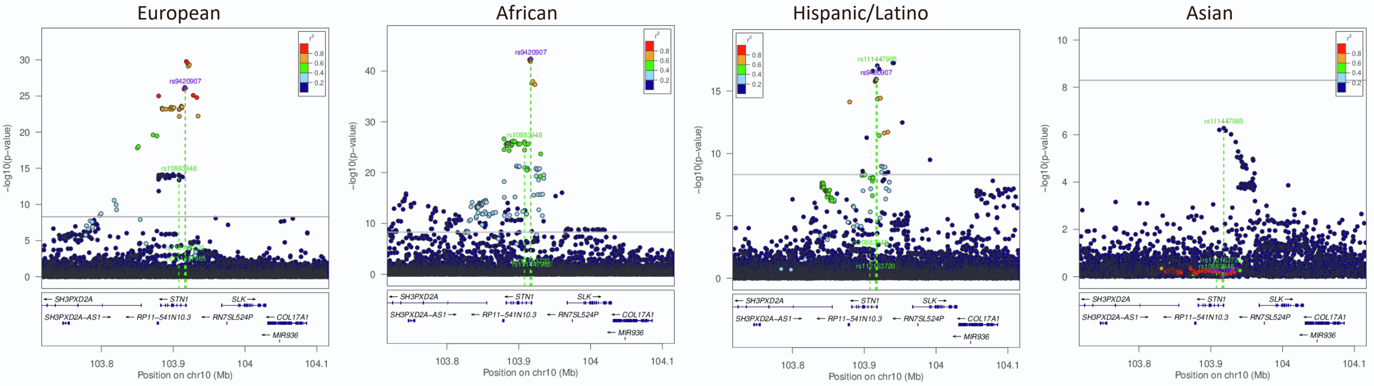

b

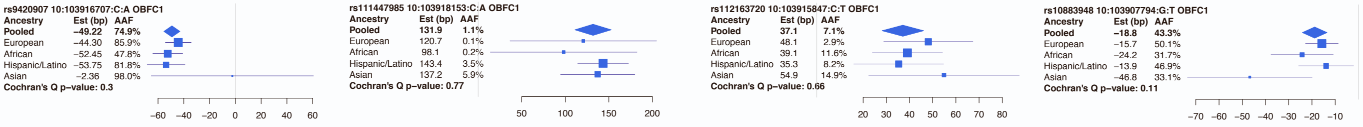

Figure S4, Detailed results from the *OBFC1* locus: [a] LocusZoom plots for four population groups for the *OBFC1* locus. Linkage disequilibrium (LD) was calculated from the set of samples used in the analysis with respect to the peak variant in the pooled trans-population primary analysis, thereby reflecting LD patterns specific to the TOPMed samples. For each figure, the peak sentinel variant from the pooled population analysis is indexed and labeled in purple, and all independent variants identified through the iterative conditional approach are labeled in green and highlighted with green dotted lines. **[b]** Forest plots displaying effect sizes and standard errors, as well as minor allele frequencies, by population group for the four sentinel variants in *OBFC1*. Related to ***Figure 3***.

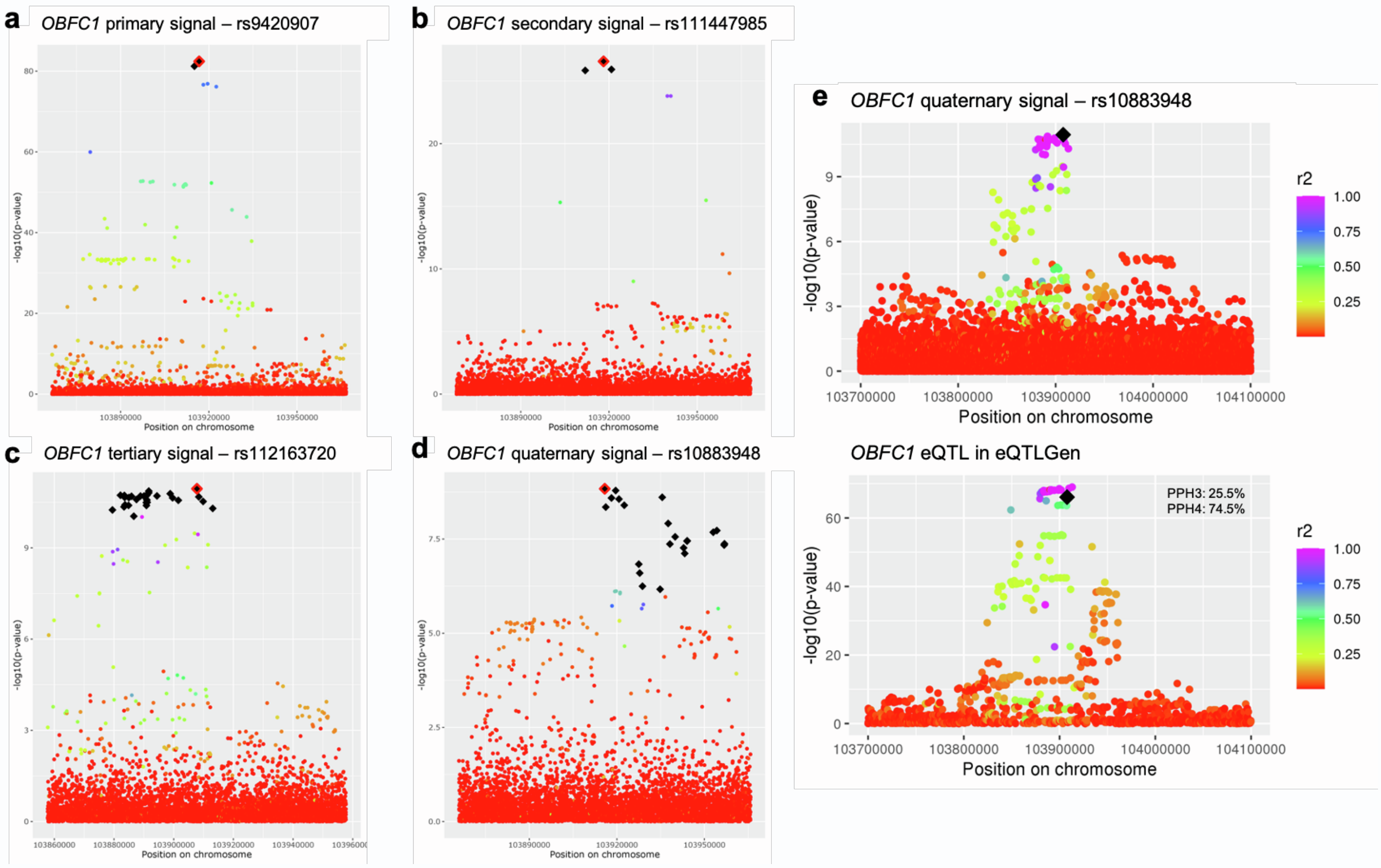

Figure S5, Credible set analysis and colocalization analysis for *OBFC1* in eQTLGen: [a-d] Manhattan plots for each *OBFC1* signal are shown where p-values were taken from the appropriate conditional analysis output and LD was calculated with respect to the sentinel variant. Credible set variants are indicated with black diamonds; the sentinel variant is indicated as a black diamond with red outline. **[e]** Manhattan plot for colocalization analysis of the *OBFC1* quaternary signal with an *OBFC1* eQTL in eQTLGen. PPH3 and PPH4 for colocalization are indicated in the top right corner of the eQTL plot. LD was calculated with respect to the sentinel, indicated with a black diamond on each graph, and with pooled trans-population analysis samples. Related to ***Figure 3***.

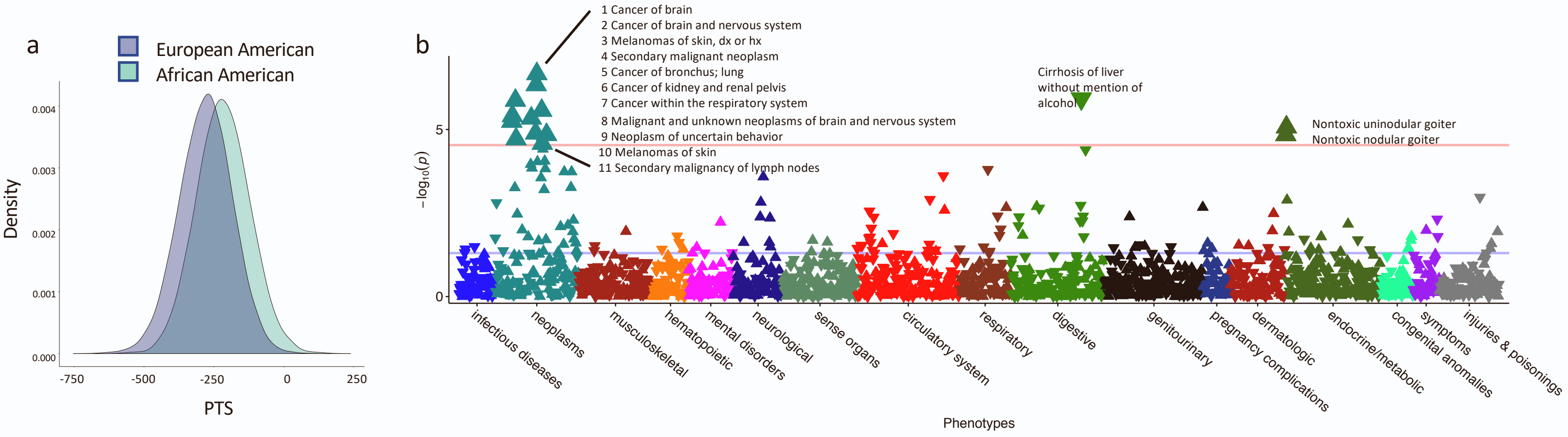

Figure S6, Polygenic trait score (PTS) analysis across 49 available sentinel variants in BioVU. **[a]** Smoothed distributions of PTS values for European Americans (n=70,439) and African Americans (n=15,174) from BioVU biobank. **[b]** Overview of the PheWAS in the BioVU European Americans. ▲ = higher PTS is associated with the phenotype. ▼ = higher PTS is protective against the phenotype. Related to ***STAR Methods: PheWAS results from Vanderbilt BioVU.***

Table S1, Demographics by TOPMed study: Sample demographics summarized by each set of analysis performed: pooled trans-population and four non-overlapping population groups (defined with HARE, using reported race/ethnicity and genetically inferred ancestry in combination; “Other/Uncertain” includes individuals with maximum stratum probability from HARE < 0.7, as well as Brazilians and Samoans who were excluded from the HARE analysis). Related to ***STAR Methods*** **.**

				HARE Population Group					Sequencing Center					
Study	Total Count	Male Count (Pct)	Mean Age (SD, Range)	European	African	Hispanic/Latino	Asian	Other/ Uncertain	Baylor	Broad	Psomagen	NYGC	UW	WASHU
AFLMU	350	266 (0.76)	48 (11.9, 19-79)	349	-	-	-	1	-	350	-	-	-	-
Amish	1109	557 (0.5)	52 (16.8, 20-91)	1109	-	-	-	-	-	1109	-	-	-	-
ARIC	3829	1833 (0.48)	58 (5.9, 44-79)	3578	245	5	-	1	3825	4	-	-	-	-
BioMe	9455	3804 (0.4)	56 (14.9, 19-90)	3180	2497	3212	428	138	6828	-	-	-	-	2627
CAMP	1662	860 (0.52)	31 (15, 9-66)	1270	223	131	21	17	-	-	-	-	1662	-
CARDIA	3396	1480 (0.44)	45 (7, 27-61)	1802	1578	7	3	6	3396	-	-	-	-	-
CARE_BADGER	35	23 (0.66)	11 (3.5, 6-18)	9	2	24	-	-	-	-	-	-	35	-
CARE_CLIC	12	7 (0.58)	11 (3.9, 6-17)	2	-	10	-	-	-	-	-	-	12	-
CARE_PACT	22	11 (0.5)	9 (2.4, 6-14)	6	2	12	-	2	-	-	-	-	22	-
CARE_TREXA	65	33 (0.51)	11 (3.3, 6-18)	16	1	46	-	2	-	-	-	-	65	-
CFS	1281	576 (0.45)	39 (19.6, 2-92)	612	664	4	1	-	-	-	-	-	1281	-
ChildrensHS_GAP	7	5 (0.71)	19 (0.6, 18-20)	2	-	5	-	-	-	-	-	-	7	-
ChildrensHS_IGERA	152	52 (0.34)	26 (2, 23-31)	50	3	94	-	5	-	-	-	-	152	-
ChildrensHS_MetaAir	52	26 (0.5)	19 (0.7, 18-21)	11	-	38	-	3	-	-	-	-	52	-
CHIRAH	263	96 (0.37)	19 (10.9, 8-40)	-	263	-	-	-	-	-	-	-	263	-
CHS	3510	1466 (0.42)	74 (5.7, 64-98)	2775	712	14	-	9	3510	-	-	-	-	-
COPDGene	10054	5327 (0.53)	59 (9.1, 39-85)	6785	3235	22	1	11	-	8241	-	-	1813	-
CRA	2982	1591 (0.53)	29 (15.2, 0-93)	-	-	2982	-	-	-	-	-	-	2982	-
DHS	372	159 (0.43)	60 (8.7, 36-86)	-	372	-	-	-	-	372	-	-	-	-
ECLIPSE	2308	1483 (0.64)	62 (7.9, 40-76)	2224	41	11	9	23	-	-	-	-	-	2308
EOCOPD	74	21 (0.28)	49 (5.8, 26-59)	73	-	-	-	1	-	-	-	-	74	-
FHS	4118	1887 (0.46)	61 (15.6, 20-98)	4102	-	1	1	14	-	4118	-	-	-	-
GALAI	893	417 (0.47)	19 (9.6, 8-55)	3	-	888	-	2	-	-	-	-	893	-
GALAII	3456	1706 (0.49)	13 (3.7, 4-60)	11	12	3410	-	23	-	-	-	986	2470	-
GeneSTAR	1485	606 (0.41)	44 (12.6, 20-79)	833	651	1	-	-	-	102	1383	-	-	-
GENOA	1188	362 (0.3)	62 (10, 25-95)	-	1188	-	-	-	-	101	-	-	1087	-
GenSalt	1823	961 (0.53)	39 (9.6, 15-62)	-	-	-	1823	-	1823	-	-	-	-	-
GOLDN	947	445 (0.47)	48 (16.4, 18-87)	946	-	1	-	-	-	-	-	-	947	-
HCHS_SOL	3854	1564 (0.41)	47 (14, 18-75)	40	59	3638	-	117	3854	-	-	-	-	-
HVH	691	452 (0.65)	64 (12, 22-92)	653	26	5	7	-	616	75	-	-	-	-
HyperGEN	1863	684 (0.37)	47 (12.8, 18-85)	1	1861	-	1	-	-	-	-	-	1863	-
IPF	309	185 (0.6)	65 (10.2, 25-91)	298	2	7	-	2	-	-	-	-	-	309
JHS	3324	1237 (0.37)	56 (12.9, 21-92)	-	3321	1	1	1	-	-	-	-	3324	-
LTRC	1340	695 (0.52)	63 (10.7, 21-88)	1226	83	25	4	2	-	1340	-	-	-	-
Mayo_VTE	1279	603 (0.47)	57 (16.8, 18-96)	1261	6	9	1	2	1279	-	-	-	-	-
MESA	5310	2519 (0.47)	61 (9.7, 39-91)	1861	1872	947	605	25	-	5310	-	-	-	-
MLOF	5094	4566 (0.9)	26 (19.2, 0-92)	3590	514	698	215	77	2924	-	-	2170	-	-
OMG_SCD	616	278 (0.45)	33 (12.3, 18-84)	-	614	1	-	1	616	-	-	-	-	-
PCGC_CHD	1905	1020 (0.54)	28 (17.5, 0-72)	1371	101	272	114	47	-	1905	-	-	-	-
PharmHU	173	87 (0.5)	8 (5.7, 0-21)	-	154	16	2	1	173	-	-	-	-	-
PIMA	50	17 (0.34)	10 (3.2, 6-17)	1	2	47	-	-	-	-	-	-	50	-
PUSH_SCD	364	184 (0.51)	12 (5.2, 3-20)	2	356	5	-	1	364	-	-	-	-	-
REDS-III_Brazil	2610	1227 (0.47)	20 (13.9, 0-77)	-	-	-	-	2610	2610	-	-	-	-	-
SAFS	996	425 (0.43)	37 (15.1, 16-84)	19	3	971	-	3	-	996	-	-	-	-
SAGE	1838	839 (0.46)	17 (6.7, 7-41)	1	1831	2	1	3	-	-	-	443	1395	-
SAPPHIRE_asthma	4688	1649 (0.35)	34 (14, 12-64)	220	4284	112	52	20	-	-	-	-	4688	-
SARP	1748	670 (0.38)	36 (17.4, 6-84)	1059	594	44	42	9	-	-	-	1748	-	-
Samoan	1284	510 (0.4)	45 (11.3, 25-65)	-	-	-	-	1284	-	-	-	906	378	-
THRV	2144	1056 (0.49)	52 (10, 17-86)	-	-	-	2144	-	2144	-	-	-	-	-
VAFAR	117	76 (0.65)	59 (8.5, 26-75)	117	-	-	-	-	-	117	-	-	-	-
VU_AF	1106	807 (0.73)	53 (11.2, 16-81)	1044	48	8	4	2	-	1106	-	-	-	-
walk_PHaSST	415	193 (0.47)	37 (13.5, 12-84)	1	397	16	-	1	415	-	-	-	-	-
WGHS	115	0 (0)	49 (3.4, 45-59)	114	-	-	-	1	-	115	-	-	-	-
WHI	10989	1 (0)	69 (6.9, 50-87)	9027	1443	277	203	39	-	10989	-	-	-	-
**Total**	**109122**	**47604 (0.44)**	**49 (20.4, 0-98)**	**51654**	**29260**	**18019**	**5683**	**4506**	**34377**	**36350**	**1383**	**6253**	**25515**	**5244**

Table S2, Conditional analysis results by chromosome: Results of the iterative conditional analysis using individual level data. Results are presented by chromosome providing a detailed overview of the conditional step at which each variant was identified as the peak signal. (Note that some variants had p-value>5x10^{-9} in the primary analysis, but p-value<5x10^{-9} in a conditional step). Within each chromosome, variants are ordered not on position, but in the sequence of identification through the conditional analysis showing the iterative process used: Primary and Rounds 1-6 of conditioning. Chromosomes varied in the number of analyses needed until no additional variants were included (maximum steps = 7 on chromosome 5). Related to ***Table 1*** and ***Figure 2***.

					LD with Primary		Primary		Conditional Round 1		Conditional Round 2		Conditional Round 3		Conditional Round 4		Conditional Round 5		Conditional Round 6	
chr	pos	Locus	rsNum	Round	r2	D'	Est	p-value	Est	p-value	Est	p-value	Est	p-value	Est	p-value	Est	p-value	Est	p-value
1	226367601	PARP1	rs1136410	Primary			**-28.50**	**9.32E-20**												
1	35259602	ZMYM4	rs11581846	Cond_1	0.001	0.06	-19.09	3.04E-10	**-19.14**	**2.74E-10**										
1	113881455	BCL2L15	rs2296176	Cond_2	0.000	0.02	-19.70	2.84E-10	-19.58	3.58E-10	**-19.56**	**3.69E-10**								
2	54263623	ACYP2	rs144980386	Primary			**28.23**	**1.32E-17**												
2	54255416	ACYP2	rs17189743	Cond_1	0.003	1.00	-58.93	7.18E-12	**-55.56**	**1.06E-10**										
3	169769649	TERC	rs12637184	Primary			**-59.07**	**1.30E-96**												
3	169772313	TERC	rs9826466	Cond_1	0.005	1.00	-70.30	3.25E-17	**-75.18**	**1.70E-19**										
3	117584223	LINC00901	rs961617801	Cond_2	0.000	1.00	1063.26	1.25E-11	1050.17	2.02E-11	**1049.68**	**2.05E-11**								
3	190053412	P3H2	rs10937417	Cond_3	0.008	0.22	14.49	6.89E-10	14.13	1.66E-09	14.23	1.26E-09	**14.19**	**1.39E-09**						
4	163155406	NAF1	rs1351222	Primary			**28.15**	**3.27E-26**												
4	163144568	NAF1	rs113580095	Cond_1	0.004	1.00	-273.46	4.72E-18	**-251.28**	**1.96E-15**										
4	163126692	NAF1	rs60735607	Cond_2	0.106	1.00	-7.44	0.00396382	-18.51	1.28E-11	**-18.43**	**1.55E-11**								
4	9928595	SLC2A2	rs4235345	Cond_3	0.000	0.01	16.86	3.82E-09	16.79	4.30E-09	16.77	4.47E-09	**16.92**	**3.22E-09**						
5	1285859	TERT	rs7705526	Primary			**50.87**	**1.64E-92**												
5	1292331	TERT	rs34052286	Cond_1	0.005	0.27	-48.46	1.50E-12	**-70.34**	**2.75E-24**										
5	1287079	TERT	rs2853677	Cond_2	0.257	0.60	-39.84	8.17E-65	-21.47	1.31E-15	**-22.91**	**1.54E-17**								
5	1272383	TERT	rs192999400	Cond_3	0.004	0.97	82.11	3.10E-15	91.36	1.72E-18	87.95	2.94E-17	**89.49**	**8.00E-18**						
5	1292843	TERT	rs114616103	Cond_4	0.000	0.06	-40.67	2.39E-07	-42.83	5.06E-08	-44.51	1.47E-08	-47.07	2.14E-09	**-57.82**	**3.25E-13**				
5	1280823	TERT	rs6897196	Cond_5	0.316	0.82	44.48	1.87E-83	25.00	1.15E-17	21.30	4.98E-13	20.77	1.83E-12	20.81	1.61E-12	**21.49**	**3.07E-13**		
5	139637905	CXXC5	rs75903170	Cond_6	0.001	0.09	30.40	5.69E-10	30.19	6.88E-10	29.87	1.03E-09	29.64	1.36E-09	29.60	1.41E-09	29.48	1.63E-09	**29.42**	**1.76E-09**
6	31815431	HSPA1A	rs1008438	Primary			**-19.75**	**3.42E-17**												
7	124812616	POT1	rs720613	Primary			**-26.40**	**1.27E-26**												
7	129041243	TNP03	rs7783384	Cond_1	0.000	0.01	-15.89	2.34E-12	**-15.89**	**2.23E-12**										
7	124858989	POT1	rs202187871	Cond_2	0.000	0.96	727.22	4.89E-12	726.39	4.98E-12	**727.04**	**4.70E-12**								
8	73046483	TERF1	rs10112752	Primary			**-17.10**	**4.59E-13**												
8	73033303	TERF1	rs73687065	Cond_1	0.004	1.00	88.85	3.10E-12	**83.89**	**4.90E-11**										
8	73004218	TERF1	rs183633026	Cond_2	0.003	0.99	101.90	1.59E-10	97.09	1.13E-09	**97.77**	**8.66E-10**								
10	103916707	OBFC1	rs9420907	Primary			**-54.72**	**3.90E-83**												
10	103918153	OBFC1	rs111447985	Cond_1	0.004	1.00	109.76	2.29E-24	**116.56**	**2.72E-27**										
10	94344908	NOC3L	rs3758526	Cond_2	0.001	0.05	-21.48	6.80E-12	-22.01	1.88E-12	**-21.79**	**3.06E-12**								
10	99514276	NKX2-3	rs10883359	Cond_3	0.021	0.43	-18.14	3.60E-12	-16.71	1.46E-10	-17.12	4.98E-11	**-17.29**	**3.23E-11**						
10	103915847	OBFC1	rs112163720	Cond_4	0.026	1.00	3.35	0.44000458	24.06	6.29E-08	26.92	1.47E-09	27.21	9.69E-10	**26.89**	**1.50E-09**				
10	103907794	OBFC1	rs10883948	Cond_5	0.246	0.98	-27.96	2.04E-34	-8.30	0.0015196	-12.20	3.81E-06	-12.10	4.55E-06	-12.29	3.20E-06	**-18.90**	**8.80E-12**		
11	108158382	ATM	rs61380955	Primary			-19.29	**2.47E-17**												
13	41150640	KBTBD7	rs1411041	Primary			22.08	**6.29E-14**												
14	24242592	TINF2	rs28372734	Primary			108.81	**1.74E-27**												
14	72959582	DCAF4	rs2572	Cond_1	0.004	0.18	26.37	5.14E-12	**26.29**	**5.67E-12**										
14	24243052	TINF2	rs8016076	Cond_2	0.000	1.00	79.49	1.80E-11	80.64	9.06E-12	**80.60**	**9.23E-12**								
14	24254544	TINF2	rs41293824	Cond_3	0.000	1.00	80.93	1.31E-09	82.24	6.96E-10	82.48	6.19E-10	**84.54**	**2.26E-10**						
15	50065546	ATP8B4	rs7172615	Primary			-17.00	**4.31E-09**												
16	82166498	MPHOSPH6	rs2967355	Primary			-27.86	**3.96E-19**												
16	74630845	RFWD3	rs7193541	Cond_1	0.001	0.07	-18.69	1.47E-16	**-18.71**	**1.34E-16**										
16	69357811	TERF2	rs9925619	Cond_2	0.002	0.14	18.64	3.01E-14	18.61	3.24E-14	**18.92**	**1.21E-14**								
16	70193527	CLEC18C	rs62049363	Cond_3	0.010	0.14	-16.23	4.09E-10	-16.38	2.73E-10	-16.62	1.48E-10	**-16.65**	**1.36E-10**						
16	87961594	BANP	rs12934497	Cond_4	0.004	0.11	14.93	9.15E-10	14.86	1.06E-09	14.75	1.42E-09	14.88	9.96E-10	**14.83**	**1.12E-09**				
18	666625	TYMS	rs8088781	Primary			**-27.73**	**2.49E-15**												
18	676473	TYMS	rs2612101	Cond_1	0.357	0.96	2.15	0.40769627	**22.60**	**3.37E-12**										
18	44666476	SETBP1	rs2852770	Cond_2	0.000	0.01	-18.59	1.00E-11	-18.56	1.06E-11	**-18.58**	**9.99E-12**								
18	650764	TYMS	rs150119891	Cond_3	0.000	0.60	-76.76	2.27E-07	-80.32	6.13E-08	-100.93	1.98E-11	**-100.93**	**1.96E-11**						
19	22032639	ZNF257_ZNF676	rs8105767	Primary			20.57	3.59E-18												
20	63678201	RTEL1	rs41309367	Primary			**-29.14**	**2.52E-33**												
20	63689775	RTEL1	rs35640778	Cond_1	0.022	0.99	-120.85	2.31E-28	**-144.36**	**6.39E-39**										
20	36922795	SAMHD1	rs2342113	Cond_2	0.003	0.06	-23.27	4.62E-18	-23.26	4.60E-18	**-23.25**	**4.52E-18**								
20	63695521	RTEL1	rs181080831	Cond_3	0.000	0.47	174.01	6.40E-17	177.46	1.49E-17	176.23	2.36E-17	**177.20**	**1.55E-17**						
20	63661765	RTEL1	rs41308088	Cond_4	0.033	0.99	29.50	1.58E-10	40.34	6.80E-18	39.84	1.64E-17	39.69	2.09E-17	**40.36**	**5.88E-18**				
20	63676585	RTEL1	rs79981941	Cond_5	0.044	0.91	-37.56	1.46E-23	-29.82	6.54E-15	-29.91	5.22E-15	-29.97	4.45E-15	-29.46	1.26E-14	**-25.64**	**2.82E-11**		
22	50532618	TYMP	rs361725	Primary			**-14.99**	**1.36E-10**												
X	66015290	VSIG4	rs12394264	Primary			**18.62**	**9.67E-16**												
X	154720412	GAB3	rs2728723	Cond_1	0.009	0.11	13.80	1.21E-12	**13.76**	**1.38E-12**										

Table S3, Results at prior GWAS variants: TOPMed results from the primary pooled trans-population analysis for all prior telomere length GWAS sentinel variants with reported p-value $<5x10^{-8}$ in prior published studies. Related to ***STAR Methods*** **.**

					Previously reported results							Single variant anlaysis in pooled trans-population sample		
Chromosome	Position (hg38)	rsID	Ref	Alt	Locus Name	First Author	Year	PMCID	P-Value	Direction	Near Table 1 Variant	P-value	Estimate (bp)	AAF
1	226374920	rs3219104	A	C	PARP1	Dorajoo	2019	PMC6554354	2.23E-16	+	Yes	1.27E-18	25.6	80.9%
1	226374920	rs3219104	A	C	PARP1	Li	2020	PMC7058826	9.31E-11	+	Yes	1.27E-18	25.6	80.9%
2	54248729	rs11125529	C	A	ACYP2	Codd	2013	PMC4006270	4.48E-08	+	Yes	8.82E-16	25.5	14.6%
2	136258421	rs4452212	G	A	CXCR4	Levy	2010	PMC2889047	2.94E-08	N/A	No	2.19E-01	-3.4	32.7%
3	58390292	rs6772228	T	A	PXK	Pooley	2013	PMC3836481	3.91E-10	N/A	No	6.38E-01	-3.2	3.0%
3	101513249	rs55749605	C	A	SENP7	Li	2020	PMC7058826	2.38E-08	-	No	2.26E-02	-5.6	69.7%
3	169759718	rs12638862	A	G	TERC	Delgado	2017	PMC5749304	2.20E-08	-	Yes	2.24E-65	-44.5	25.8%
3	169763483	rs12696304	C	G	TERC	Prescott	2011	PMC3091863	1.50E-14	N/A	Yes	4.18E-44	-33.2	39.9%
3	169764547	rs2293607	T	C	TERC	Dorajoo	2019	PMC6554354	7.57E-39	-	Yes	3.19E-96	-58.9	21.7%
3	169774313	rs10936599	C	T	TERC	Codd	2013	PMC4006270	2.54E-31	-	Yes	8.15E-95	-58.5	21.8%
3	169779797	rs1317082	A	G	TERC	Mangino	2012	PMC3510758	1.14E-08	N/A	Yes	1.61E-95	-58.8	21.8%
3	169779797	rs1317082	A	G	TERC	Pooley	2013	PMC3836481	1.33E-19	N/A	Yes	1.61E-95	-58.8	21.8%
3	169796797	rs10936600	A	T	LRRC34 (TERC)	Li	2020	PMC7058826	6.42E-51	-	Yes	3.99E-86	-54.5	22.6%
3	169850328	rs16847897	G	C	TERC	Prescott	2011	PMC3091863	1.60E-13	N/A	Yes	1.54E-38	-31.5	31.9%
3	169862183	rs1920116	G	A	TERC	Walsh	2014	PMC4074274	8.30E-09	-	Yes	2.49E-46	-37.5	26.3%
4	70908630	rs13137667	T	C	MOB1B	Li	2020	PMC7058826	2.37E-08	+	No	5.81E-02	8.1	92.3%
4	107383042	rs7680468	G	T	DKK2_PAPSS1	Lee	2014	PMC3894567	4.70E-08	N/A	No	7.26E-01	2.3	2.8%
4	163086668	rs7675998	A	G	NAF1	Codd	2013	PMC4006270	4.35E-16	+	Yes	5.05E-22	26.3	78.4%
4	163127047	rs4691895	G	C	NAF1	Li	2020	PMC7058826	1.47E-21	+	Yes	3.48E-26	28.5	77.8%
4	163180330	rs10857352	A	G	NAF1	Dorajoo	2019	PMC6554354	4.85E-09	+	Yes	1.48E-21	21.7	45.7%
5	1282204	rs7726159	C	A	TERT	Pooley	2013	PMC3836481	4.67E-17	N/A	Yes	4.04E-87	49.1	29.8%
5	1285859	rs7705526	C	A	TERT	Dorajoo	2019	PMC6554354	2.61E-38	+	Yes	1.64E-92	50.9	29.4%
5	1285859	rs7705526	C	A	TERT	Li	2020	PMC7058826	4.82E-45	+	Yes	1.64E-92	50.9	29.4%
5	1286401	rs2736100	C	A	TERT	Codd	2013	PMC4006270	4.38E-19	-	Yes	5.27E-79	-42.3	52.3%
5	1286401	rs2736100	C	A	TERT	Walsh	2014	PMC4074274	1.40E-15	-	Yes	5.27E-79	-42.3	52.3%
5	1287079	rs2853677	G	A	TERT	Li	2020	PMC7058826	3.12E-31	-	Yes	8.17E-65	-39.8	62.9%
6	25480100	rs34991172	T	G	CARMIL1	Li	2020	PMC7058826	6.03E-09	-	No	2.51E-01	-7.3	3.4%
6	31619784	rs2736176	G	C	PRRC2A	Li	2020	PMC7058826	3.41E-10	+	Yes	2.10E-09	16.3	24.1%
7	124914213	rs59294613	C	A	POT1	Li	2020	PMC7058826	1.12E-13	-	Yes	5.59E-25	-25.7	28.0%
7	124959695	rs7776744	A	G	POT1	Dorajoo	2019	PMC6554354	2.51E-10	-	Yes	4.28E-13	-16.3	53.8%
8	73008648	rs28365964	T	C	TERF1	Dorajoo	2019	PMC6554354	6.96E-15	+	Yes	1.46E-02	76.9	0.1%
10	103900068	rs2487999	T	C	OBFC1	Pooley	2013	PMC3836481	4.22E-14	N/A	Yes	3.14E-53	-49.6	84.4%
10	103916188	rs9419958	T	C	STN1 (OBFC1)	Li	2020	PMC7058826	4.77E-19	-	Yes	9.05E-83	-54.6	74.9%
10	103916188	rs9419958	T	C	OBFC1	Mangino	2012	PMC3510758	9.13E-11	N/A	Yes	9.05E-83	-54.6	74.9%
10	103916707	rs9420907	C	A	OBFC1	Codd	2013	PMC4006270	6.90E-11	-	Yes	3.90E-83	-54.7	74.9%
10	103918139	rs4387287	A	C	OBFC1	Levy	2010	PMC2889047	3.87E-09	N/A	Yes	2.35E-77	-49.7	67.8%
10	103920828	rs12415148	T	C	OBFC1	Dorajoo	2019	PMC6554354	2.78E-25	+	Yes	1.04E-23	107.5	1.1%
11	108234866	rs228595	G	A	ATM	Li	2020	PMC7058826	1.39E-08	-	Yes	2.12E-13	-17.7	36.2%
11	108377161	rs227080	A	G	ATM	Dorajoo	2019	PMC6554354	1.87E-10	-	Yes	1.27E-14	-17.5	53.7%
14	24252121	rs41293836	C	T	TINF2	Dorajoo	2019	PMC6554354	2.47E-42	+	Yes	6.49E-08	47.6	1.6%
14	72938044	rs2302588	G	C	DCAF4	Li	2020	PMC7058826	1.64E-08	+	Yes	5.76E-10	23.4	9.7%
14	72948525	rs2535913	G	A	DCAF4	Mangino	2015	PMC4345921	6.38E-10	N/A	Yes	1.77E-09	-15.8	24.7%
16	58175370	rs74019828	G	A	CSNK2A2	Saxena	2014	PMC4106467	4.52E-08	-	No	3.62E-01	-4.2	6.3%
16	69373083	rs3785074	A	G	TERF2	Li	2020	PMC7058826	4.50E-10	+	Yes	4.73E-14	18.4	30.8%
16	74646176	rs62053580	A	G	RFWD3	Li	2020	PMC7058826	3.96E-08	-	Yes	3.67E-11	-22.1	13.2%
16	82166375	rs7194734	C	T	MPHOSPH6	Li	2020	PMC7058826	6.72E-10	-	Yes	3.31E-13	-20.8	80.9%
17	8232774	rs3027234	C	T	CTC1	Mangino	2012	PMC3510758	2.29E-08	N/A	No	7.97E-05	-12.6	15.2%
19	22032639	rs8105767	A	G	ZNF208	Codd	2013	PMC4006270	1.11E-09	+	Yes	3.59E-18	20.6	35.1%
19	22032639	rs8105767	A	G	ZNF208	Li	2020	PMC7058826	5.21E-13	+	Yes	3.59E-18	20.6	35.1%
19	22176638	rs412658	C	T	ZNF676	Mangino	2012	PMC3510758	9.75E-09	N/A	Yes	5.15E-07	11.6	42.7%
20	63638397	rs75691080	C	T	RTEL1/STMN3	Li	2020	PMC7058826	5.75E-14	-	Yes	6.92E-17	-33.5	8.5%
20	63660246	rs34978822	C	G	RTEL1	Li	2020	PMC7058826	7.04E-10	-	Yes	1.78E-28	-122.5	1.1%
20	63678201	rs41309367	C	T	RTEL1	Dorajoo	2019	PMC6554354	1.16E-08	-	Yes	2.52E-33	-29.1	67.3%
20	63678486	rs6010620	A	G	RTEL1	Walsh	2014	PMC4074274	4.70E-19	-	Yes	3.13E-10	-18.5	79.1%
20	63790269	rs755017	A	G	RTEL1	Codd	2013	PMC4006270	6.71E-09	+	Yes	3.58E-05	11.9	19.6%
20	63805045	rs73624724	T	C	RTEL1/ZBTB46	Li	2020	PMC7058826	6.08E-12	+	Yes	1.65E-04	10.9	19.3%

Table S4, Association results for the single variant association analysis for each of the 59 sentinel variants from *Table 1* : Single variant results are shown for the pooled trans-population analysis, and each population group. Variants with a minor allele count <5 were not included in the analysis and are listed as "-". Related to ***Table 1*** **.**

				Est (in bp) from the single variant analysis by stratum					P-value from the single variant analysis by stratum					Alternate allele frequency					Percent variation explained				
Chr	Position	Locus	rsNum	Trans-population	European	African	Hispanic /Latino	Asian	Trans-population	European	African	Hispanic /Latino	Asian	Trans-population	European	African	Hispanic /Latino	Asian	Trans-population	European	African	Hispanic /Latino	Asian
1	35259602	ZMYM4	rs11581846	-19.1	-21.6	-11.8	-25.4	-6.1	3.04E-10	7.72E-05	4.31E-02	3.18E-06	5.73E-01	59.9%	89.6%	19.4%	57.8%	23.4%	0.036%	0.030%	0.014%	0.121%	0.006%
1	113881455	BCL2L15	rs2296176	-19.7	-22.1	-22.9	-9.5	-23.9	2.84E-10	1.82E-07	1.93E-03	2.06E-01	4.38E-02	15.2%	19.1%	9.2%	13.9%	16.0%	0.036%	0.053%	0.033%	0.009%	0.072%
1	226367601	PARP1	rs1136410	-28.5	-27.0	-19.5	-26.3	-32.2	9.32E-20	3.52E-09	3.79E-02	1.84E-05	3.11E-04	16.7%	15.7%	5.5%	27.6%	41.7%	0.076%	0.068%	0.015%	0.102%	0.230%
2	54255416	ACYP2	rs17189743	-58.9	-57.5	-28.7	-82.6	-37.8	7.18E-12	1.84E-07	2.89E-01	9.38E-06	3.04E-01	1.7%	2.3%	0.6%	1.9%	1.4%	0.043%	0.053%	0.004%	0.109%	0.019%
2	54263623	ACYP2	rs144980386	28.2	33.7	21.5	30.2	5.9	1.32E-17	1.92E-12	7.80E-04	2.50E-04	6.15E-01	13.2%	14.1%	12.8%	11.2%	16.9%	0.067%	0.096%	0.039%	0.075%	0.004%
3	117584223	LINC00901	rs961617801	1063.3	1082.0	-	-	-	1.25E-11	1.33E-10	-	-	-	0.01%	0.01%	-	-	-	0.042%	0.080%	-	-	-
3	169769649	TERC	rs12637184	-59.1	-56.6	-62.4	-65.8	-55.1	1.30E-96	6.32E-48	1.35E-13	4.36E-28	2.94E-10	21.7%	24.1%	7.1%	29.5%	52.9%	0.399%	0.410%	0.188%	0.671%	0.702%
3	169772313	TERC	rs9826466	-70.3	307.0	-71.6	-66.2	-	3.25E-17	1.33E-01	9.16E-16	1.38E-02	-	1.9%	0.01%	6.1%	0.9%	-	0.065%	0.004%	0.221%	0.034%	-
3	190053412	P3H2	rs10937417	14.5	12.8	18.1	17.2	-3.7	6.89E-10	2.97E-04	2.03E-05	2.85E-03	6.91E-01	36.8%	33.3%	49.1%	29.4%	31.2%	0.035%	0.025%	0.062%	0.050%	0.003%
4	9928595	SLC2A2	rs4235345	16.9	19.0	12.4	15.1	48.5	3.82E-09	1.64E-06	5.66E-02	8.70E-03	5.44E-02	20.3%	23.0%	12.5%	31.5%	3.5%	0.032%	0.045%	0.012%	0.038%	0.065%
4	163126692	NAF1	rs60735607	-7.4	-9.0	-2.2	-10.9	6.6	3.96E-03	1.97E-02	6.23E-01	8.81E-02	7.32E-01	26.3%	24.8%	36.3%	21.1%	5.5%	0.008%	0.011%	0.001%	0.016%	0.002%
4	163144568	NAF1	rs113580095	-273.5	-254.3	-331.8	-278.3	-	4.72E-18	2.07E-09	4.09E-02	7.66E-08	-	0.1%	0.2%	0.02%	0.3%	-	0.069%	0.070%	0.014%	0.161%	-
4	163155406	NAF1	rs1351222	28.2	28.5	23.5	36.7	30.8	3.27E-26	4.56E-13	4.04E-06	3.11E-09	4.18E-03	77.0%	76.4%	77.3%	77.5%	79.0%	0.103%	0.102%	0.073%	0.195%	0.145%
5	1272383	TERT	rs192999400	82.1	81.7	81.0	75.7	95.6	3.10E-15	2.64E-01	8.22E-13	2.41E-02	1.05E-01	1.1%	0.0%	3.6%	0.6%	0.5%	0.057%	0.002%	0.175%	0.028%	0.046%
5	1280823	TERT	rs6897196	44.5	43.3	38.7	44.9	65.0	1.87E-83	2.47E-37	2.80E-18	1.27E-16	2.45E-13	46.8%	40.1%	63.0%	38.6%	39.4%	0.344%	0.316%	0.260%	0.381%	0.948%
5	1285859	TERT	rs7705526	50.9	52.8	35.0	53.0	67.0	1.64E-92	1.29E-50	1.65E-10	5.22E-20	3.65E-14	29.4%	33.5%	18.8%	28.5%	40.8%	0.382%	0.434%	0.140%	0.467%	1.014%
5	1287079	TERT	rs2853677	-39.8	-33.3	-38.7	-47.6	-61.8	8.17E-65	2.76E-23	8.35E-16	1.59E-17	3.10E-12	62.9%	56.9%	72.7%	67.0%	60.6%	0.265%	0.191%	0.222%	0.404%	0.860%
5	1292331	TERT	rs34052286	-48.5	-60.1	-45.5	-51.2	-	1.50E-12	3.90E-01	2.26E-09	4.33E-03	-	2.8%	0.1%	8.6%	2.1%	-	0.046%	0.001%	0.122%	0.045%	-
5	1292843	TERT	rs114616103	-40.7	-47.9	-17.3	-44.3	-28.1	2.39E-07	1.30E-06	2.79E-01	7.08E-02	7.93E-01	2.1%	2.9%	1.8%	1.1%	0.2%	0.024%	0.045%	0.004%	0.018%	0.001%
5	139637905	CXXC5	rs75903170	30.4	23.5	48.6	27.4	13.9	5.69E-10	1.02E-03	1.56E-06	3.68E-02	3.92E-01	5.5%	5.6%	4.6%	4.0%	7.7%	0.035%	0.021%	0.079%	0.024%	0.013%
6	31815431	HSPA1A	rs1008438	-19.8	-18.6	-18.5	-25.6	-17.8	3.42E-17	5.22E-08	1.68E-04	8.89E-07	4.23E-02	51.0%	38.0%	73.9%	52.2%	44.4%	0.065%	0.057%	0.048%	0.134%	0.073%
7	124812616	POT1	rs720613	-26.4	-31.9	-19.4	-26.8	-19.3	1.27E-26	3.17E-18	2.54E-05	1.17E-05	4.24E-02	28.6%	29.1%	30.9%	24.0%	28.6%	0.105%	0.147%	0.061%	0.107%	0.073%
7	124858989	POT1	rs202187871	727.2	710.0	-	-	-	4.89E-12	3.61E-11	-	-	-	0.01%	0.03%	-	-	-	0.044%	0.085%	-	-	-
7	129041243	TNP03	rs7783384	-15.9	-18.3	-14.0	-13.3	-19.7	2.34E-12	1.03E-07	9.75E-04	1.07E-02	2.39E-02	57.6%	62.6%	53.5%	54.0%	50.9%	0.045%	0.055%	0.037%	0.036%	0.090%
8	73004218	TERF1	rs183633026	101.9	73.1	-7.0	112.0	-	1.59E-10	4.49E-02	9.26E-01	1.85E-09	-	0.5%	0.2%	0.1%	2.2%	-	0.038%	0.008%	0.000%	0.201%	-
8	73033303	TERF1	rs73687065	88.9	29.8	93.0	79.4	-	3.10E-12	7.37E-01	3.73E-11	2.79E-02	-	0.8%	0.03%	2.3%	0.5%	-	0.045%	0.000%	0.150%	0.027%	-
8	73046483	TERF1	rs10112752	-17.1	-18.7	-14.8	-16.4	-26.9	4.59E-13	2.35E-08	1.31E-03	3.77E-03	2.67E-02	36.2%	43.7%	30.8%	32.0%	15.6%	0.048%	0.060%	0.035%	0.047%	0.087%
10	94344908	NOC3L	rs3758526	-21.5	-20.2	-21.9	-16.8	-26.5	6.80E-12	5.83E-05	6.94E-05	4.16E-02	5.88E-03	14.9%	12.5%	18.2%	11.1%	28.1%	0.043%	0.031%	0.054%	0.023%	0.134%
10	99514276	NKX2-3	rs10883359	-18.1	-14.9	-34.5	-12.7	-13.5	3.60E-12	5.15E-05	3.75E-08	2.73E-02	1.27E-01	25.2%	28.6%	13.3%	29.5%	41.3%	0.044%	0.032%	0.104%	0.027%	0.041%
10	103907794	OBFC1	rs10883948	-28.0	-25.8	-49.0	-14.8	3.5	2.04E-34	8.05E-15	8.43E-27	4.61E-03	7.09E-01	43.3%	50.1%	31.7%	46.9%	33.1%	0.137%	0.117%	0.393%	0.045%	0.002%
10	103915847	OBFC1	rs112163720	3.3	32.9	-12.8	8.8	10.7	4.40E-01	8.78E-04	5.36E-02	3.60E-01	3.77E-01	7.1%	2.9%	11.6%	8.2%	14.9%	0.001%	0.021%	0.013%	0.005%	0.014%
10	103916707	OBFC1	rs9420907	-54.7	-51.3	-59.3	-57.1	-0.1	3.90E-83	8.24E-27	4.41E-43	1.13E-16	9.98E-01	74.9%	85.9%	47.8%	81.8%	98.0%	0.342%	0.223%	0.648%	0.382%	0.000%
10	103918153	OBFC1	rs111447985	109.8	97.3	48.9	127.9	94.1	2.29E-24	2.65E-02	2.63E-01	9.14E-18	5.30E-07	1.1%	0.1%	0.2%	3.5%	5.9%	0.095%	0.010%	0.004%	0.410%	0.445%
11	108158382	ATM	rs61380955	-19.3	-23.9	-15.5	-16.6	-14.7	2.47E-17	1.45E-12	4.16E-04	1.88E-03	9.65E-02	51.4%	57.5%	38.1%	59.6%	46.7%	0.066%	0.097%	0.043%	0.054%	0.049%
13	41150640	KBTBD7	rs1411041	22.1	24.9	21.3	21.7	17.4	6.29E-14	6.16E-08	4.87E-04	6.57E-04	5.16E-02	60.8%	84.5%	17.1%	75.7%	39.3%	0.052%	0.057%	0.042%	0.065%	0.067%
14	24242592	TINF2	rs28372734	108.8	117.6	95.7	126.7	94.5	1.74E-27	6.07E-02	2.75E-05	4.51E-08	1.75E-09	1.2%	0.1%	0.9%	1.3%	8.2%	0.108%	0.007%	0.060%	0.166%	0.641%
14	24243052	TINF2	rs8016076	79.5	429.9	76.9	83.0	-	1.80E-11	6.51E-02	2.79E-09	1.25E-02	-	0.9%	0.0%	2.8%	0.6%	-	0.041%	0.007%	0.121%	0.035%	-
14	24254544	TINF2	rs41293824	80.9	60.7	73.4	124.4	-	1.31E-09	6.36E-01	9.37E-07	4.55E-04	-	0.7%	0.0%	2.1%	0.5%	-	0.034%	0.000%	0.082%	0.068%	-
14	72959582	DCAF4	rs2572	26.4	26.6	35.2	21.0	33.6	5.14E-12	1.04E-06	2.50E-04	1.89E-02	2.27E-03	9.5%	10.3%	5.2%	8.9%	19.5%	0.044%	0.046%	0.046%	0.031%	0.165%
15	50065546	ATP8B4	rs7172615	-17.0	-19.7	-21.1	-7.2	-11.2	4.31E-09	4.64E-07	5.19E-03	3.00E-01	2.30E-01	18.8%	23.3%	8.8%	16.8%	31.7%	0.032%	0.049%	0.027%	0.006%	0.025%
16	69357811	TERF2	rs9925619	18.6	14.4	22.2	25.9	7.4	3.01E-14	1.10E-04	2.63E-07	1.04E-05	5.88E-01	30.3%	27.5%	40.9%	26.6%	12.0%	0.053%	0.029%	0.091%	0.108%	0.005%
16	70193527	CLEC18C	rs62049363	-16.2	-16.3	-11.3	-17.9	-10.7	4.09E-10	1.68E-06	1.49E-01	1.78E-03	2.48E-01	28.3%	39.2%	8.6%	30.6%	30.5%	0.036%	0.044%	0.007%	0.054%	0.024%
16	74630845	RFWD3	rs7193541	-18.7	-23.5	-17.6	-13.3	-5.6	1.47E-16	2.57E-12	3.38E-05	1.60E-02	5.25E-01	42.6%	41.6%	50.1%	34.1%	41.5%	0.063%	0.095%	0.059%	0.032%	0.007%
16	82166498	MPHOSPH6	rs2967355	-27.9	-25.6	-32.2	-37.7	1.0	3.96E-19	2.00E-10	4.19E-04	5.10E-07	9.33E-01	84.0%	78.0%	94.1%	85.8%	83.1%	0.073%	0.078%	0.043%	0.140%	0.000%
16	87961594	BANP	rs12934497	14.9	13.8	16.1	16.8	34.3	9.15E-10	3.57E-05	1.06E-03	2.97E-03	9.59E-02	35.3%	46.5%	25.4%	31.8%	4.8%	0.034%	0.033%	0.037%	0.049%	0.049%
18	650764	TYMS	rs150119891	-76.8	-77.6	-41.2	-138.4	-	2.27E-07	2.12E-06	4.03E-01	1.27E-02	-	0.6%	1.1%	0.2%	0.2%	-	0.025%	0.044%	0.002%	0.035%	-
18	666625	TYMS	rs8088781	-27.7	-28.3	-27.2	-28.0	-4.9	2.49E-15	2.46E-09	3.51E-05	2.54E-03	9.21E-01	11.8%	14.4%	11.9%	8.4%	0.8%	0.057%	0.069%	0.059%	0.051%	0.000%
18	676473	TYMS	rs2612101	2.2	-8.5	5.5	18.5	45.9	4.08E-01	3.07E-02	2.20E-01	1.44E-03	2.88E-01	25.7%	23.3%	34.2%	27.9%	1.1%	0.001%	0.009%	0.005%	0.056%	0.020%
18	44666476	SETBP1	rs2852770	-18.6	-25.9	-7.8	-24.0	-6.0	1.00E-11	1.60E-09	1.01E-01	2.25E-04	5.79E-01	21.3%	18.6%	27.0%	20.3%	20.0%	0.042%	0.071%	0.009%	0.076%	0.005%
19	22032639	ZNF257_ZNF	rs8105767	20.6	21.6	23.3	12.7	26.7	3.59E-18	3.63E-09	4.85E-08	2.15E-02	4.03E-03	35.1%	29.1%	47.1%	34.6%	32.2%	0.069%	0.067%	0.102%	0.029%	0.146%
20	36922795	SAMHD1	rs2342113	-23.3	-33.8	-15.3	-26.1	-7.9	4.62E-18	7.52E-13	9.76E-04	7.99E-06	3.66E-01	23.7%	14.5%	30.0%	30.8%	46.9%	0.069%	0.100%	0.037%	0.111%	0.014%
20	63661765	RTEL1	rs41308088	29.5	36.2	14.0	16.6	59.7	1.58E-10	2.75E-10	3.04E-01	1.00E-01	1.92E-01	6.5%	9.2%	2.5%	7.3%	1.1%	0.038%	0.077%	0.004%	0.015%	0.030%
20	63676585	RTEL1	rs79981941	-37.6	-40.3	-28.4	-47.7	-51.3	1.46E-23	1.65E-12	1.43E-05	2.80E-08	6.40E-02	9.9%	9.3%	12.0%	10.1%	2.9%	0.092%	0.097%	0.064%	0.171%	0.061%
20	63678201	RTEL1	rs41309367	-29.1	-26.2	-37.8	-34.0	-7.0	2.52E-33	2.55E-13	8.79E-16	1.67E-09	4.73E-01	67.3%	68.8%	70.7%	69.1%	30.1%	0.133%	0.104%	0.221%	0.202%	0.009%
20	63689775	RTEL1	rs35640778	-120.9	-121.4	-143.7	-95.6	5.2	2.31E-28	1.00E-21	3.07E-05	1.90E-03	9.72E-01	1.1%	1.7%	0.4%	0.7%	0.1%	0.112%	0.178%	0.059%	0.054%	0.000%
20	63695521	RTEL1	rs181080831	174.0	190.3	12.5	142.0	-	6.40E-17	9.02E-16	8.81E-01	1.07E-02	-	0.3%	0.5%	0.1%	0.2%	-	0.064%	0.125%	0.000%	0.036%	-
22	50532618	TYMP	rs361725	-15.0	-21.2	-5.0	-11.3	-20.1	1.36E-10	5.91E-10	2.86E-01	3.31E-02	3.55E-02	53.4%	63.0%	30.9%	59.0%	69.8%	0.038%	0.074%	0.004%	0.025%	0.078%
X	66015290	VSIG4	rs12394264	18.6	18.5	15.3	22.2	7.7	9.67E-16	7.46E-07	4.58E-05	1.76E-05	8.44E-01	29.8%	16.0%	62.3%	23.8%	0.9%	0.059%	0.047%	0.057%	0.102%	0.001%
X	154720412	GAB3	rs2728723	13.8	16.5	7.1	14.4	16.6	1.21E-12	1.86E-08	4.96E-02	1.10E-03	5.50E-02	62.8%	67.9%	52.2%	60.0%	79.8%	0.046%	0.061%	0.013%	0.059%	0.065%

Table S5, Iterative conditional analysis of *OBFC1* : Iterative conditional analysis was repeated on chromosome 10 focusing exclusively on the *OBFC1* locus, defined as a 2Mb window around the original top sentinel, rs9420907. The sentinel for each signal was consistent with the iterative conditional analysis performed on the entirety of chromosome 10 (***Table S2***). The summary statistics from each iterative conditional analysis were used to perform colocalization analysis on the non-primary signals. Colocalization analysis was performed using coloc with all significant gene-tissue pairs in GTEx and with all genes in a 2Mb window around rs9420907 in eQTLGen. All results with PPH4 > 0.7 for each signal are reported. Related to ***Figure 3*** **.**

chr	position	rsNum	Round	r2	D'	Primary		Conditional Round 1		Conditional Round 2		Conditional Round 3		coloc results with GTEx (PPH4 > 0.7)				
						Est	p-value	Est	p-value	Est	p-value	Est	p-value	Gene	Gene Symbol	Tissue	PPH3	PPH4
10	103916707	rs9420907	Primary			**-54.70**	**3.90E-83**							ENSG00000260461.1	RP11-541N10.3	Adipose_Subcutaneous	0.03	0.95
														ENSG00000107960.10	OBFC1	Adipose_Subcutaneous	0.08	0.9
														ENSG00000260461.1	RP11-541N10.3	Colon_Sigmoid	0.13	0.72
														ENSG00000107960.10	OBFC1	Colon_Transverse	0.08	0.92
														ENSG00000107960.10	OBFC1	Esophagus_Mucosa	0.03	0.97
														ENSG00000107960.10	OBFC1	Esophagus_Muscularis	0.05	0.94
														ENSG00000107960.10	OBFC1	Colon_Sigmoid	0.16	0.81
														ENSG00000107960.10	OBFC1	Skin_Not_Sun_Exposed_Suprapubic	0.03	0.97
														ENSG00000107960.10	OBFC1	Skin_Sun_Exposed_Lower_leg	0.02	0.98
														ENSG00000107960.10	OBFC1	Spleen	0.1	0.79
														ENSG00000107960.10	OBFC1	Nerve_Tibial	0.07	0.92
10	103918153	rs111447985	Secondary	0.004	1.00	109.76	2.29E-24	**116.56**	**2.72E-27**					N/A (rare variant, not available in GTEx or eQTLGen)				
10	103915847	rs112163720	Tertiary	0.026	1	3.35	0.440005	24.10	6.29E-08	**26.90**	**1.47E-09**			ENSG00000107960.10	OBFC1	Muscle_Skeletal	0.16	0.84
10	103907794	rs10883948	Quaternary	0.246	0.98	-28.00	2.04E-34	-8.30	1.52E-03	-12.20	3.81E-06	**-18.80**	**1.14E-11**	ENSG00000260461.1	RP11-541N10.3	Brain_Cerebellar_Hemisphere	0.25	0.73
														ENSG00000260461.1	RP11-541N10.3	Brain_Cerebellum	0.26	0.74
														ENSG00000260461.1	RP11-541N10.3	Brain_Cortex	0.24	0.72
														ENSG00000260461.1	RP11-541N10.3	Brain_Frontal_Cortex_BA9	0.25	0.75
														ENSG00000260461.1	RP11-541N10.3	Esophagus_Mucosa	0.23	0.77
														ENSG00000065613.13	SLK	Muscle_Skeletal	0.24	0.72
														ENSG00000065613.13	SLK	Ovary	0.25	0.75
														ENSG00000107960.10	OBFC1	Whole_Blood	0.25	0.75

Table S6, BioVU PheWAS results: Results of the PheWAS performed on 49 available sentinel variants and a polygenic trait score across these 49 sentinel variants using BioVU self-identified African Americans (AA, n=15,174) and BioVU self-identified European Americans (EA, n=70,439). The cumulative TL risk score for the BioVU AA and EA samples was derived from the African and European specific effects sizes from ***Table 1*** , respectively. Results were evaluated at a Bonferroni threshold corrected for the number of informative phecodes for each variant. Related to ***STAR Methods*** **.**

Chr	Pos	Locus	rsNum	Ref	Alt	Trans-population TL Effect size in (bp)	PheWAS Results: Phecode	Category	Phenotype	OR	P-Value	Number of Samples	Ancestry
49 SNP EA PTS							165	neoplasms	Cancer within the respiratory system	1.10	5.76E-06	2465 / 66885	EA
							165.1	neoplasms	Cancer of bronchus; lung	1.11	3.92E-06	2444 / 66885	
							172.1	neoplasms	Melanomas of skin, dx or hx	1.14	1.35E-06	1461 / 59520	
							172.11	neoplasms	Melanomas of skin	1.14	1.93E-05	1209 / 59520	
							189.1	neoplasms	Cancer of kidney and renal pelvis	1.16	4.61E-06	980 / 68020	
							191	neoplasms	Manlignant and unknown neoplasms of brain and nervous system	1.17	1.29E-05	838 / 67856	
							191.1	neoplasms	Cancer of brain and nervous system	1.22	4.62E-07	657 / 67856	
							191.11	neoplasms	Cancer of brain	1.24	2.16E-07	592 / 67856	
							198	neoplasms	Secondary malignant neoplasm	1.07	2.70E-06	6574 / 52740	
							198.1	neoplasms	Secondary malignancy of lymph nodes	1.08	2.63E-05	3381 / 52740	
							199	neoplasms	Neoplasm of uncertain behavior	1.10	1.43E-05	2254 / 52740	
							241	endocrine/metabolic	Nontoxic nodular goiter	1.11	1.45E-05	1909 / 56362	
							241.1	endocrine/metabolic	Nontoxic uninodular goiter	1.16	8.50E-06	925 / 56362	
							571.51	digestive	Cirrhosis of liver without mention of alcohol	0.90	1.20E-06	2062 / 56630	
3	169769649	*TERC*	rs12637184	G	A	-59.79	782.6	symptoms	Pallor and flushing	6.33	2.68E-06	21 / 12880	AA
4	9928595	*SLC2A2*	rs4235345	G	A	16.78	274.1	endocrine/metabolic	Gout	0.65	9.03E-24	2095 / 66779	EA
							274	endocrine/metabolic	Gout and other crystal arthropathies	0.67	9.57E-22	2186 / 66779	EA
							274.11	endocrine/metabolic	Gouty arthropathy	0.61	4.06E-11	685 / 66779	EA
5	1272383	*TERT*	rs192999400	C	T	101.32	573.3	digestive	Hepatomegaly	4.62	2.71E-06	59 / 12759	AA
							742.1	musculoskeletal	Loose body in joint	212.24	3.16E-06	42 / 59012	EA
							772	symptoms	Symptoms of the muscles	37.80	1.01E-05	357 / 61619	EA
							199.4	neoplasms	Neurofibromatosis	179.33	1.10E-05	71 / 52740	EA
							286.13	hematopoietic	Congenital factor VIII disorder	163.41	2.89E-05	80 / 55029	EA
5	1285859	*TERT*	rs7705526	C	A	30.04	200.1	neoplasms	Polycythemia vera	1.72	4.88E-08	214 / 59260	EA
							200	neoplasms	Myeloproliferative disease	1.29	9.32E-08	1006 / 67218	EA
							191.11	neoplasms	Cancer of brain	1.35	9.53E-07	592 / 67856	EA
							191.1	neoplasms	Cancer of brain and nervous system	1.30	5.96E-06	657 / 67856	EA
5	1287079	*TERT*	rs2853677	G	A	-23.89	200.1	neoplasms	Polycythemia vera	0.58	2.06E-08	214 / 59260	EA
5	139637905	*CXXC5*	rs75903170	T	A	29.55	603.2	genitourinary	Spermatocele	3.16	1.31E-05	59 / 61894	EA
6	31815431	*HSPA1A*	rs1008438	A	C	-20.33	250.1	endocrine/metabolic	Type 1 diabetes	1.41	4.11E-28	2192 / 54196	EA
							250.11	endocrine/metabolic	Type 1 diabetes with ketoacidosis	2.05	5.76E-18	300 / 54196	EA
							557.1	digestive	Celiac disease	1.87	1.18E-17	382 / 48299	EA
							250.13	endocrine/metabolic	Type 1 diabetes with ophthalmic manifestations	1.88	2.52E-13	273 / 54196	EA
							250.12	endocrine/metabolic	Type 1 diabetes with renal manifestations	1.80	1.24E-12	300 / 54196	EA
							250.3	endocrine/metabolic	Insulin pump user	1.20	3.04E-12	3108 / 54196	EA
							250	endocrine/metabolic	Diabetes mellitus	1.10	1.88E-09	10334 / 54196	EA
							250.14	endocrine/metabolic	Type 1 diabetes with neurological manifestations	1.44	1.24E-08	505 / 54196	EA
							250.7	endocrine/metabolic	Diabetic retinopathy	1.29	2.65E-07	827 / 63608	EA
							709	dermatologic	Diffuse diseases of connective tissue	1.23	1.16E-06	1202 / 59384	EA
							250.2	endocrine/metabolic	Type 2 diabetes	1.08	4.01E-06	9876 / 54196	EA
							709.2	dermatologic	Sicca syndrome	1.37	1.02E-05	402 / 59384	EA
							244.5	endocrine/metabolic	Congenital hypothyroidism	1.83	1.99E-05	102 / 56362	EA
							250.23	endocrine/metabolic	Type 2 diabetes with ophthalmic manifestations	1.24	2.49E-05	799 / 54196	EA
8	73004218	*TERF1*	rs183633026	A	G	99.36	769	symptoms	Nonallopathic lesions NEC	14.51	1.06E-05	73 / 70207	EA
8	73033303	*TERF1*	rs73687065	T	C	85.32	523.3	digestive	Periodontitis (acute or chronic)	21.07	6.63E-06	214 / 64696	EA
							389.2	sense organs	Conductive hearing loss	3.46	1.85E-05	90 / 14180	AA
10	99514276	*NKX2-3*	rs10883359	A	G	-16.52	555	digestive	Inflammatory bowel disease and other gastroenteritis and colitis	0.86	3.86E-06	2479 / 48299	EA
							555.1	digestive	Regional enteritis	0.85	7.20E-06	2103 / 48299	EA
10	103915847	*OBFC1*	rs112163720	C	T	37.13	634.3	pregnancy complications	Ectopic pregnancy	4.93	1.01E-05	37 / 67408	EA
14	24242592	*TINF2*	rs28372734	C	G	112.59	379.3	sense organs	Aphakia and other disorders of lens	31.99	7.73E-06	91 / 61188	EA
14	24243052	*TINF2*	rs8016076	T	C	83.78	385.5	sense organs	Tympanosclerosis and middle ear disease related to otitis media	423.47	2.52E-06	58 / 61381	EA
							530.3	digestive	Stricture and stenosis of esophagus	3.99	1.53E-05	80 / 10913	AA
							367.9	sense organs	Blindness and low vision	58.59	1.98E-05	452 / 64773	EA
							386.1	sense organs	Meniere's disease	170.74	2.81E-05	213 / 60084	EA
14	24254544	*TINF2*	rs41293824	C	A	83.12	747.2	congenital anomalies	Congenital anomalies of peripheral vascular system	51.72	1.18E-05	203 / 65822	EA
							340	neurological	Migraine	11.94	1.58E-05	2556 / 62491	EA
15	50065546	*ATP8B4*	rs7172615	G	T	-17.75	200	neoplasms	Myeloproliferative disease	1.26	3.91E-06	1006 / 67218	EA
18	650764	*TYMS*	rs150119891	C	T	-98.77	270.2	endocrine/metabolic	Disorders of amino-acid metabolism	48.43	7.90E-06	20 / 14081	AA
20	63695521	*RTEL1*	rs181080831	G	A	180.51	735.22	musculoskeletal	Claw toe (acquired)	25.58	5.75E-07	28 / 61376	EA
							840.2	injuries & poisonings	Rotator cuff (capsule) sprain	74.46	1.26E-06	68 / 13031	AA

Table S7, UKBB PheWAS results: Results of the PheWAS conducted using SAIGE, a method employing generalized linear mixed models and allowing for imbalance between case and control counts, adjusting for genetic relatedness, sex, birth year and the first 4 principal components of ancestry, on approximately 400,000 UK Biobank (UKBB) participants. Results were downloaded from files provided by the University of Michigan PheWeb server (http://pheweb.sph.umich.edu/SAIGE-UKB/about) on 47 available sentinel variants. Results were evaluated at a Bonferroni threshold corrected for the number of phecodes available for each variant (N=1,403). Related to ***STAR Methods*** .

Chr	Pos	Locus	rsNum	Ref	Alt	Trans-population TL Effect size in (bp)	PheWAS Results					
							Phecode	Category	Phenotype	OR	P-Value	Number of Samples
3	169487437	*TERC*	rs12637184	G	A	-59.79	216	neoplasms	Benign neoplasm of skin	0.89	6.9E-10	7722 / 400618
							218.1	neoplasms	Uterine leiomyoma	0.91	7.9E-08	10344 / 204369
							218	neoplasms	Benign neoplasm of uterus	0.92	7.6E-07	10609 / 204369
							208	neoplasms	Benign neoplasm of colon	0.94	2.7E-06	20204 / 386011
							622	genitourinary	Polyp of female genital organs	0.93	9.5E-06	10881 / 208551
							214.1	neoplasms	Lipoma of skin and subcutaneous tissue	0.90	1.1E-05	4611 / 401613
							214	neoplasms	Lipoma	0.91	1.8E-05	6271 / 401613
4	9930219	*SLC2A2*	rs4235345	G	A	16.78	274.1	endocrine/metabolic	Gout	0.67	3.0E-37	3195 / 405198
							274	endocrine/metabolic	Gout and other crystal arthropathies	0.71	4.4E-33	3763 / 405198
5	1285974	*TERT*	rs7705526	C	A	30.04	200	neoplasms	Myeloproliferative disease	1.43	2.6E-13	995 / 404466
							218.1	neoplasms	Uterine leiomyoma	1.10	2.7E-09	10344 / 204369
							218	neoplasms	Benign neoplasm of uterus	1.10	3.2E-09	10609 / 204369
							191	neoplasms	Manlignant and unknown neoplasms of brain and nervous system	1.41	1.3E-08	655 / 407239
							172.2	neoplasms	Other non-epithelial cancer of skin	1.09	2.4E-08	11149 / 395071
							191.1	neoplasms	Cancer of brain and nervous system	1.45	2.4E-08	531 / 407239
							172	neoplasms	Skin cancer	1.08	3.2E-08	13752 / 395071
							191.11	neoplasms	Cancer of brain	1.47	3.6E-08	486 / 407239
							220	neoplasms	Benign neoplasm of ovary	1.24	5.3E-08	1481 / 192900
							702.2	dermatologic	Seborrheic keratosis	0.86	1.4E-07	3092 / 403439
							200.1	neoplasms	Polycythemia vera	1.41	1.2E-05	390 / 401145
5	1287194	*TERT*	rs2853677	G	A	-23.89	172.2	neoplasms	Other non-epithelial cancer of skin	0.90	5.4E-15	11149 / 395071
							172	neoplasms	Skin cancer	0.92	1.9E-11	13752 / 395071
							218	neoplasms	Benign neoplasm of uterus	0.92	2.5E-08	10609 / 204369
							702.2	dermatologic	Seborrheic keratosis	1.15	2.9E-08	3092 / 403439
							218.1	neoplasms	Uterine leiomyoma	0.92	4.7E-08	10344 / 204369
							200	neoplasms	Myeloproliferative disease	0.79	1.9E-07	995 / 404466
							189.2	neoplasms	Cancer of bladder	0.87	3.4E-06	2427 / 404796
							189.21	neoplasms	Malignant neoplasm of bladder	0.87	7.9E-06	2146 / 404796
							702	dermatologic	Degenerative skin conditions and other dermatoses	1.09	1.8E-05	5522 / 398746
5	1292958	*TERT*	rs114616103	C	T	-57.21	208	neoplasms	Benign neoplasm of colon	0.88	2.1E-05	20204 / 386011
6	31783208	*HSPA1A*	rs1008438	A	C	-20.33	557.1	digestive	Celiac disease	2.80	7.1E-178	1855 / 334783
							557	digestive	Intestinal malabsorption (non-celiac)	2.54	1.7E-167	2103 / 334783
							250.1	endocrine/metabolic	Type 1 diabetes	1.37	2.6E-27	2660 / 388756
							242	endocrine/metabolic	Thyrotoxicosis with or without goiter	1.25	4.6E-11	1860 / 391429
							695.7	dermatologic	Prurigo and Lichen	1.35	1.2E-08	779 / 402672
							695	dermatologic	Erythematous conditions	1.18	1.3E-08	2420 / 402672
							244	endocrine/metabolic	Hypothyroidism	1.07	3.5E-08	14871 / 391429
							454	circulatory system	Varicose veins	0.93	6.7E-08	12172 / 369592
							242.1	endocrine/metabolic	Graves' disease	1.44	9.0E-08	457 / 391429
							454.1	circulatory system	Varicose veins of lower extremity	0.93	1.1E-07	11697 / 369592
							580.12	genitourinary	Non-proliferative glomerulonephritis	1.93	2.2E-07	136 / 397602
							250.11	endocrine/metabolic	Type 1 diabetes with ketoacidosis	1.64	2.3E-07	237 / 388756
							244.4	endocrine/metabolic	Hypothyroidism NOS	1.07	3.2E-07	14171 / 391429
							250	endocrine/metabolic	Diabetes mellitus	1.06	4.4E-07	20203 / 388756
							250.7	endocrine/metabolic	Diabetic retinopathy	1.22	4.6E-07	1339 / 396859
							366	sense organs	Cataract	1.06	7.6E-07	20352 / 388609
							250.23	endocrine/metabolic	Type 2 diabetes with ophthalmic manifestations	1.21	1.9E-06	1298 / 388756
							709.2	dermatologic	Sicca syndrome	1.35	4.3E-06	513 / 399404
							250.12	endocrine/metabolic	Type 1 diabetes with renal manifestations	2.16	6.7E-06	76 / 388756
							251	endocrine/metabolic	Other disorders of pancreatic internal secretion	1.24	7.3E-06	943 / 405386
							251.1	endocrine/metabolic	Hypoglycemia	1.24	7.4E-06	939 / 386319
							285	hematopoietic	Other anemias	1.06	8.9E-06	12256 / 390026
							709	dermatologic	Diffuse diseases of connective tissue	1.11	1.4E-05	3463 / 399404
							580.2	genitourinary	Nephrotic syndrome without mention of glomerulonephritis	1.34	1.6E-05	469 / 397602
							371.3	sense organs	Inflammation of eyelids	0.88	3.3E-05	2396 / 399306
10	101274033	*NKX2-3*	rs10883359	A	G	-16.52	555.2	digestive	Ulcerative colitis	0.89	1.4E-05	3195 / 334783
							555	digestive	Inflammatory bowel disease and other gastroenteritis and colitis	0.90	2.3E-05	4528 / 334783
10	105676465	*OBFC1*	rs9420907	C	A	-49.22	427.2	circulatory system	Atrial fibrillation and flutter	0.89	1.1E-09	14820 / 380919
							218	neoplasms	Benign neoplasm of uterus	0.89	3.7E-08	10609 / 204369
							172.11	neoplasms	Melanomas of skin	0.80	4.5E-08	2691 / 395071
							172.1	neoplasms	Melanomas of skin, dx or hx	0.80	4.5E-08	2691 / 395071
							214.1	neoplasms	Lipoma of skin and subcutaneous tissue	0.84	5.2E-08	4611 / 401613
							218.1	neoplasms	Uterine leiomyoma	0.89	6.9E-08	10344 / 204369
							214	neoplasms	Lipoma	0.87	1.2E-07	6271 / 401613
							427	circulatory system	Cardiac dysrhythmias	0.93	6.1E-07	24681 / 380919
							216	neoplasms	Benign neoplasm of skin	0.89	4.5E-06	7722 / 400618
							704	dermatologic	Diseases of hair and hair follicles	0.88	2.2E-05	5344 / 402357
							706	dermatologic	Diseases of sebaceous glands	0.91	2.5E-05	8948 / 399255
							172	neoplasms	Skin cancer	0.92	2.6E-05	13752 / 395071
							706.2	dermatologic	Sebaceous cyst	0.91	3.6E-05	8876 / 399255
14	73426290	*DCAF4*	rs2572	C	T	27.97	401	circulatory system	Hypertension	1.05	2.1E-05	77977 / 330366
							401.1	circulatory system	Essential hypertension	1.05	0.0000238	77723 / 330366
20	62309554	*RTEL1*	rs41309367	C	T	-34.10	555	digestive	Inflammatory bowel disease and other gastroenteritis and colitis	1.11	0.00000878	4528 / 334783
20	62326874	*RTEL1*	rs181080831	G	A	180.51	165.1	neoplasms	Cancer of bronchus; lung	6.75	0.00000512	2101 / 406226
							165	neoplasms	Cancer within the respiratory system	4.85	0.0000119	2700 / 406226
							214	neoplasms	Lipoma	2.50	0.0000243	6271 / 401613

Table S8, dbGaP Phs IDs by Study: Project identifiers for dbGaP data accession by study. Related to ***STAR Methods*** **.**

Study	Phs ID
AFLMU	phs001543
Amish	phs000956
ARIC	phs001211
BioMe	phs001644
CAMP	phs001726
CARDIA	phs001612
CARE_BADGER	phs001728
CARE_CLIC	phs001729
CARE_PACT	phs001730
CARE_TREXA	phs001732
CFS	phs000954
ChildrensHS_GAP	phs001602
ChildrensHS_IGERA	phs001603
ChildrensHS_MetaAir	phs001604
CHIRAH	phs001605
CHS	phs001368
COPDGene	phs000951
CRA	phs000988
DHS	phs001412
ECLIPSE	phs001472
EOCOPD	phs000946
FHS	phs000974
GALAI	phs001542
GALAII	phs000920
GeneSTAR	phs001218
GENOA	phs001345
GenSalt	phs001217
GOLDN	phs001359
HCHS_SOL	phs001395
HVH	phs000993
HyperGEN	phs001293
IPF	phs001607
JHS	phs000964
LTRC	phs001662
Mayo_VTE	phs001402
MESA	phs001416
MLOF	phs001515
OMG_SCD	phs001608
PCGC_CHD	phs001735
PharmHU	phs001466
PIMA	phs001727
PUSH_SCD	phs001682
REDS-III_Brazil	phs001468
SAFS	phs001215
SAGE	phs000921
SAPPHIRE_asthma	phs001467
SARP	phs001446
Samoan	phs000972
THRV	phs001387
VAFAR	phs000997
VU_AF	phs001032
walk_PHaSST	phs001514
WGHS	phs001040
WHI	phs001237

Supplemental Note 1

There are limitations to the interpretation of effect sizes from genetic studies of TL that are related to the heterogeneity and lack of standardization of various TL assays themselves, therefore hindering comparability of results (e.g., genetic effect sizes) between studies (reviewed in Aubert & Lansdorp, 2008[S1]; Nussey et al., 2014[S2]). This is an area receiving considerable attention with extensive work being done within the recently funded Telomere Research Network (https://trn.tulane.edu/). Most prior, and all the large-scale (N>5000) GWAS rely on "relative TL" measured using qPCR. While cost-effective and high throughput, this measurement technology has its own technical limitations[S3] and does not provide an estimate of TL in base pairs (bp). In fact, most prior GWAS leave effect sizes on the T/R ratio scale output by qPCR; else a post-hoc transformation is necessary to translate T/R ratio into bp as done by Codd et al[S4] using age effects to rescale. In contrast, both TelSeq and Southern blot report TL in terms of bp, but also have some important features to note: The mean terminal restriction fragments (mTRF) measured by Southern blot include both the canonical telomeric TTAGGG repeats and non-canonical sub-telomeric regions of the total cell population[S5]. Southern blot is also labor intensive and requires high amounts of starting DNA generally making it difficult for large GWAS-style studies. TelSeq[S6] estimates the length of the TTAGGG repeat region, by counting sequencing reads containing a certain number of copies of this hexamer. Therefore TelSeq estimates can depend on the value of k (the number of hexamer repeats) used in the estimation, which in turns depends on sequencing read length. However TelSeq has the advantage of leveraging pre-existing sequence data as illustrated in our TOPMed work, i.e., it does not require further wet-lab work to generate TL estimates. Finally,

we note that Southern blot, as well as other techniques that can determine TL of single cell populations or individual telomere lengths[S7], are labor-intensive and not yet amenable to high-throughput, large-scale implementation to be used in GWAS.

REFERENCES

S1. Aubert G, Lansdorp PM. Telomeres and aging. Physiol Rev. 2008;88(2):557-79.
S2. Nussey DH, Baird D, Barrett E, Boner W, Fairlie J, Gemmell N, Hartmann N, Horn T, Haussmann M, Olsson M, et al. Measuring telomere length and telomere dynamics in evolutionary biology and ecology. Methods Ecol Evol. 2014;5(4):299-310.
S3. Nettle D, Gadalla SM, Lai TP, Susser E, Bateson M, Aviv A. Telomere length measurement for longitudinal analysis: implications of assay precision. Am J Epidemiol. 2021.
S4. Codd V, Nelson CP, Albrecht E, Mangino M, Deelen J, Buxton JL, Hottenga JJ, Fischer K, Esko T, Surakka I, et al. Identification of seven loci affecting mean telomere length and their association with disease. Nat Genet. 2013;45(4):422-7, 7e1-2.
S5. Kimura M, Aviv A. Measurement of telomere DNA content by dot blot analysis. Nucleic Acids Res. 2011;39(12):e84.
S6. Ding Z, Mangino M, Aviv A, Spector T, Durbin R, Consortium UK. Estimating telomere length from whole genome sequence data. Nucleic Acids Res. 2014;42(9):e75.
S7. Uppuluri L, Varapula D, Young E, Riethman H, Xiao M. Single-molecule telomere length characterization by optical mapping in nano-channel array: Perspective and review on telomere length measurement. Environ Toxicol Pharmacol. 2021;82:103562.

Study Acknowledgements

Generation of TOPMed whole genome sequencing data by study:
Whole genome sequencing (WGS) for the Trans-Omics in Precision Medicine (TOPMed) program was supported by the National Heart, Lung and Blood Institute (NHLBI). WGS for NHLBI TOPMed: AFLMU (phs001543) was performed at Broad Genomics (3UM1HG008895-01S2; HHSN268201500014C); WGS for NHLBI TOPMed: Amish (phs000956) was performed at Broad Genomics (3R01HL121007-01S1); WGS for NHLBI TOPMed: ARIC (phs001211) was performed at Baylor (3U54HG003273-12S2 / HHSN268201500015C,3R01HL092577-06S1), Broad Genomics (3U54HG003273-12S2 / HHSN268201500015C,3R01HL092577-06S1); WGS for NHLBI TOPMed: BioMe (phs001644) was performed at MGI (HHSN268201600037I,HHSN268201600033I,3UM1HG008853-01S2), Baylor (HHSN268201600037I,HHSN268201600033I,3UM1HG008853-01S2); WGS for NHLBI TOPMed: CAMP (phs001726) was performed at NWGC (HHSN268201600032I); WGS for NHLBI TOPMed: CARDIA (phs001612) was performed at Baylor (HHSN268201600033I); WGS for NHLBI TOPMed: CARE_BADGER (phs001728) was performed at NWGC (HHSN268201600032I); WGS for NHLBI TOPMed: CARE_CLIC (phs001729) was performed at NWGC (HHSN268201600032I); WGS for NHLBI TOPMed: CARE_PACT (phs001730) was performed at NWGC (HHSN268201600032I); WGS for NHLBI TOPMed: CARE_TREXA (phs001732) was performed at NWGC (HHSN268201600032I); WGS for NHLBI TOPMed: CFS (phs000954) was performed at NWGC (HHSN268201600032I,3R01HL098433-05S1); WGS for NHLBI TOPMed: ChildrensHS_GAP (phs001602) was performed at NWGC (HHSN268201600032I); WGS for NHLBI TOPMed: ChildrensHS_IGERA (phs001603) was performed at NWGC (HHSN268201600032I); WGS for NHLBI TOPMed: ChildrensHS_MetaAir (phs001604) was performed at NWGC (HHSN268201600032I); WGS for NHLBI TOPMed: CHIRAH (phs001605) was performed at NWGC (HHSN268201600032I); WGS for NHLBI TOPMed: CHS (phs001368) was performed at Baylor (HHSN268201600033I,3U54HG003273-12S2 / HHSN268201500015C); WGS for NHLBI TOPMed: COPDGene (phs000951) was performed at NWGC (3R01HL089856-08S1,HHSN268201500014C), Broad Genomics (3R01HL089856-08S1,HHSN268201500014C); WGS for NHLBI TOPMed: CRA (phs000988) was performed at NWGC (3R37HL066289-13S1,HHSN268201600032I); WGS for NHLBI TOPMed: DHS (phs001412) was performed at Broad Genomics (HHSN268201500014C); WGS for NHLBI TOPMed: ECLIPSE (phs001472) was performed at MGI (HHSN268201600037I); WGS for NHLBI TOPMed: EOCOPD (phs000946) was performed at NWGC (3R01HL089856-08S1); WGS for NHLBI TOPMed: FHS (phs000974) was performed at Broad Genomics (3U54HG003067-12S2,3R01HL092577-06S1); WGS for NHLBI TOPMed: GALAI (phs001542) was performed at NWGC (HHSN268201600032I); WGS for NHLBI TOPMed: GALAII (phs000920) was performed at NYGC (3R01HL117004-02S3,HHSN268201600032I), NWGC (3R01HL117004-02S3,HHSN268201600032I), NYGC (UM1 HG008901); WGS for NHLBI TOPMed: GeneSTAR (phs001218) was performed at Psomagen (3R01HL112064-04S1,R01HL112064,HHSN268201500014C), Illumina (3R01HL112064-04S1,R01HL112064,HHSN268201500014C), Broad Genomics (3R01HL112064-04S1,R01HL112064,HHSN268201500014C); WGS for NHLBI TOPMed: GENOA (phs001345) was performed at NWGC (3R01HL055673-18S1,HHSN268201500014C), Broad Genomics (3R01HL055673-18S1,HHSN268201500014C); WGS for NHLBI TOPMed: GenSalt (phs001217) was performed at Baylor (HHSN268201500015C); WGS for NHLBI TOPMed: GOLDN (phs001359) was performed at NWGC (3R01HL104135-04S1); WGS for NHLBI TOPMed: HCHS_SOL (phs001395) was performed at Baylor (HHSN268201600033I); WGS for NHLBI TOPMed: HVH (phs000993) was performed at Broad Genomics (3R01HL092577-06S1,3U54HG003273-12S2 / HHSN268201500015C), Baylor (3R01HL092577-06S1,3U54HG003273-12S2 / HHSN268201500015C); WGS for NHLBI TOPMed: HyperGEN (phs001293) was performed at NWGC (3R01HL055673-18S1); WGS for NHLBI TOPMed: IPF (phs001607) was performed at MGI (HHSN268201600037I); WGS for NHLBI TOPMed: JHS (phs000964) was performed at NWGC (HHSN268201100037C); WGS for NHLBI TOPMed: LTRC

(phs001662) was performed at Broad Genomics (HHSN268201600034I); WGS for NHLBI TOPMed: Mayo_VTE (phs001402) was performed at Baylor (3U54HG003273-12S2 / HHSN268201500015C); WGS for NHLBI TOPMed: MESA (phs001416) was performed at Broad Genomics (3U54HG003067-13S1,HHSN268201500014C); WGS for NHLBI TOPMed: MLOF (phs001515) was performed at Baylor (HHSN268201600033I,HHSN268201500016C), NYGC (HHSN268201600033I,HHSN268201500016C); WGS for NHLBI TOPMed: OMG_SCD (phs001608) was performed at Baylor (HHSN268201500015C); WGS for NHLBI TOPMed: PCGC_CHD (phs001735) was performed at Broad Genomics (HHSN268201600034I); WGS for NHLBI TOPMed: PharmHU (phs001466) was performed at Baylor (HHSN268201500015C); WGS for NHLBI TOPMed: PIMA (phs001727) was performed at NWGC (HHSN268201600032I); WGS for NHLBI TOPMed: PUSH_SCD (phs001682) was performed at Baylor (HHSN268201500015C); WGS for NHLBI TOPMed: REDS-III_Brazil (phs001468) was performed at Baylor (HHSN268201500015C); WGS for NHLBI TOPMed: SAFS (phs001215) was performed at Illumina (R01HL113322,3R01HL113323-03S1); WGS for NHLBI TOPMed: SAGE (phs000921) was performed at NYGC (3R01HL117004-02S3,HHSN268201600032I), NWGC (3R01HL117004-02S3,HHSN268201600032I); WGS for NHLBI TOPMed: SAPPHIRE_asthma (phs001467) was performed at NWGC (HHSN268201600032I); WGS for NHLBI TOPMed: SARP (phs001446) was performed at NYGC (HHSN268201500016C); WGS for NHLBI TOPMed: SAS (phs000972) was performed at NWGC (HHSN268201100037C,HHSN268201500016C), NYGC (HHSN268201100037C,HHSN268201500016C); WGS for NHLBI TOPMed: THRV (phs001387) was performed at Baylor (3R01HL111249-04S1 / HHSN26820150015C); WGS for NHLBI TOPMed: VAFAR (phs000997) was performed at Broad Genomics (3U54HG003067-12S2 / 3U54HG003067-13S1; 3UM1HG008895-01S2; 3UM1HG008895-01S2,3R01HL092577-06S1); WGS for NHLBI TOPMed: VU_AF (phs001032) was performed at Broad Genomics (3R01HL092577-06S1); WGS for NHLBI TOPMed: walk_PHaSST (phs001514) was performed at Baylor (HHSN268201500015C); WGS for NHLBI TOPMed: WGHS (phs001040) was performed at Broad Genomics (3R01HL092577-06S1); WGS for NHLBI TOPMed: WHI (phs001237) was performed at Broad Genomics (HHSN268201500014C). Core support including centralized genomic read mapping and genotype calling, along with variant quality metrics and filtering were provided by the TOPMed Informatics Research Center (3R01HL-117626-02S1; contract HHSN268201800002I). Core support including phenotype harmonization, data management, sample-identity QC, and general program coordination were provided by the TOPMed Data Coordinating Center (R01HL-120393; U01HL-120393; contract HHSN268201800001I). We gratefully acknowledge the studies and participants who provided biological samples and data for TOPMed. NYGC = New York Genome Center; Broad Genomics = Broad Institute Genomics Platform; NWGC = University of Washington Northwest Genomics Center; Illumina = Illumina Genomic Services; Psomagen = Psomagen Corp.; Baylor = Baylor Human Genome Sequencing Center; MGI = McDonnell Genome Institute

NHLBI TOPMed: Atrial Fibrillation Biobank LMU (AFLMU) in the context of the Arrhythmia-Biobank-LMU

AFLMU is a repository of AF patients recruited in the context of the German Competence Network for Atrial Fibrillation (AFNET) and at the Department of Medicine I of the University Hospital Munich. In this context, DNA samples were preferentially sampled if the patient developed AF before the age of 60 years. Cases were selected if the diagnosis of atrial fibrillation was made on an electrocardiogram analyzed by a trained physician. Patients with signs of moderate to severe heart failure, moderate to severe valve disease or with hyperthyroidism were excluded from the study. All participants provided written informed consent. AFLMU was approved by the Ethics Committee at the Ludwig-Maximilian's University.

NHLBI TOPMed: Genetics of Cardiometabolic Health in the Amish (Amish)

The Amish studies upon which these data are based were supported by NIH grants R01 AG18728, U01 HL072515, R01 HL088119, R01 HL121007, and P30 DK072488. See publication: PMID: 18440328

NHLBI TOPMed: Atherosclerosis Risk in Communities (ARIC)
The Atherosclerosis Risk in Communities study has been funded in whole or in part with Federal funds from the National Heart, Lung, and Blood Institute, National Institutes of Health, Department of Health and Human Services (contract numbers HHSN268201700001I, HHSN268201700002I, HHSN268201700003I, HHSN268201700004I and HHSN268201700005I). The authors thank the staff and participants of the ARIC study for their important contributions.

NHLBI TOPMed: The Genetics and Epidemiology of Asthma in Barbados (BAGS)
We gratefully acknowledge the contributions of Pissamai and Trevor Maul, Paul Levett, Anselm Hennis, P. Michele Lashley, Raana Naidu, Malcolm Howitt and Timothy Roach, and the numerous health care providers, and community clinics and co-investigators who assisted in the phenotyping and collection of DNA samples, and the families and patients for generously donating DNA samples to the Barbados Asthma Genetics Study (BAGS). The Genetics and Epidemiology of Asthma in Barbados is supported by National Institutes of Health (NIH) National Heart, Lung, Blood Institute TOPMed (R01 HL104608-S1) and: R01 AI20059, K23 HL076322, R01HL087699, and RC2 HL101651. For the specific cohort descriptions and descriptions regarding the collection of phenotype data can be found at: https://www.nhlbiwgs.org/group/bags-asthma. The authors wish to give special recognition to the individual study participants who provided biological samples and or data, without their support in research none of this would be possible.

NHLBI TOPMed: BioMe Biobank at Mount Sinai (BioMe)
The Mount Sinai BioMe Biobank has been supported by The Andrea and Charles Bronfman Philanthropies and in part by Federal funds from the NHLBI and NHGRI (U01HG00638001; U01HG007417; X01HL134588). We thank all participants in the Mount Sinai Biobank. We also thank all our recruiters who have assisted and continue to assist in data collection and management and are grateful for the computational resources and staff expertise provided by Scientific Computing at the Icahn School of Medicine at Mount Sinai.

NHLBI TOPMed: CAMP
We thank the clinical centers and the Data Coordinating Center of the Childhood Asthma Management Program (CAMP) as well as all of the study participants at the 8 clinical sites. The CAMP study was supported by NHLBI P01 HL132825.

NHLBI TOPMed: Coronary Artery Risk Development in Young Adults Study (CARDIA)
The Coronary Artery Risk Development in Young Adults Study (CARDIA) is conducted and supported by the National Heart, Lung, and Blood Institute (NHLBI) in collaboration with the University of Alabama at Birmingham (HHSN268201800005I & HHSN268201800007I), Northwestern University (HHSN268201800003I), University of Minnesota (HHSN268201800006I), and Kaiser Foundation Research Institute (HHSN268201800004I). CARDIA was also partially supported by the Intramural Research Program of the National Institute on Aging (NIA) and an intra-agency agreement between NIA and NHLBI (AG0005).

NHLBI TOPMed: CARE_BADGER
This research was supported by grants from the National Heart, Lung, and Blood Institute (NHLBI), ((5U10HL064287, 5U10HL064288, 5U10HL064295, 5U10HL064307, 5U10HL064305, 5U10HL064313, and HL080083)

NHLBI TOPMed: CARE_CLIC

This research was supported by grants from the National Heart, Lung, and Blood Institute (NHLBI), ((5U10HL064287, 5U10HL064288, 5U10HL064295, 5U10HL064307, 5U10HL064305, 5U10HL064313, and HL080083)

NHLBI TOPMed: CARE_PACT
This research was supported by grants from the National Heart, Lung, and Blood Institute (NHLBI), ((5U10HL064287, 5U10HL064288, 5U10HL064295, 5U10HL064307, 5U10HL064305, 5U10HL064313, and HL080083)

NHLBI TOPMed: CARE_TREXA
This research was supported by grants from the National Heart, Lung, and Blood Institute (NHLBI), ((5U10HL064287, 5U10HL064288, 5U10HL064295, 5U10HL064307, 5U10HL064305, 5U10HL064313, and HL080083)

NHLBI TOPMed: The Cleveland Family Study (CFS)
The Cleveland Family Study has been supported in part by National Institutes of Health grants [R01-HL046380, KL2-RR024990, R35-HL135818, and R01-HL113338].

NHLBI TOPMed: Children's Health Study: Integrative Genetic Approaches to Gene-Air Pollution Interactions in Asthma (ChildrensHS_GAP)
The Integrative Genetic Approaches to Gene-Air Pollution Interactions in Asthma (GAP) study was supported by the National Institute of Environmental Health Sciences (NIEHS) grant # R01ES021801. The Children's Health Study (CHS) was supported by the Southern California Environmental Health Sciences Center (grant P30ES007048); National Institute of Environmental Health Sciences (grants 5P01ES011627, ES021801, ES023262, P01ES009581, P01ES011627, P01ES022845, R01 ES016535, R03ES014046, P50 CA180905, R01HL061768, R01HL076647, R01HL087680 and RC2HL101651), the Environmental Protection Agency (grants RD83544101, R826708, RD831861, and R831845), and the Hastings Foundation.

NHLBI TOPMed: Children's Health Study: Integrative Genomics and Environmental Research of Asthma (ChildrensHS_IGERA)
The Integrative Genomics and Environmental Research of Asthma (IGERA) Study was supported by the National Heart, Lung and Blood Institute (grant # RC2HL101543 -The Asthma BioRepository for Integrative Genomics Research, PI Gilliland/Raby). The Children's Health Study (CHS) was supported by the Southern California Environmental Health Sciences Center (grant P30ES007048); National Institute of Environmental Health Sciences (grants 5P01ES011627, ES021801, ES023262, P01ES009581, P01ES011627, P01ES022845, R01 ES016535, R03ES014046, P50 CA180905, R01HL061768, R01HL076647, R01HL087680 and RC2HL101651), the Environmental Protection Agency (grants RD83544101, R826708, RD831861, and R831845), and the Hastings Foundation.

NHLBI TOPMed: Children's Health Study: Effects of Air Pollution on the Development of Obesity in Children (ChildrensHS_MetaAir)
The Effects of Air Pollution on the Development of Obesity in Children (Meta-AIR) study was supported by the Southern California Children’s Environmental Health Center funded by the National Institute of Environmental Health Sciences (NIEHS) (P01ES022845) and the Environmental Protection Agency (EPA) (RD-83544101–0). The Children's Health Study (CHS) was supported by the Southern California Environmental Health Sciences Center (grant P30ES007048); National Institute of Environmental Health Sciences (grants 5P01ES011627, ES021801, ES023262, P01ES009581, P01ES011627, P01ES022845, R01 ES016535, R03ES014046, P50 CA180905, R01HL061768, R01HL076647, R01HL087680 and

RC2HL101651), the Environmental Protection Agency (grants RD83544101, R826708, RD831861, and R831845), and the Hastings Foundation.

NHLBI TOPMed: Genetics Sub-Study of Chicago Initiative to Raise Asthma Health Equity (CHIRAH)
Support for the Genetics Sub-Study of Chicago Initiative to Raise Asthma Health Equity was provided by NHLBI grant number UO1 HL072496.

NHLBI TOPMed: Cardiovascular Health Study (CHS)
This research was supported by contracts HHSN268201200036C, HHSN268200800007C, HHSN268201800001C, N01HC55222, N01HC85079, N01HC85080, N01HC85081, N01HC85082, N01HC85083, N01HC85086, and 75N92021D00006, and grants U01HL080295 and U01HL130114 from the National Heart, Lung, and Blood Institute (NHLBI), with additional contribution from the National Institute of Neurological Disorders and Stroke (NINDS). Additional support was provided by R01AG023629 from the National Institute on Aging (NIA). A full list of principal CHS investigators and institutions can be found at CHS-NHLBI.org. The content is solely the responsibility of the authors and does not necessarily represent the official views of the National Institutes of Health.

NHLBI TOPMed: Genetic Epidemiology of COPD (COPDGene) in the TOPMed Program
The COPDGene project described was supported by Award Number U01 HL089897 and Award Number U01 HL089856 from the National Heart, Lung, and Blood Institute. The content is solely the responsibility of the authors and does not necessarily represent the official views of the National Heart, Lung, and Blood Institute or the National Institutes of Health. The COPDGene project is also supported by the COPD Foundation through contributions made to an Industry Advisory Board comprised of AstraZeneca, Boehringer Ingelheim, GlaxoSmithKline, Novartis, Pfizer, Siemens and Sunovion. A full listing of COPDGene investigators can be found at: http://www.copdgene.org/directory

NHLBI TOPMed: The Genetic Epidemiology of Asthma in Costa Rica (CRA)
This study was supported by NHLBI grants R37 HL066289 and P01 HL132825. We wish to acknowledge the investigators at the Channing Division of Network Medicine at Brigham and Women's Hospital, the investigators at the Hospital Nacional de Niños in San José, Costa Rica and the study subjects and their extended family members who contributed samples and genotypes to the study, and the NIH/NHLBI for its support in making this project possible.

NHLBI TOPMed: Diabetes Heart Study (DHS)
This work was supported by R01 HL92301, R01 HL67348, R01 NS058700, R01 AR48797, R01 DK071891, R01 AG058921, the General Clinical Research Center of the Wake Forest University School of Medicine (M01 RR07122, F32 HL085989), the American Diabetes Association, and a pilot grant from the Claude Pepper Older Americans Independence Center of Wake Forest University Health Sciences (P60 AG10484).

NHLBI TOPMed: ECLIPSE
The ECLIPSE study (NCT00292552) was sponsored by GlaxoSmithKline. The ECLIPSE investigators included: ECLIPSE Investigators — Bulgaria: Y. Ivanov, Pleven; K. Kostov, Sofia. Canada: J. Bourbeau, Montreal; M. Fitzgerald, Vancouver, BC; P. Hernandez, Halifax, NS; K. Killian, Hamilton, ON; R. Levy, Vancouver, BC; F. Maltais, Montreal; D. O'Donnell, Kingston, ON. Czech Republic: J. Krepelka, Prague. Denmark: J. Vestbo, Hvidovre. The Netherlands: E. Wouters, Horn-Maastricht. New Zealand: D. Quinn, Wellington. Norway: P. Bakke, Bergen. Slovenia: M. Kosnik, Golnik. Spain: A. Agusti, J. Sauleda, P. de Mallorca. Ukraine: Y. Feschenko, V. Gavrisyuk, L. Yashina, Kiev; N. Monogarova, Donetsk. United Kingdom: P. Calverley, Liverpool; D. Lomas, Cambridge; W. MacNee, Edinburgh; D. Singh, Manchester; J. Wedzicha, London. United States: A. Anzueto, San Antonio, TX; S. Braman, Providence,

RI; R. Casaburi, Torrance CA; B. Celli, Boston; G. Giessel, Richmond, VA; M. Gotfried, Phoenix, AZ; G. Greenwald, Rancho Mirage, CA; N. Hanania, Houston; D. Mahler, Lebanon, NH; B. Make, Denver; S. Rennard, Omaha, NE; C. Rochester, New Haven, CT; P. Scanlon, Rochester, MN; D. Schuller, Omaha, NE; F. Sciurba, Pittsburgh; A. Sharafkhaneh, Houston; T. Siler, St. Charles, MO; E. Silverman, Boston; A. Wanner, Miami; R. Wise, Baltimore; R. ZuWallack, Hartford, CT. ECLIPSE Steering Committee: H. Coxson (Canada), C. Crim (GlaxoSmithKline, USA), L. Edwards (GlaxoSmithKline, USA), D. Lomas (UK), W. MacNee (UK), E. Silverman (USA), R. Tal-Singer (Co-chair, GlaxoSmithKline, USA), J. Vestbo (Co-chair, Denmark), J. Yates (GlaxoSmithKline, USA). ECLIPSE Scientific Committee: A. Agusti (Spain), P. Calverley (UK), B. Celli (USA), C. Crim (GlaxoSmithKline, USA), B. Miller (GlaxoSmithKline, USA), W. MacNee (Chair, UK), S. Rennard (USA), R. Tal-Singer (GlaxoSmithKline, USA), E. Wouters (The Netherlands), J. Yates (GlaxoSmithKline, USA).

NHLBI TOPMed: Boston Early-Onset COPD Study in the TOPMed Program (EOCOPD)
The Boston Early-Onset COPD Study was supported by R01 HL113264 and U01 HL089856 from the National Heart, Lung, and Blood Institute.

NHLBI TOPMed: Whole Genome Sequencing and Related Phenotypes in the Framingham Heart Study (FHS)
The Framingham Heart Study (FHS) acknowledges the support of contracts NO1-HC-25195, HHSN268201500001I, and 75N92019D00031 from the National Heart, Lung and Blood Institute and grant supplement R01 HL092577-06S1 for this research. We also acknowledge the dedication of the FHS study participants without whom this research would not be possible.

NHLBI TOPMed: Genes-environments and Admixture in Latino Asthmatics (GALA I) Study
The Genes-environments and Admixture in Latino Americans (GALA I) Study was supported by the National Heart, Lung, and Blood Institute of the National Institute of Health (NIH) grants R01HL117004 and X01HL134589; study enrollment supported by Sandler Center for Basic Research in Asthma and the Sandler Family Foundation, the American Asthma Foundation, the American Lung Association, the NIH grants K23HL04464 and HL07185, the Resource Centers for Minority Aging Research from the National Institute on Aging, RCMAR P30-AG15272, the National Institute of Nursing Research and the National Center on Minority Health and Health Disparities.

NHLBI TOPMed: Genes-environments and Admixture in Latino Asthmatics (GALA II) Study
The Genes-environments and Admixture in Latino Americans (GALA II) Study was supported by the National Heart, Lung, and Blood Institute of the National Institute of Health (NIH) grants R01HL117004 and X01HL134589; study enrollment supported by the Sandler Family Foundation, the American Asthma Foundation, the RWJF Amos Medical Faculty Development Program, Harry Wm. and Diana V. Hind Distinguished Professor in Pharmaceutical Sciences II and the National Institute of Environmental Health Sciences grant R01ES015794 .
WGS of part of GALA II was performed by New York Genome Center under The Centers for Common Disease Genomics of the Genome Sequencing Program (GSP) Grant (UM1 HG008901). The GSP Coordinating Center (U24 HG008956) contributed to cross-program scientific initiatives and provided logistical and general study coordination. GSP is funded by the National Human Genome Research Institute, the National Heart, Lung, and Blood Institute, and the National Eye Institute.
The GALA II study collaborators include Shannon Thyne, UCSF; Harold J. Farber, Texas Children's Hospital; Denise Serebrisky, Jacobi Medical Center; Rajesh Kumar, Lurie Children's Hospital of Chicago; Emerita Brigino-Buenaventura, Kaiser Permanente; Michael A. LeNoir, Bay Area Pediatrics; Kelley Meade, UCSF Benioff Children’s Hospital, Oakland; William Rodriguez-Cintron, VA Hospital, Puerto Rico; Pedro C. Avila, Northwestern University; Jose R. Rodriguez-Santana, Centro de Neumologia Pediatrica; Luisa N. Borrell, City University of New York; Adam Davis, UCSF Benioff Children's Hospital, Oakland; Saunak Sen, University of Tennessee and Fred Lurmann, Sonoma

Technologies, Inc. The authors acknowledge the families and patients for their participation and thank the numerous health care providers and community clinics for their support and participation in GALA II. In particular, the authors thank study coordinator Sandra Salazar; the recruiters who obtained the data: Duanny Alva, MD, Gaby Ayala-Rodriguez, Lisa Caine, Elizabeth Castellanos, Jaime Colon, Denise DeJesus, Blanca Lopez, Brenda Lopez, MD, Louis Martos, Vivian Medina, Juana Olivo, Mario Peralta, Esther Pomares, MD, Jihan Quraishi, Johanna Rodriguez, Shahdad Saeedi, Dean Soto, Ana Taveras; and the lab researcher Celeste Eng who processed the biospecimens.

NHLBI TOPMed: GeneSTAR (Genetic Study of Atherosclerosis Risk)
The Johns Hopkins Genetic Study of Atherosclerosis Risk (GeneSTAR) was supported by grants from the National Institutes of Health through the National Heart, Lung, and Blood Institute (U01HL72518, HL087698, HL112064) and by a grant from the National Center for Research Resources (M01-RR000052) to the Johns Hopkins General Clinical Research Center. We would like to thank the participants and families of GeneSTAR and our dedicated staff for all their sacrifices.

NHLBI TOPMed: Genetic Epidemiology Network of Arteriopathy (GENOA)
Support for GENOA was provided by the National Heart, Lung and Blood Institute (HL054457, HL054464, HL054481, HL119443, and HL087660) of the National Institutes of Health.

NHLBI TOPMed: Genetic Epidemiology Network of Salt Sensitivity (GenSalt)
The Genetic Epidemiology Network of Salt-Sensitivity (GenSalt) was supported by research grants (U01HL072507, R01HL087263, and R01HL090682) from the National Heart, Lung and Blood Institute, National Institutes of Health, Bethesda, MD.

NHLBI TOPMed: Genetics of Lipid Lowering Drugs and Diet Network (GOLDN)
GOLDN biospecimens, baseline phenotype data, and intervention phenotype data were collected with funding from National Heart, Lung and Blood Institute (NHLBI) grant U01 HL072524. Whole-genome sequencing in GOLDN was funded by NHLBI grant R01 HL104135 and supplement R01 HL104135-04S1.

NHLBI TOPMed: Hispanic Community Health Study/Study of Latinos (HCHS_SOL)
The Hispanic Community Health Study/Study of Latinos is a collaborative study supported by contracts from the National Heart, Lung, and Blood Institute (NHLBI) to the University of North Carolina (HHSN268201300001I / N01-HC-65233), University of Miami (HHSN268201300004I / N01-HC-65234), Albert Einstein College of Medicine (HHSN268201300002I / N01-HC-65235), University of Illinois at Chicago – HHSN268201300003I / N01-HC-65236 Northwestern Univ), and San Diego State University (HHSN268201300005I / N01-HC-65237). The following Institutes/Centers/Offices have contributed to the HCHS/SOL through a transfer of funds to the NHLBI: National Institute on Minority Health and Health Disparities, National Institute on Deafness and Other Communication Disorders, National Institute of Dental and Craniofacial Research, National Institute of Diabetes and Digestive and Kidney Diseases, National Institute of Neurological Disorders and Stroke, NIH Institution-Office of Dietary Supplements.

NHLBI TOPMed: Heart and Vascular Health Study (HVH)
The Heart and Vascular Health Study was supported by grants HL068986, HL085251, HL095080, and HL073410 from the National Heart, Lung, and Blood Institute.

NHLBI TOPMed: Hypertension Genetic Epidemiology Network (HyperGEN)
The HyperGEN Study is part of the National Heart, Lung, and Blood Institute (NHLBI) Family Blood Pressure Program; collection of the data represented here was supported by grants U01 HL054472 (MN Lab), U01 HL054473 (DCC), U01 HL054495 (AL FC), and U01 HL054509 (NC FC). The HyperGEN:

Genetics of Left Ventricular Hypertrophy Study was supported by NHLBI grant R01 HL055673 with whole-genome sequencing made possible by supplement -18S1.

NHLBI TOPMed: IPF
This research was supported by the National Heart, Lung and Blood Institute (R01-HL097163, P01-HL092870, and UH3-HL123442) and the Department of Defense (W81XWH-17-1-0597).

NHLBI TOPMed: The Jackson Heart Study (JHS)
The Jackson Heart Study (JHS) is supported and conducted in collaboration with Jackson State University (HHSN268201800013I), Tougaloo College (HHSN268201800014I), the Mississippi State Department of Health (HHSN268201800015I) and the University of Mississippi Medical Center (HHSN268201800010I, HHSN268201800011I and HHSN268201800012I) contracts from the National Heart, Lung, and Blood Institute (NHLBI) and the National Institute on Minority Health and Health Disparities (NIMHD). The authors also wish to thank the staffs and participants of the JHS.

NHLBI TOPMed: LTRC
This study utilized biological specimens and data provided by the Lung Tissue Research Consortium (LTRC) supported by the National Heart, Lung, and Blood Institute (NHLBI). The LTRC was sponsored by a contract from the NHLBI: HHSN2682016000021

NHLBI TOPMed: Mayo Clinic Venous Thromboembolism Study (Mayo_VTE)
Funded, in part, by grants from the National Institutes of Health, National Heart, Lung and Blood Institute (HL66216 and HL83141). the National Human Genome Research Institute (HG04735, HG06379), and research support provided by Mayo Foundation.

NHLBI TOPMed: Multi-Ethnic Study of Atherosclerosis (MESA)
Support for the Multi-Ethnic Study of Atherosclerosis (MESA) projects are conducted and supported by the National Heart, Lung, and Blood Institute (NHLBI) in collaboration with MESA investigators. Support for MESA is provided by contracts 75N92020D00001, HHSN268201500003I, N01-HC-95159, 75N92020D00005, N01-HC-95160, 75N92020D00002, N01-HC-95161, 75N92020D00003, N01-HC-95162, 75N92020D00006, N01-HC-95163, 75N92020D00004, N01-HC-95164, 75N92020D00007, N01-HC-95165, N01-HC-95166, N01-HC-95167, N01-HC-95168, N01-HC-95169, UL1-TR-000040, UL1-TR-001079, and UL1-TR-001420. MESA Family is conducted and supported by the National Heart, Lung, and Blood Institute (NHLBI) in collaboration with MESA investigators. Support is provided by grants and contracts R01HL071051, R01HL071205, R01HL071250, R01HL071251, R01HL071258, R01HL071259, by the National Center for Research Resources, Grant UL1RR033176. The provision of genotyping data was supported in part by the National Center for Advancing Translational Sciences, CTSI grant UL1TR001881, and the National Institute of Diabetes and Digestive and Kidney Disease Diabetes Research Center (DRC) grant DK063491 to the Southern California Diabetes Endocrinology Research Center.

NHLBI TOPMed: My Life, Our Future (MLOF)
The My Life, Our Future samples and data are made possible through the partnership of Bloodworks Northwest, the American Thrombosis and Hemostasis Network, the National Hemophilia Foundation, and Bioverativ. We gratefully acknowledge the hemophilia treatment centers and their patients who provided biological samples and phenotypic data.

NHLBI TOPMed: Outcome Modifying Genes in Sickle Cell Disease (OMG-SCD)
The OMG-SCD study was administered by Marilyn J. Telen, M.D. and Allison E. Ashley-Koch, Ph.D. from Duke University Medical Center, and collection of the data set was supported by grants HL068959

and HL079915 from the National Heart, Lung, and Blood Institute (NHLBI) of the National Institute of Health (NIH).

NHLBI TOPMed: Pediatric Cardiac Genomics Consortium's Congenital Heart Disease Biobank (PCGC-CHD)

The Pediatric Cardiac Genomics Consortium (PCGC) program is funded by the National Heart, Lung, and Blood Institute, National Institutes of Health, U.S. Department of Health and Human Services through grants UM1HL128711, UM1HL098162, UM1HL098147, UM1HL098123, UM1HL128761, and U01HL131003.

NHLBI TOPMed: The Pharmacogenomics of Hydroxyurea in Sickle Cell Disease (PharmHU)

Collection of the PharmHU samples and data were supported in part by the Department of Pediatrics, Baylor College of Medicine funds, National Institutes of Health (NIH) National Institute of Diabetes and Digestive and Kidney Diseases (NIDDK) grant 1K08 DK110448-01, NIH NHLBI R01 HL069234, and U01-HL117721 funded by NHLBI. We are very grateful to the patients with sickle cell disease for their participation in PharmHU.

NHLBI TOPMed: PIMA

This research was supported by grants from the National Heart, Lung, and Blood Institute (NHLBI), ((5U10HL064287, 5U10HL064288, 5U10HL064295, 5U10HL064307, 5U10HL064305, 5U10HL064313, and HL080083)

NHLBI TOPMed: PUSH_SCD

We thank Dr. Victor R Gordeuk and the investigators of the PUSH study and the patients who participated in the study. We also thank the PUSH clinical site team: Howard University: Victor R Gordeuk, Sergei Nekhai, Oswaldo Castro, Sohail Rana, Mehdi Nouraie, James G Taylor 6th, Children National Medical Center: Caterina Minniti, Deepika Darbari, Lori Lutchman-Jones, Nitti Dham, Craig Sable, NHLBI: Mark Gladwin, Greg Kato. University of Michigan: Andrew Campbell, Gregory Ensing, Manuel Arteta, Special thanks to the volunteers who participated in the PUSH study. This project was funded with federal funds from the NHLBI, NIH. Detail description of the study was published in Haematologica. 2009 Mar;94(3):340-7, MInniti C, et al. “Elevated tricuspid regurgitant jet velocity in children and adolescents with sickle cell disease: association with hemolysis and hemoglobin oxygen desaturation.”

NHLBI TOPMed: Recipient Epidemiology and Donor Evaluation Study-III (REDS-III_Brazil)

The Recipient Epidemiology and Donor Evaluation Study (REDS)-III was funded by NIH NHLBI contract HHSN268201100007I and conducted under the leadership of Simone Glynn (NHLBI), and principle investigators Brian Custer and Ester Sabino. We are grateful to the Brazilian sickle cell disease patients who participated in the REDS-III study and provided blood samples for whole genome sequencing as well as the REDS-III staff: Vitalant Research Institute (Shannon Kelly), University of Sao Paulo (Miriam V Flor Park, Ligia Capuani), Hemominas Belo Horizonte (Anna Barbara Proietti), Hemominas Montes Claros (Rosimere Alfonso), Hemominas Juiz de Fora (Daniela de O. Werneck Rodrigues), Hemope (Paula Loureiro), Hemorio (Claudia Maximo).

NHLBI TOPMed: San Antonio Family Heart Study (SAFS)

Collection of the San Antonio Family Study data was supported in part by National Institutes of Health (NIH) grants P01 HL045522, R01 MH078143, R01 MH078111 and R01 MH083824; and whole genome sequencing of SAFS subjects was supported by U01 DK085524 and R01 HL113323. We are very grateful to the participants of the San Antonio Family Study for their continued involvement in our research programs.

NHLBI TOPMed: Study of African Americans, Asthma, Genes and Environment (SAGE)
The Study of African Americans, Asthma, Genes and Environments (SAGE) was supported by by the National Heart, Lung, and Blood Institute of the National Institute of Health (NIH) grants R01HL117004 and X01HL134589; study enrollment supported by the Sandler Family Foundation, the American Asthma Foundation, the RWJF Amos Medical Faculty Development Program, Harry Wm. and Diana V. Hind Distinguished Professor in Pharmaceutical Sciences II. The SAGE study collaborators include Harold J. Farber, Texas Children's Hospital; Emerita Brigino-Buenaventura, Kaiser Permanente; Michael A. LeNoir, Bay Area Pediatrics; Kelley Meade, UCSF Benioff Children's Hospital, Oakland; Luisa N. Borrell, City University of New York; Adam Davis, UCSF Benioff Children's Hospital, Oakland and Fred Lurmann, Sonoma Technologies, Inc. The authors acknowledge the families and patients for their participation and thank the numerous health care providers and community clinics for their support and participation in SAGE. In particular, the authors thank study coordinator Sandra Salazar; the recruiters who obtained the data: Lisa Caine, Elizabeth Castellanos, Brenda Lopez, MD, Shahdad Saeedi; and the lab researcher Celeste Eng who processed the biospecimens.

NHLBI TOPMed: Study of Asthma Phenotypes & Pharmacogenomic Interactions by Race-Ethnicity (SAPPHIRE_asthma)
The SAPPHIRE cohort was supported by grant funding from the Fund for Henry Ford Hospital, the American Asthma Foundation, and the following institutes of the National Institutes of Health: the National Heart Lung and Blood Institute (R01HL141845, R01HL118267, X01HL134589, R01HL079055), the National Institute of Allergy and Infectious Diseases (R01AI079139, R01AI061774), and the National Institute of Diabetes and Digestive and Kidney Diseases (R01DK113003, R01DK064695).

NHLBI TOPMed: Genetics of Sarcoidosis in African Americans (Sarcoidosis)
Supported by the National Institutes of Health under Grant R01HL113326-05, P30 GM110766-01, and U54GM104938-06.

NHLBI TOPMed: Severe Asthma Research Program (SARP)
The authors acknowledge the contributions of the study coordinators and staff at each of the clinical centers and the Data Coordinating Center as well as all the study participants that have been integral to the success of the NHLBI Severe Asthma Research Program (funded by U10 HL109164, U10 HL109257, U10 HL109146, U10 HL109172, U10 HL109250, U10 HL109250, U10 HL109250, U10 HL109168, U10 HL109152, U10 HL109086).

NHLBI TOPMed: Genome-wide Association Study of Adiposity in Samoans (SAS)
Financial support from the U.S. National Institutes of Health Grants R01-HL093093 and R01HL133040. We acknowledge the assistance of the Samoa Ministry of Health and the Samoa Bureau of Statistics for their guidance and support in the conduct of this study. We thank the local village officials for their help and the participants for their generosity. The following publication describes the origin of the dataset: Hawley NL, Minster RL, Weeks DE, Viali S, Reupena MS, Sun G, Cheng H, Deka R, McGarvey ST. Prevalence of Adiposity and Associated Cardiometabolic Risk Factors in the Samoan Genome-Wide Association Study. Am J Human Biol 2014. 26: 491-501. DOI: 10.1002/jhb.22553. PMID: 24799123.

NHLBI TOPMed: Rare Variants for Hypertension in Taiwan Chinese (THRV)
The Rare Variants for Hypertension in Taiwan Chinese (THRV) is supported by the National Heart, Lung, and Blood Institute (NHLBI) grant (R01HL111249) and its participation in TOPMed is supported by an NHLBI supplement (R01HL111249-04S1). THRV is a collaborative study between Washington University in St. Louis, LA BioMed at Harbor UCLA, University of Texas in Houston, Taichung Veterans General Hospital, Taipei Veterans General Hospital, Tri-Service General Hospital, National

Health Research Institutes, National Taiwan University, and Baylor University. THRV is based (substantially) on the parent SAPPHIRe study, along with additional population-based and hospital-based cohorts. SAPPHIRe was supported by NHLBI grants (U01HL54527, U01HL54498) and Taiwan funds, and the other cohorts were supported by Taiwan funds.

NHLBI TOPMed: The Vanderbilt AF Ablation Registry (VAFAR)
The research reported in this article was supported by grants from the American Heart Association to Dr. Shoemaker (11CRP742009), Dr. Darbar (EIA 0940116N), and grants from the National Institutes of Health (NIH) to Dr. Darbar (R01 HL092217), and Dr. Roden (U19 HL65962, and UL1 RR024975). The project was also supported by a CTSA award (UL1 TR00045) from the National Center for Advancing Translational Sciences. Its contents are solely the responsibility of the authors and do not necessarily represent the official views of the National Center for Advancing Translational Sciences or the NIH.

NHLBI TOPMed: The Vanderbilt Atrial Fibrillation Registry (VU_AF)
The research reported in this article was supported by grants from the American Heart Association to Dr. Darbar (EIA 0940116N), and grants from the National Institutes of Health (NIH) to Dr. Darbar (HL092217), and Dr. Roden (U19 HL65962, and UL1 RR024975). This project was also supported by CTSA award (UL1TR000445) from the National Center for Advancing Translational Sciences. Its contents are solely the responsibility of the authors and do not necessarily represent the official views of the National Center for Advancing Translational Sciences of the NIH.

NHLBI TOPMed: Treatment of Pulmonary Hypertension and Sickle Cell Disease With Sildenafil Therapy (Walk-PHaSST)
We thank Dr. Mark Gladwin and the investigators of the Walk-PHasst study and the patients who participated in the study. We also thanks the walk-PHaSST clinical site team: Albert Einstein College of Medicine: Jane Little and Verlene Davis; Columbia University: Robyn Barst, Erika Rosenzweig, Margaret Lee and Daniela Brady; UCSF Benioff Children's Hospital Oakland: Claudia Morris, Ward Hagar, Lisa Lavrisha, Howard Rosenfeld, and Elliott Vichinsky; Children's Hospital of Pittsburgh of UPMC: Regina McCollum; Hammersmith Hospital, London: Sally Davies, Gaia Mahalingam, Sharon Meehan, Ofelia Lebanto, and Ines Cabrita; Howard University: Victor Gordeuk, Oswaldo Castro, Onyinye Onyekwere,, Alvin Thomas, Gladys Onojobi, Sharmin Diaz, Margaret Fadojutimi-Akinsiku, and Randa Aladdin; Johns Hopkins University: Reda Girgis, Sophie Lanzkron and Durrant Barasa; NHLBI: Mark Gladwin, Greg Kato, James Taylor, Vandana Sachdev, Wynona Coles, Catherine Seamon, Mary Hall, Amy Chi, Cynthia Brenneman, Wen Li, and Erin Smith; University of Colorado: Kathryn Hassell, David Badesch, Deb McCollister and Julie McAfee; University of Illinois at Chicago: Dean Schraufnagel, Robert Molokie, George Kondos, Patricia Cole-Saffold, and Lani Krauz; National Heart & Lung Institute, Imperial College London: Simon Gibbs. Thanks also to the data coordination center team from Rho, Inc.: Nancy Yovetich, Rob Woolson, Jamie Spencer, Christopher Woods, Karen Kesler, Vickie Coble, and Ronald W. Helms. We also thank Dr. Yingze Zhang for directing the Walk-PHasst repository and Dr. Mehdi Nouraie for maintaining the Walk-PHasst database and Dr. Jonathan Goldsmith as a NIH program director for this study. Special thanks to the volunteers who participated in the Walk-PHaSST study. This project was funded with federal funds from the NHLBI, NIH, Department of Health and Human Services, under contract HHSN268200617182C. This study is registered at www.clinicaltrials.gov as NCT00492531. Detail description of the study was published in Blood, 2011 118:855-864, Machado et al "Hospitalization for pain in patients with sickle cell disease treated with sildenafil for elevated TRV and low exercise capacity".

NHLBI TOPMed: Novel Risk Factors for the Development of Atrial Fibrillation in Women (WGHS)

The WGHS is supported by the National Heart, Lung, and Blood Institute (HL043851 and HL080467) and the National Cancer Institute (CA047988 and UM1CA182913). The most recent cardiovascular endpoints were supported by ARRA funding HL099355.

NHLBI TOPMed: Women's Health Initiative (WHI)
The WHI program is funded by the National Heart, Lung, and Blood Institute, National Institutes of Health, U.S. Department of Health and Human Services through contracts HHSN268201600018C, HHSN268201600001C, HHSN268201600002C, HHSN268201600003C, and HHSN268201600004C. This manuscript was prepared in collaboration with investigators of the WHI, and has been reviewed and/or approved by the Women's Health Initiative (WHI). The short list of WHI investigators can be found at https://www.whi.org/researchers/Documents%20%20Write%20a%20Paper/WHI%20Investigator%20Short%20List.pdf.

Other Acknowledgements

Rebecca NA Keener was supported in part by NIH/NIGMS grant 5K12GM123914.
Tyne W Miller-Fleming was supported in part by grant(s) T32 HG008341.
Marios NA Arvanitis was supported in part by grant(s) T32HL007227.
Lucas NA Barwick was supported in part by NHLBI Contract HHSN2682016000021.
John NA Blangero was supported in part by grant(s) R01 Hl113323, U01 DK085524, P01 HL045522, R01 MH078143, R01 MH078111; R01 MH083824.
Esteban G Burchard was supported in part by the Sandler Family Foundation, American Asthma Foundation, RWJF Amos Medical Faculty Development Program, Harry Wm. and Diana V. Hind Distinguished Professor in Pharmaceutical Sciences II, and grants U01HL138626, R01HL117004, R01HL128439, R01HL135156, X01HL134589, R01HL141992, R01HL141845, U01HG009080, R01ES015794, R21ES24844, P60MD006902, R01MD010443, RL5GM118984, R56MD013312, R01HD085993, 24RT-0025 and 27IR-0030.
Juan C Celedon was supported in part by grant(s) R01 HL117191, R01 MD011764, R01 HL119952.
Dawn L DeMeo was supported in part by grant(s) PO1 HL114501 and PO1 HL132825.
Stefan Kääb is supported by the DZHK (German Centre for Cardiovascular Research), partner site: Munich Heart Alliance, Munich, Germany, and by the Munich Center of Health Sciences (MC Health) as part of LMUinnovativ.
Laura M Raffield was supported in part by grant(s) T32 HL129982.
Daniel E Weeks was supported in part by grant(s) R01HL093093, R01HL1333040.
L. Keoki NA Williams was supported in part by grant(s) R01AI061774, R01AI079139, R01HL079055, R01HL118267, R01HL141845, R01DK064695, R01DK113003, the American Asthma Foundation, and the Fund for Henry Ford Hospital.
Brian E Cade was supported in part by grant(s) K01-HL135405.
Zhanghua NA Chen was supported in part by grant(s) R00ES027870.
Michael H Cho was supported in part by NHLBI grants R01HL113264, R01HL137927, and R01HL135142.
Joanne E Curran was supported in part by grant(s) R01 Hl113323, U01 DK085524, P01 HL045522, R01 MH078143, R01 MH078111; R01 MH083824.
Eimear E Kenny was supported in part by grant(s) R01HL104608; U01HG009610; R01DK110113; U01HG009080; UM1HG0089001.
Ryan L Minster was supported in part by grant(s) R01HL093093, R01HL1333040.
Marquitta J. White was supported in part by grant(s) K01HL140218.

Scott T Weiss was supported in part by grant(s) R37 HL066289 and P01 HL132825.
Ramachandran S Vasan was supported in part by Contracts NO1-HC-25195, HHSN268201500001I and 75N92019D00031 from the National Heart, Lung and Blood Institute and grant supplement R01 HL092577-06S1; Dr. Vasan is supported in part by the Evans Medical Foundation and the Jay and Louis Coffman Endowment from the Department of Medicine, Boston University School of Medicine.
Moritz F Sinner is supported by the DZHK (German Centre Cardiovascular Research), partner site: Munich Heart Alliance, Munich, Germany
Edwin K Silverman was supported in part by grant(s) U01HL089856, P01HL114501, R01HL147148, R01HL137927.
Wayne H-H Sheu was supported in part by grant(s) MOST 107-2314B-075A-001-MY3.
Susan NA Redline was supported in part by grant(s) R35-HL135818.
Juan M Peralta was supported in part by grant(s) R01 Hl113323, U01 DK085524, P01 HL045522, R01 MH078143, R01 MH078111; R01 MH083824.
Stephen T McGarvey was supported in part by grant(s) R01HL093093, R01HL1333040.
Angel C.Y. Mak was supported in part by grant(s) R01MD010443, U01HG009080, R01HL128439, R01HL135156, R01HL141992, R01HL141845, R01HL117004.
Ruth JF Loos was supported in part by grant(s) R01DK110113; R01 DK107786; R01 HL142302.
Rajesh NA Kumar was supported in part by grant(s) UG1 HL139125, U01HL138626-01A1, HHSN2752011300013C, U19AR06952, R01AI127695.
Frank D Gilliland was supported in part by the Hastings Foundation.
Bruce Gelb was supported in part by grants(s) R01 HL098123.
A. Aviv's current telomere research is funded by the NIH (R01 HL134840 and U01AG066529) and the Norwegian Research Council (NFR) ES562296.
Goncalo NA Abecasis was supported in part by grant(s) HHSN268201800002I, OT1HL14248, U24HG008956.
James G Wilson was supported in part by grant(s) U54GM115428.
Alexander P Reiner was supported in part by grant(s) R01HL130733, R01HL116446.

The Analysis Commons was funded by R01HL131136.

TOPMed Consortium Members:
Namiko Abe, Gonçalo Abecasis, Francois Aguet, Christine Albert, Laura Almasy, Alvaro Alonso, Seth Ament, Peter Anderson, Pramod Anugu, Deborah Applebaum-Bowden, Kristin Ardlie, Dan Arking, Donna K Arnett, Allison Ashley-Koch, Stella Aslibekyan, Tim Assimes, Paul Auer, Dimitrios Avramopoulos, John Barnard, Kathleen Barnes, R. Graham Barr, Emily Barron-Casella, Lucas Barwick, Terri Beaty, Gerald Beck, Diane Becker, Lewis Becker, Rebecca Beer, Amber Beitelshees, Emelia Benjamin, Takis Benos, Marcos Bezerra, Larry Bielak, Joshua Bis, Thomas Blackwell, John Blangero, Eric Boerwinkle, Donald W. Bowden, Russell Bowler, Jennifer Brody, Ulrich Broeckel, Jai Broome, Karen Bunting, Esteban Burchard, Carlos Bustamante, Erin Buth, Brian Cade, Jonathan Cardwell, Vincent Carey, Cara Carty, Richard Casaburi, James Casella, Peter Castaldi, Mark Chaffin, Christy Chang, Yi-Cheng Chang, Daniel Chasman, Sameer Chavan, Bo-Juen Chen, Wei-Min Chen, Yii-Der Ida Chen, Michael Cho, Seung Hoan Choi, Lee-Ming Chuang, Mina Chung, Ren-Hua Chung, Clary Clish, Suzy Comhair, Matthew Conomos, Elaine Cornell, Adolfo Correa, Carolyn Crandall, James Crapo, L. Adrienne Cupples, Joanne Curran, Jeffrey Curtis, Brian Custer, Coleen Damcott, Dawood Darbar, Sayantan Das, Sean David, Colleen Davis, Michelle Daya, Mariza de Andrade, Lisa de las Fuentes, Michael DeBaun, Ranjan Deka, Dawn DeMeo, Scott Devine, Qing Duan, Ravi Duggirala, Jon Peter Durda, Susan Dutcher, Charles Eaton, Lynette Ekunwe, Adel El Boueiz, Patrick Ellinor, Leslie Emery, Serpil Erzurum, Charles Farber, Tasha Fingerlin, Matthew Flickinger, Myriam Fornage, Nora Franceschini, Chris Frazar, Mao Fu, Stephanie M. Fullerton, Lucinda Fulton, Stacey Gabriel, Weiniu Gan, Shanshan Gao, Yan Gao, Margery Gass, Bruce Gelb, Xiaoqi (Priscilla) Geng, Mark Geraci, Soren Germer, Robert Gerszten, Auyon Ghosh, Richard Gibbs, Chris Gignoux, Mark Gladwin, David Glahn, Stephanie Gogarten, Da-Wei Gong, Harald Goring, Sharon Graw, Daniel Grine, C. Charles Gu, Yue Guan, Xiuqing Guo, Namrata Gupta, Jeff Haessler, Michael Hall, Daniel Harris, Nicola L. Hawley, Jiang He, Ben Heavner, Susan Heckbert, Ryan Hernandez, David Herrington, Craig Hersh, Bertha Hidalgo, James Hixson, Brian Hobbs, John Hokanson, Elliott Hong, Karin Hoth, Chao (Agnes) Hsiung, Yi-Jen Hung, Haley Huston, Chii Min Hwu, Marguerite Ryan Irvin, Rebecca Jackson, Deepti Jain, Cashell Jaquish, Min A Jhun, Jill Johnsen, Andrew Johnson, Craig Johnson, Rich Johnston, Kimberly Jones, Hyun Min Kang, Robert Kaplan, Sharon Kardia, Sekar Kathiresan, Shannon Kelly, Eimear Kenny, Michael Kessler, Alyna Khan, Wonji Kim, Greg Kinney, Barbara Konkle, Charles Kooperberg, Holly Kramer, Christoph Lange, Ethan Lange, Leslie Lange, Cathy Laurie, Cecelia Laurie, Meryl LeBoff, Jiwon Lee, Seunggeun Shawn Lee, Wen-Jane Lee, Jonathon LeFaive, David Levine, Dan Levy, Joshua Lewis, Xiaohui Li, Yun Li, Henry Lin, Honghuang Lin, Keng Han Lin, Xihong Lin, Simin Liu, Yongmei Liu, Yu Liu, Ruth J.F. Loos, Steven Lubitz, Kathryn Lunetta, James Luo, Michael Mahaney, Barry Make, Ani Manichaikul, JoAnn Manson, Lauren Margolin, Lisa Martin, Susan Mathai, Rasika Mathias, Susanne May, Patrick McArdle, Merry-Lynn McDonald, Sean McFarland, Stephen McGarvey, Daniel McGoldrick, Caitlin McHugh, Hao Mei, Luisa Mestroni, Deborah A Meyers, Julie Mikulla, Nancy Min, Mollie Minear, Ryan L Minster, Braxton D. Mitchell, Matt Moll, May E. Montasser, Courtney Montgomery, Arden Moscati, Solomon Musani, Stanford Mwasongwe, Josyf C Mychaleckyj, Girish Nadkarni, Rakhi Naik, Take Naseri, Pradeep Natarajan, Sergei Nekhai, Sarah C. Nelson, Bonnie Neltner, Deborah Nickerson, Kari North, Jeff O'Connell, Tim O'Connor, Heather Ochs-Balcom, David Paik, Nicholette Palmer, James Pankow, George Papanicolaou, Afshin Parsa, Juan Manuel Peralta, Marco Perez, James Perry, Ulrike Peters, Patricia Peyser, Lawrence S Phillips, Toni Pollin, Wendy Post, Julia Powers Becker, Meher Preethi Boorgula, Michael Preuss, Bruce Psaty, Pankaj Qasba, Dandi Qiao, Zhaohui Qin, Nicholas Rafaels, Laura Raffield, Vasan S. Ramachandran , D.C. Rao, Laura Rasmussen-Torvik, Aakrosh Ratan, Susan Redline, Robert Reed, Elizabeth Regan, Alex Reiner, Muagututi‘a Sefuiva Reupena, Ken Rice, Stephen Rich, Dan Roden, Carolina Roselli, Jerome Rotter, Ingo Ruczinski, Pamela Russell, Sarah Ruuska, Kathleen Ryan, Ester Cerdeira Sabino, Danish Saleheen, Shabnam Salimi, Steven Salzberg, Kevin Sandow, Vijay G. Sankaran, Christopher Scheller, Ellen Schmidt, Karen Schwander, David Schwartz, Frank Sciurba, Christine Seidman, Jonathan Seidman, Vivien Sheehan, Stephanie L. Sherman, Amol Shetty, Aniket Shetty, Wayne Hui-Heng Sheu, M. Benjamin Shoemaker, Brian Silver, Edwin Silverman, Jennifer Smith, Josh Smith, Nicholas Smith, Tanja Smith, Sylvia Smoller, Beverly

Snively, Michael Snyder, Tamar Sofer, Nona Sotoodehnia, Adrienne M. Stilp, Garrett Storm, Elizabeth Streeten, Jessica Lasky Su, Yun Ju Sung, Jody Sylvia, Adam Szpiro, Carole Sztalryd, Daniel Taliun, Hua Tang, Margaret Taub, Kent D. Taylor, Matthew Taylor, Simeon Taylor, Marilyn Telen, Timothy A. Thornton, Machiko Threlkeld, Lesley Tinker, David Tirschwell, Sarah Tishkoff, Hemant Tiwari, Catherine Tong, Russell Tracy, Michael Tsai, Dhananjay Vaidya, David Van Den Berg, Peter VandeHaar, Scott Vrieze, Tarik Walker, Robert Wallace, Avram Walts, Fei Fei Wang, Heming Wang, Karol Watson, Daniel E. Weeks, Bruce Weir, Scott Weiss, Lu-Chen Weng, Jennifer Wessel, Cristen Willer, Kayleen Williams, L. Keoki Williams, Carla Wilson, James Wilson, Quenna Wong, Joseph Wu, Huichun Xu, Lisa Yanek, Ivana Yang, Rongze Yang, Norann Zaghloul, Maryam Zekavat, Yingze Zhang, Snow Xueyan Zhao, Wei Zhao, Degui Zhi, Xiang Zhou, Xiaofeng Zhu, Michael Zody, Sebastian Zoellner

TOPMed Hematology and Hemostasis Working Group:
Laura Almasy, Allison Ashley-Koch, Paul Auer, Abraham Aviv, Melissa Bailey, Emily Barron-Casella, David Beame, Lewis Becker, Alexander Bick, Larry Bielak, Thomas Blackwell, John Blangero, Michael Bowers, Jennifer Brody, Pamela Burton, James Casella, Christy Chang, Han Chen, Ming-Huei Chen, Michael Cho, Jason Collins, Matthew Conomos, Adolfo Correa, Rhea Cosentino, Paul de Vries, Jennifer Dean, Pinkal Desai, Qing Duan, Connor Emdin, Nauder Faraday, Jean Feng, Regina Fisher, Annette Fitzpatrick, James Floyd, Santhi Ganesh, Brady Gaynor, Manjit Hanspal, Ross Hardison, Ben Heavner, Craig Hersh, Chani Hodonsky, Michael Honigberg, Steve Horvath, Yao Hu, Jennifer Huffman, Kruthika Raman Iyer, Deepti Jain, Sidd Jaiswal, Jill Johnsen, Andrew Johnson, Brian Joyce, Joel Kaufman, Addison Keely, Rebecca Keener, Shannon Kelly, Derek Klarin, Malgorzata Klauzinska, Barbara Konkle, Charles Kooperberg, Ethan Lange, Leslie Lange, Cathy Laurie, Cecelia Laurie, Brandon Lê, Grace Lee, Claire Leiser, Guillaume Lettre, David Levine, Dan Levy, Joshua Lewis, Bingshan Li, Yun Li, Amarise Little, Shelly-Ann Love, Rasika Mathias, Ravi Mathur, Caitlin McHugh, Karen Miga, Anna Mikhaylova, Julie Mikulla, Braxton D. Mitchell, Alanna C Morrison, Rakhi Naik, Drew Nannini, Pradeep Natarajan, Deborah Nickerson, Jeff O'Connell, Christopher O'Donnell, Nels Olson, Nathan Pankratz, Benedict Paten, James Perry, Steve Pipe, James Pirruccello, Linda Polfus, Bruce Psaty, Jennifer Anne Purnell, Laura Raffield, Alex Reiner, Stephen Rich, Shabnam Salimi, Vijay G. Sankaran, Noah Simon, Nicholas Smith, Adrienne M. Stilp, Hua Tang, Margaret Taub, Marilyn Telen, Timothy A. Thornton, Russell Tracy, Md Mesbah Uddin, Kate Wehr, Joshua Weinstock, Ellen Werner, Marsha Wheeler, Ann Whitney, Eric Whitsel, Kerri Wiggins, Lisa Yanek, Yu-Chung Yang, Maryam Zekavat, Wei Zhao, Xiuwen Zheng, Yinan Zheng

TOPMed Structural Variation Working Group:
Paul Auer, Melissa Bailey, Kathleen Barnes, David Beame, Michael Bowers, Harrison Brand, Ulrich Broeckel, Mark Chaisson, Lavanya Challagundla, Kei Hang Katie Chan, Seung Hoan Choi, Zechen Chong, Bradley Coe, John Cole, Ryan Collins, Matthew Conomos, Michelle Daya, Jennifer Dean, Scott Devine, Evan Eichler, Annette Fitzpatrick, Stephanie Gogarten, C. Charles Gu, Amelia Weber Hall, Ira Hall, Bob Handsaker, Ben Heavner, James Hixson, Deepti Jain, Jicai Jiang, Jill Johnsen, Brian Joyce, Goo Jun, Hyun Min Kang, Addison Keely, Spencer Kelley, Charles Kooperberg, John Lane, Cathy Laurie, Seung-been Steven Lee, David Levine, Dan Levy, Honghuang Lin, Simin Liu, Angel CY Mak, Alisa Manning, Rasika Mathias, Steve McCarroll, Julie Mikulla, Drew Nannini, Giuseppe Narzisi, Deborah Nickerson, Jeff O'Connell, Wanda O'Neal, Grier Page, Nathan Pankratz, Alexandre Pereira, Patricia Peyser, Nathan Pezant, Jennifer Anne Purnell, Aakrosh Ratan, Alex Reiner, Stephen Rich, Ingo Ruczinski, Aniko Sabo, Steven Salzberg, Jenny Schoenberg, Jonathan Seidman, Minseok Seo, Nasa Sinnott Armstrong, Albert Vernon Smith, Vinodh Srinivasasainagendra, Arvis Sulovari, Margaret Taub, Olivia Tintea, Kate Wehr, Joshua Weinstock, Marsha Wheeler, James Wilson, Huichun Xu, Sandy Zellner, Wei Zhao, Yinan Zheng, Degui Zhi, Sebastian Zoellner
